# Standard operating procedure for curation and clinical interpretation of variants in cancer

**DOI:** 10.1186/s13073-019-0687-x

**Published:** 2019-11-29

**Authors:** Arpad M. Danos, Kilannin Krysiak, Erica K. Barnell, Adam C. Coffman, Joshua F. McMichael, Susanna Kiwala, Nicholas C. Spies, Lana M. Sheta, Shahil P. Pema, Lynzey Kujan, Kaitlin A. Clark, Amber Z. Wollam, Shruti Rao, Deborah I. Ritter, Dmitriy Sonkin, Gordana Raca, Wan-Hsin Lin, Cameron J. Grisdale, Raymond H. Kim, Alex H. Wagner, Subha Madhavan, Malachi Griffith, Obi L. Griffith

**Affiliations:** 1 McDonnell Genome Institute, Washington University School of Medicine, St. Louis, MO USA; 2 Department of Pathology and Immunology, Washington University School of Medicine, St. Louis, MO USA; 3 Department of Genetics, Washington University School of Medicine, St. Louis, MO USA; 4 Innovation Center for Biomedical Informatics, Georgetown University, Washington DC, USA; 5 Department of Pediatrics, Texas Children’s Hospital, Baylor College of Medicine, Houston, TX USA; 6 Biometric Research Program, Division of Cancer Treatment and Diagnosis, National Cancer Institute, Rockville, MD USA; 7 Keck School of Medicine, University of Southern California, Los Angeles, California USA; 8 Department of Cancer Biology, Mayo Clinic, Jacksonville, Florida USA; 9 Canada’s Michael Smith Genome Sciences Centre, British Columbia Cancer Agency, Vancouver, BC Canada; 10 Fred A. Litwin Family Center in Genetic Medicine, University Health Network, Toronto, ON Canada; 11 Georgetown Lombardi Comprehensive Cancer Center, Washington DC, USA; 12 Siteman Cancer Center, Washington University School of Medicine, St. Louis, MO USA; 13 Department of Medicine, Washington University School of Medicine, St. Louis, MO USA

**Keywords:** Cancer, Variant, Curation, Standard operating procedure, Knowledgebase

## Abstract

Manually curated variant knowledgebases and their associated knowledge models are serving an increasingly important role in distributing and interpreting variants in cancer. These knowledgebases vary in their level of public accessibility, and the complexity of the models used to capture clinical knowledge. CIViC (Clinical Interpretation of Variants in Cancer - www.civicdb.org) is a fully open, free-to-use cancer variant interpretation knowledgebase that incorporates highly detailed curation of evidence obtained from peer-reviewed publications and meeting abstracts, and currently holds over 6300 Evidence Items for over 2300 variants derived from over 400 genes. CIViC has seen increased adoption by, and also undertaken collaboration with, a wide range of users and organizations involved in research. To enhance CIViC’s clinical value, regular submission to the ClinVar database and pursuit of other regulatory approvals is necessary. For this reason, a formal peer reviewed curation guideline and discussion of the underlying principles of curation is needed. We present here the CIViC knowledge model, standard operating procedures (SOP) for variant curation, and detailed examples to support community-driven curation of cancer variants.

## Introduction

Expansion of pan-cancer sequencing efforts in research and clinical settings has led to a rapid increase in the number of variants that require clinical annotation [1–5]. Substantial computational and manual requirements for variant identification and interpretation have been shown to hinder the development of optimal treatment protocols for patients [6, 7]. These issues highlight the need for normalized clinical classification and representation of relevant variants, as well as open distribution of a standardized cancer variant knowledgebase [8, 9]. Manual curation is an essential part of establishing such a knowledgebase. The harmonization of these curation practices to standard operations procedures (SOPs) would improve quality control and interoperability, facilitating regulatory approval of these curation efforts, especially in the case where these SOPs have been subjected to peer review. SOPs designed to guide a formalized curation effort should outline the structure of the knowledge model, give specific guidance for the curation of each of the model’s elements, and detail how specific guidelines and variant classification systems such as those provided by the American College of Medical Genetics (ACMG), the American Society of Clinical Oncology (ASCO) and the Association for Molecular Pathology (AMP) [10, 11] are utilized during curation. We previously reported the release of the Clinical Interpretation of Variants in Cancer (CIViC) knowledgebase (www.civicdb.org) [12] with only informal online documentation to guide curation. Here, we provide an SOP which will be enforced by all CIViC Editors during moderation of all submitted curation, with the intent to formalize curation into CIViC, and also provide possible motivation for other resources in the field to offer curation SOPs for peer review.

The main text outlines the four principal components of the CIViC knowledgebase (Genes, Variants, Evidence Items, and Assertions) and their associated features. Genes, have collections of associated Variants, where each Variant is supported by at least one literature or conference abstract-derived piece of Evidence (Evidence Item or EID). Multiple Evidence Items describing a single Variant in a specific clincal context can be summarized into a CIViC Assertion. The supplemental materials provide detailed examples and guidelines for curation (see “Curation Practices”) of each element, with emphasis on understanding many of the nuances of cancer variant curation. Common challenges, especially for new CIViC Curators, which could introduce inconsistencies into the database are addressed throughout the SOP. Further details on the CIViC knowledge model, standards and guidelines for curation and moderation, and details on the CIViC project are available in the CIViC help documents (docs.civicdb.org).

## The CIViC knowledge model and key components

### The CIViC knowledge model for clinical variants

**Fig. 1:**
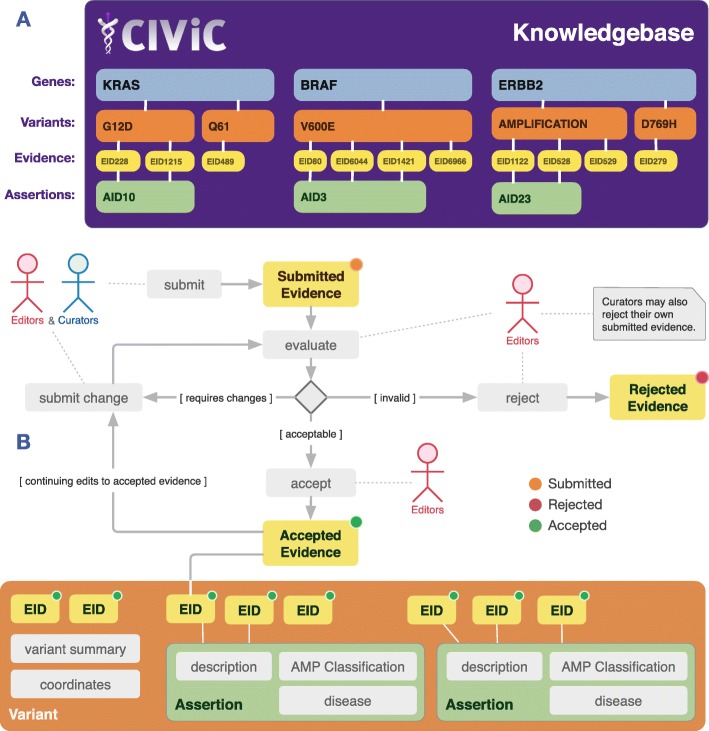
Overview of the CIViC knowledge model for the exploration of existing data (i.e., searching and browsing) and content curation. **a** The CIViC knowledge model consists of four interconnected levels that contribute to the content within CIViC: Genes (blue), Variants (orange), Evidence (yellow), and Assertions (green). Each broadly defined CIViC Variant is associated with a single gene but can have many lines of evidence linking it to clinical relevance. **b** CIViC curation typically begins with the submission of an Evidence Item. Creation of an Evidence Item will automatically generate Gene and Variant records in the knowledgebase if they do not already exist. Once submitted, the Evidence Item undergoes evaluation by expert Editors and (if necessary) revision with ultimate rejection or acceptance. Accepted Evidence Items can be used to build Assertions, which are visualized at the Variant-level. Similar cycles of curation and moderation are employed for all curatable entities in CIViC (e.g., Variant Summaries, Coordinates, Assertions)

The CIViC knowledgebase was built to permit both consumption (i.e., searching, browsing, and downloading) of existing entries as well as curation of new content. The knowledgebase has been organized into a four-level hierarchy: Genes, Variants, Evidence Items, and Assertions (Fig. 1a). Each level has its own knowledge model. All data created using these knowledge models are available through a web interface (www.civicdb.org) and an application programming interface (API, http://docs.civicdb.org/en/latest/api.html).

For content creation, CIViC Curators can add or suggest revisions to curated content at each level (Fig. 1b). Adding content involves submitting new Evidence Items or Assertions that subsequently undergo revision and review by CIViC Editors. Revision of content involves adding or revising the clinical summary and/or its associated features. Once changes are made within the CIViC database, the additions/revisions become visible directly or on a separate revision page depending on the type of submission. Curation is listed as a “submitted” (i.e., pending) until it is accepted by an Editor, who is given power to accept or reject Curator submissions. Curators may reject (but not accept) their own submissions/revisions. Editors are required to fill out a conflict of interest statement (Additional file 1: Figure S1). Further information on Roles in CIViC (Curator, Editor, etc) is in Additional file 1: Table S1, and for a list of User Actions in CIViC see Additional file 1: Table S2.

#### General curation practices

CIViC Curators should avoid directly copying phrases from original sources (including abstracts) for summaries, statements, and comments. This practice prevents plagiarism and copyright infringement for articles with limited public access. Suggested revisions require a comment, generally providing rationale for the change. This allows CIViC Editors to better understand the changes being proposed and facilitates acceptance or further modification. The Source Record Page (Additional file 1: Figure S2) gives an overview of evidence and ability to comment on an evidence source. The Source Suggestion (Additional file 1: Figure S3) offers a rapid and simple means to contribute to CIViC. If a Curator finds inaccuracies or inconsistencies in the database, they should flag such entities to assist Editors in rectifying curation issues, using the flag button at the upper left of CIViC curatable elements (seen in Additional file 1: Figures S1A, S4, S5, and other screenshots in the supplement). Other useful curation features that are found throughout CIViC are described in Additional file 1: Table S3. A workflow for evidence curation is given in Additional file 1: Figure S6.

### Structure and curation of the gene knowledge model

#### Structure of the gene knowledge model

**Fig. 2:**
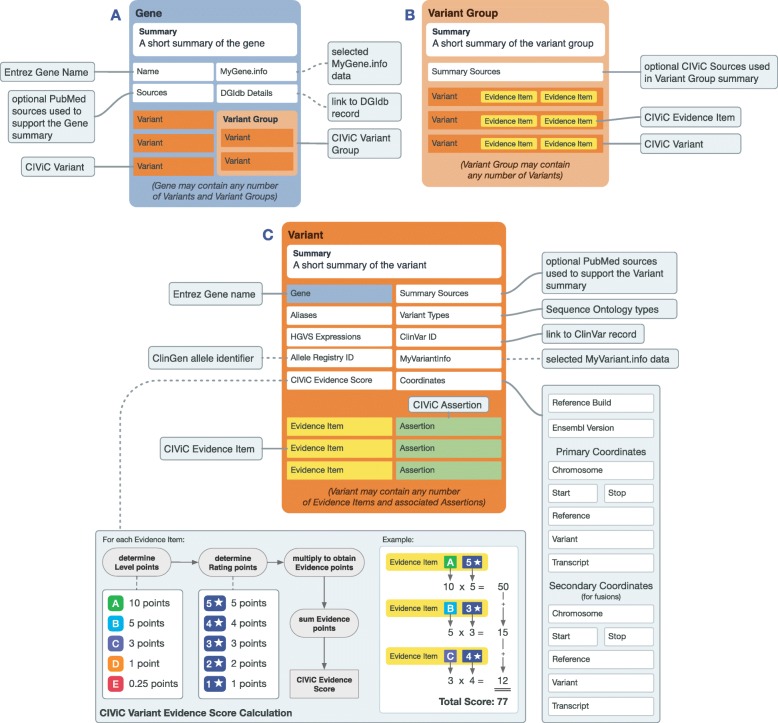
Overview of the Gene and Variant knowledge models and the structure of Variant Groups. The Gene and Variant knowledge models shown above display their associated features (including the Variant Groups feature of Variants) and their origins. Features that are linked to their notes with dotted lines are automatically generated, whenever possible. **a** Gene data (blue box) consists of curated features (Gene Name, Summary, Sources) and auto-generated links to external entities (MyGene.info and DGIdb). Each Gene can be associated with any number of Variants (dark orange box) and Variants can be grouped (light orange box) based on any unifying feature type (e.g., fusions, activating mutations). **b** Variant Group features are outlined by the light orange box. These features include a Summary with Sources and associated Variants. **c** Variant data (dark orange box) includes the Gene Name, Aliases, HGVS Expressions, Variant Evidence Score, Allele Registry ID, Summary Sources, Variant Types, ClinVar IDs, MyVariant.info, and Coordinates. Variants can be associated with CIViC Assertions (green) and Evidence Items (yellow)

The **Gene** knowledge model consists of a Gene Summary which discusses the clinical relevance of the gene in cancer, providing context for the CIViC Variants associated with the Gene, and may specifically mention variants which are prominent in certain cancers. It also contains other structured elements including Gene Name, Gene Summary, external link to The Drug Gene Interaction Database [13–15], useful citations on the overall clinical relevance of the gene, and link-out details from MyGene.info [16] (Fig. 2a). For a Gene record to be created, it must be associated with at least one CIViC Variant.

#### Curating within the gene knowledge model

The CIViC **Gene Name** utilizes the HGNC official symbol as provided by Entrez, primarily those approved by the HUGO Gene Nomenclature Committee (HGNC). Curators must enter a valid Entrez Gene Name (e.g., *TP53*) and should verify the correct entry against the Entrez Gene ID automatically displayed by the CIViC interface. Alternative Gene Names (Aliases/Synonyms) are imported from Entrez and are searchable throughout the database.

A CIViC **Gene Summary** should be created to provide a high-level overview of clinical relevance of cancer variants for the gene. Gene Summaries should focus on emphasizing the clinical relevance from a molecular perspective and should not describe the biological function of the gene unless necessary to contextualize its clinical relevance in cancer. Gene Summaries should include relevant cancer subtypes, specific treatments for the gene’s associated variants, pathway interactions, functional alterations caused by variants in the gene, and normal/abnormal functions of the gene with associated roles in oncogenesis (Additional file 1: Figure S4). A CIViC Gene Summary should generally be limited to one or two paragraphs and cite relevant reviews to further support the gene’s clinical relevance in cancer.

### Structure and curation of the variant knowledge model

#### Structure of the variant knowledge model

A CIViC **Variant** represents any molecular alteration with evidence for clinical relevance in cancer. A new Variant is added to the CIViC database when a new Evidence Item for that Variant is submitted. The CIViC definition for a variant is intentionally broad, to encompass not only simple variation (e.g., SNVs and indels), but also regional variation (e.g., exon mutation), or other types of variation (e.g., expression, amplification, gene fusion, etc.) (Additional file 1: Table S4). Features within the CIViC Variant knowledge model include: Variant Summary, Variant Type, HGVS nomenclature, ClinVar [17] IDs, Variant Evidence Score, representative Variant Coordinates and Transcript, associated Assertions, and external data from MyVariant.info [16] (Fig. 2). Methods for editing Variant information are shown in Additional file 1: Figure S5 and an exemplary Variant entry is shown in Additional file 1: Figure S7.

#### Curating within the variant knowledge model

The **Variant Name** describes the specific variant being interpreted for clinical utility. The Variant Name can be very specific [e.g., *VHL* R176fs (c.528delG)], or can refer to a collection of variants fitting a named category (i.e., *categorical variants* [18]). Examples of categorical CIViC Variants include *KRAS* G12/G13, *EGFR* Exon 20 Insertion, and *PIK3CA* Mutation (Additional file 1: Figure S8). Other Variant Names, including star-allele nomenclature adopted by the pharmacogenetics field (e.g., DPYD*2A; Additional file 1: Figure S9) [19] are also supported. A list of common variant types supported by CIViC are described in Additional file 1: Table S4. When curating this field, the most specific Variant Name described by the source (i.e., publication or abstract) should be used (e.g., *KRAS* G12/G13 rather than *KRAS* Exon 2 Mutation if the paper describes individual variant calls).

**Variant Aliases** are alternative names, descriptions, or identifiers that differ from the primary CIViC Variant Name. These terms are manually curated and are incorporated into the search fields within the CIViC interface. Curators should include one or more aliases such as protein changes on alternative transcripts (e.g., D754Y for *ERBB2* D769Y), dbSNP IDs [20], COSMIC IDs [21] or other identifiers used in the literature to describe the variant.

The **Variant Summary** is a user-defined summary of the clinical relevance of the specific CIViC Variant. The Variant Summary should be a synthesis of the existing Evidence Statements for the CIViC Variant. When curating a Variant Summary, basic information on recurrence rates and biological/functional impact of the variant may be included, but the focus should be on the clinical impact. For Predisposing Variants, any appropriate American College of Medical Genetics (ACMG) evidence codes (ACMG-AMP 2015 criteria [10]) that are not specific to a disease (e.g., PM2) should be recorded with a summary of supporting evidence (Additional file 1: Figure S10). Associated sources (PubMed IDs), including valuable review articles that might not be appropriate for the development of Evidence Items, may be used as references for the Variant Summary.

**Variant Type(s)** are used to classify variants by Sequence Ontology terms [21, 22]. These terms permit advanced searching for categories of variants in the CIViC interface and downstream semantic analyses of CIViC Variants. The most specific term(s) that can be applied to a given Variant should be utilized. Use of the Sequence Ontology browser (http://www.sequenceontology.org/browser/obob.cgi) is recommended to identify appropriate terms. When choosing variant types, selection of multiple terms is supported in order to capture both functional and structural effects of the variant (Additional file 1: Table S5). However, these terms should not be ancestors or descendents of one another, and all selected terms should be descendents of the ‘sequence_variant’ term whenever possible.

The **Variant Evidence Score** sums the Evidence Scores for all Evidence Items associated with the Variant. Evidence Item Scores are calculated by multiplying a weighted Evidence Rating (i.e., one point for each star) by the values assigned to Evidence Level (i.e., A = 10, B = 5, C = 3, D = 1, E = 0.5). The Variant Evidence Score is a relative measure of the total amount of curation in the database for a specific CIViC Variant and does not take into account conflicting evidence.

**Primary and Secondary Coordinates** for each CIViC Variant are manually curated and verified. Each Variant is assigned representative genomic coordinates (Chromosome, Start, Stop, Reference base, and Variant base) for the assigned reference assembly (e.g., GRCh37). Primary Coordinates are generated for all Variants. Secondary Coordinates are utilized for structural variants involving two loci (e.g., fusion variants). Specific guidelines for choosing representative coordinates and transcripts are described below.

##### Choosing representative coordinates

Although multiple genomic changes can often lead to functionally equivalent alterations (e.g., same amino acid change), CIViC uses representative coordinates to provide user-friendly variant context rather than enumerate all possible alterations that could cause the Variant. When choosing a representative CIViC Variant, Curators should use the most common or highly recurrent alteration observed (Additional file 1: Figures S11 and S12). Genomic coordinates are 1-based with left-shifted normalization and include a specified reference assembly (GRCh37 preferred). Based on manually curated representative coordinates, an automated linkout to the ClinGen **Allele Registry** [23] is created. This link provides additional information such as unique and referenceable identifiers and mappings to multiple reference builds and transcripts for every registered variant with links to additional resources (e.g., gnomAD, ClinVar). If the required ClinGen Allele does not yet exist, the Curator should create a ClinGen account and register it.

##### Choosing a representative transcript

Multiple transcripts can often be annotated for a single gene. For this reason, a specific protein coding alteration, resulting from a genomic change, should always be expressed relative to a specific/individual transcript sequence. CIViC representative transcripts use the Ensembl archived version 75 (GRCh37), and Curators should always include the transcript version number (i.e., ENST00000078429.1 instead of ENST00000078429). There is rarely only one correct transcript. Representative transcripts must contain the variant but are otherwise chosen based on priority criteria such as: wide use in the literature, having the longest open reading frame or most exons, containing the most common exons between transcripts, or having the widest genomic coordinates (Additional file 1: Figure S13). These are consistent with Ensembl’s glossary definition of canonical.

The CIViC Variant knowledge model supports The **Human Genome Variation Society (HGVS) Sequence Variant Nomenclature (HGVS Expressions)** to describe sequence variation in genomic, RNA, coding DNA, and protein coordinates [24] as well as curated **ClinVar IDs** for each CIViC Variant. ClinVar IDs and HGVS nomenclature must be entered individually in the Variant editing interface and may capture ClinVar IDs and HGVS entries not described by the representative coordinates. Manual entry is required (e.g., not automatically linked based on representative coordinates) to permit entries for complex or Categorical CIViC Variants and to support alternate transcripts and reference build versions (Additional file 1: Figure S8).

### Structure and curation of the evidence knowledge model

#### Structure of the evidence knowledge model

**Fig. 3:**
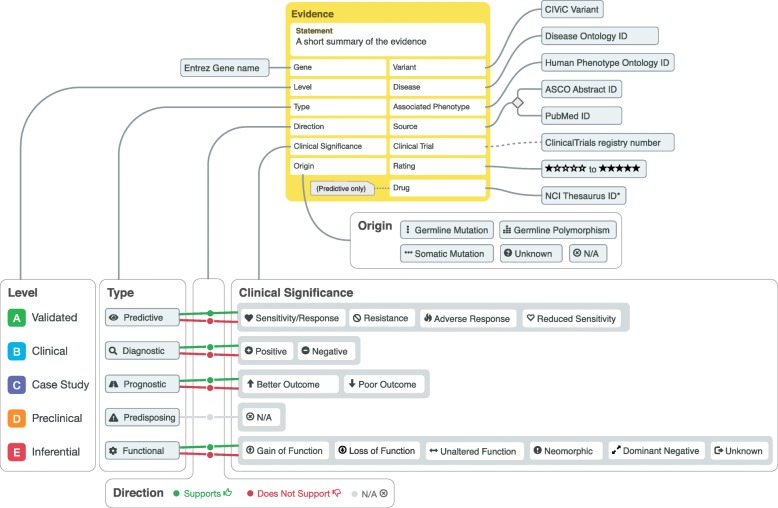
Diagram of the Evidence Item knowledge model. Evidence Items provide a summarized statement about a variant’s implication in clinical oncology in the context of structured data. The knowledge model consists of features (yellow box) that are user-generated and human-readable while leveraging outside ontologies and CIViC-defined fields. Features that are linked to their notes with dotted lines are automatically generated, whenever possible. The Variant Type, Direction, and Clinical Significance features allow Curators to develop complex Evidence Items with nuanced meaning while maintaining queryable structure

At the core of the CIViC knowledge model lies the CIViC **Evidence Item (EIDs)**. EIDs follow a structured knowledge model with 12 required fields (Gene name, Variant Name, Source Type, Variant Origin, Disease, Evidence Statement, Evidence Type, Evidence Level, Evidence Direction, Clinical Significance, and Evidence Rating) with additional optional fields (e.g., Associated Phenotypes). Based on the Evidence Type, additional required or optional fields become available (e.g., Predictive Evidence Types require a Drug Name (which will be linked to an NCIT term when available) and Drug Interaction Type if multiple drugs are involved). Figure 3 describes each field with associated requirements for successful curation and Additional file 1: Figures S14 and S15 show the Evidence Item submission form and display in the Evidence grid.

#### Curating within the evidence knowledge model

A **Gene Name** and **Variant Name** are required for EID submission. The Gene Name field will auto-fill using type-ahead search for genes in the Entrez database or their associated Aliases. The Variant Name will also auto-fill based on existing CIViC Variants. User-defined variants are also permitted if the desired Variant record does not already exist. To prevent redundancy, it is recommended that the Curator browse existing Variant Names for the gene of interest and consider possible variant synonyms before creating a new CIViC Variant.

Each Evidence Item must be associated with a **Source Type** and **Source ID**, which link the EID to the original publication supporting clinical claims. Currently, CIViC accepts publications indexed on PubMed or abstracts published through the American Society of Clinical Oncology (ASCO). If a PubMed Source Type is selected, the Curator can then enter the PubMed ID, which can be verified by comparing the desired source to the abbreviated citation that is automatically generated below the PubMed ID field. If an ASCO Source Type is selected, the ASCO Web ID should be entered into the source ID field. Additionally, **Clinical Trial Registry Number(s)** are automatically linked via the PubMed database, when available.

The **Variant Origin** categorizes the variant based on method of acquisition. Options for this field include: Somatic, Rare Germline, Common Germline, Unknown, or N/A. The Variant Origin should be entered as somatic if the variant is only found in tumor cells (i.e., a somatic variant is only found in a proper subset of non-germ cells/tissue), and is not expected to be inherited or passed to offspring. The Variant Origin is not applicable (N/A) in some circumstances, particularly in variants that involve differences in expression, methylation, or other post-translational modifications (Additional file 1: Figure S16).

The **Disease** field utilizes a term that is known to the Disease Ontology (DO) database [25]. The field will auto-fill based on existing diseases (in the cancer subset of DO) and the most specific disease subtype available should be selected. Only a single Disease term can be associated with an EID. If the clinical evidence associated with the CIViC Variant is implicated in multiple diseases, then multiple Evidence Items should be created. If the disease cannot be identified in the Disease Ontology, the “Could not find disease” box can be selected and a new field will appear that permits free text entry. In this case, it is recommended to submit a request to the Disease Ontology Term Tracker for addition of the missing disease term (http://disease-ontology.org/faq/).

The **Evidence Level** describes the robustness of the study supporting the Evidence Item. Five Evidence Levels are currently available: Validated association (A), Clinical evidence (B), Case study (C), Preclinical evidence (D), and Inferential evidence (E) (Additional file 1: Figures S17 through S21). Validated EIDs (A) have a proven or clinical consensus on the variant association in clinical practice. Typically, these Evidence Items describe Phase III clinical trials, regulatory approvals, or have associated companion diagnostics. Clinical EIDs (B) are typically clinical trials or other primary patient data supporting the clinical association. These EIDs usually include more than 5 patients supporting the claim made in the Evidence Statement. Case studies (C) are individual case reports or small case series. Preclinical evidence (D) is derived from in vivo or in vitro experiments (e.g., mouse models or cell lines) that support clinical claims. Finally, Inferential EIDs (E) indirectly associate the variant to the provided clinical evidence. These can involve hypotheses generated from previous experiments but not yet supported by experimental results. It is possible for an Evidence Source to yield multiple EIDs with different Evidence Levels, for instance Level B and Level C EIDs (See Additional file 1: Figure S22).

The **Evidence Type** refers to the type of clinical (or biological) association described by the Evidence Item’s clinical summary. Five Evidence Types are currently supported: Predictive (i.e., Therapeutic), Diagnostic, Prognostic, Predisposing, and Functional. Each Evidence Type describes the clinical or biological effect a variant has on the following: therapeutic response (Predictive), determining a patient’s diagnosis or disease subtype (Diagnostic), predicting disease progression or patient survival (Prognostic), disease susceptibility (Predisposing), or biological alterations relevant to a cancer phenotype (Functional) (Additional file 1: Figures S23 through S27). Selecting an Evidence Type has implications on available selections for Clinical Significance, as outlined in Fig. 3.

The **Evidence Direction** indicates if the Evidence Statement supports or refutes the clinical significance of an event. The available options include: “Supports” or “Does not support”. Nuanced examples for how to correctly use the Evidence Direction for Predictive Evidence Types are shown in Additional file 1: Table S6 and Additional file 1: Figure S28.

**Clinical Significance** describes how a CIViC Variant is related to a specific clinical interpretation as described in the Evidence Statement. The available options for Clinical Significance depend on the Evidence Type selected for the Evidence Statement. These options are shown in Fig. 3 with details in Additional file 1: Table S7. In brief, they describe the severity or type of treatment response (Predictive), inclusivity or exclusivity of a cancer type or subtype (Diagnostic), the type of outcome (Prognostic), or the type of biological change (Functional). Note that Predisposing Evidence Items may include ACMG-AMP evidence codes [10] in the Evidence Statement; however, they do not directly support an annotated Clinical Significance the way other Evidence Types do, and Predisposing Clinical Significance instead defaults to N/A. This is because most variants will be considered of unknown predisposing significance based on data derived from a single study. CIViC Assertions based on aggregate data handle Predisposing Clinical Significance and are described below.

The **Evidence Rating** is scored on a scale from 1 to 5 stars reflecting the Curator’s confidence in the quality of the summarized evidence (Additional file 1: Figures S29 through S33). This rating depends on a number of factors, including study size, study design, orthogonal validation, and reproducibility. Although the overall publication/study/abstract might be high quality, the Evidence Rating may be low for an Evidence Item referring to a single conclusion in the study that is not well supported. The Evidence Rating therefore does not rate the journal, publication, or Evidence Source itself, but instead evaluates in isolation the components of evidence extracted from the Evidence Source. While this remains a somewhat subjective measure, general best-practices for the Evidence Rating are provided in Additional file 1: Table S8.

The **Evidence Statement** is a brief summary of the clinical implications of the Variant in the context of a specific Disease, Evidence Type and Clinical Significance as described in the cited literature source. An Evidence Statement should synthesize the information from a published study relevant to the clinical association of the CIViC Variant. Evidence Statements should be as brief as possible (typically 1 to 3 sentences), but include sufficient experimental detail to interpret and evaluate the evidence without repeating the original text or using domain-specific acronyms or colloquialisms. Such details include the type of study (e.g., phase, design), controls used, outcomes measured, the number of individuals involved and relevant statistical values (e.g., *p*-values, R^2^, confidence intervals). Data constituting protected health information (PHI) should not be entered in the Evidence Statement field.

For Predictive evidence items, a **Drug Names** field will become available. Multiple drugs can be added to a single Evidence Item, requiring a **Drug Interaction Type** (Combination, Sequential, or Substitutes) that describes the relationship of these drugs in the study. The Drugs and Drug Interaction Types should be explicitly stated in the source supporting the Evidence Item and not inferred by the Curator. Trade names should not be used for Drugs. When available Drug Names are taken from the NCI Thesaurus (https://ncit.nci.nih.gov See Additional file 1: Figure S34). Older drug names/aliases should be referred to by their newer name in the Drug field while mentioning the old and new name in the Evidence Statement to minimize confusion (see Additional file 1: Figure S20).

When additional phenotypes not captured by the Disease field alone are indicated, **Associated Phenotypes** available in the Human Phenotype Ontology (HPO) database [26] can be added to any Evidence Item. Associated Phenotypes should provide additional information beyond what is implied by the Disease field. Phenotypes should be particularly considered for Predisposing Evidence Items whereby the given Variant is associated with a non-binary phenotype or syndrome for a particular genotype.

The last field in the Evidence Item submission form permits free-form text for additional comments about the Evidence Item. For example, Curators can call an Editor’s attention to a particular comment using macro notation (Additional file 1: Table S9). These comments will appear first in the item’s comment thread and will be visible to Editors during review.

### Structure and curation of the Assertion knowledge model

#### Structure of the Assertion knowledge model

**Fig. 4:**
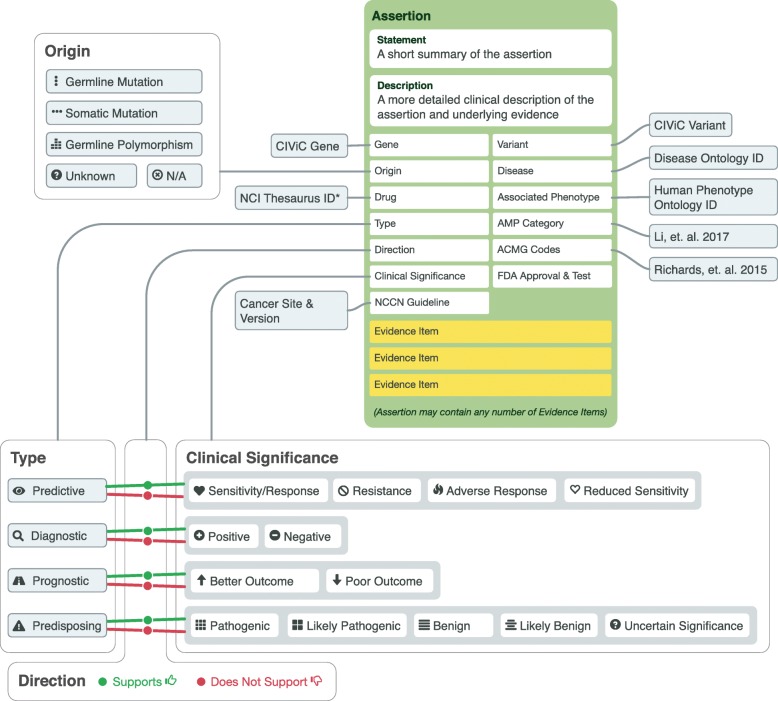
Diagram of knowledge model for CIViC Assertions. Assertions summarize a collection of Evidence Items to make a definitive clinical statement about the Variant in a specific Disease context which incorporates all known data within the knowledgebase. Assertions features (green box) build on the Evidence Item knowledge model to bring together clinical guidelines, public resources, and regulatory approvals relevant to a final variant interpretation. Assertions can be associated with any number of Evidence Items. Like Evidence Items, Assertion Type, Direction, and Clinical Significance can be used to create a specific meaning for the Assertion

The CIViC **Assertion** summarizes the clinical relevance of a CIViC Variant in a specific disease context using a collection of Evidence Items (Fig. 4). Consistent with Evidence Items, Assertions include a Gene, Variant, Variant Origin, Disease, Assertion Type, Assertion Direction, Clinical Significance, Drug (Predictive), Drug Interaction Type (Predictive), and Associated Phenotypes (optional). Fields unique to Assertions include annotation with clinical guidelines such as Association for Molecular Pathology (AMP) Tier and Level from the AMP-ASCO-CAP 2017 guidelines [11], ACMG criteria from the ACMG-AMP 2015 guidelines [10], National Comprehensive Cancer Network (NCCN) guideline/version, and FDA approvals/diagnostics. A short, one sentence Summary and a longer Description of the Assertion are also required for submission. If available, existing Evidence Items should be associated with the Assertion to support the Summary/Description. An Assertion can not be accepted without at least one accepted EID. The Assertion curation form can be found in Additional file 1: Figure S35.

#### Curating within the assertion knowledge model

The **Gene Name** and **Variant Name** for an Assertion have curation constraints. Assertions can only be created for Genes and Variants associated with at least one Evidence Item and are selected from an auto-populated list using type-ahead search. Variant Names are restricted to those associated with the selected Gene. The **Variant Origin** follows the same guidelines as described for Evidence Items (Additional file 1: Figure S36).

The **Disease** associated with the Assertion must already exist within the CIViC database. Only one Disease is permitted for each Assertion. It is recommended that the Disease be as specific as possible while still holding true for all Evidence Items associated with the Assertion (e.g., an Assertion for “non-small cell lung cancer” can be supported by Evidence Items associated with “lung adenocarcinoma” and “non-small cell lung cancer” as well as general disease categories such as “cancer”) (Additional file 1: Figure S37).

**Fig. 5:**
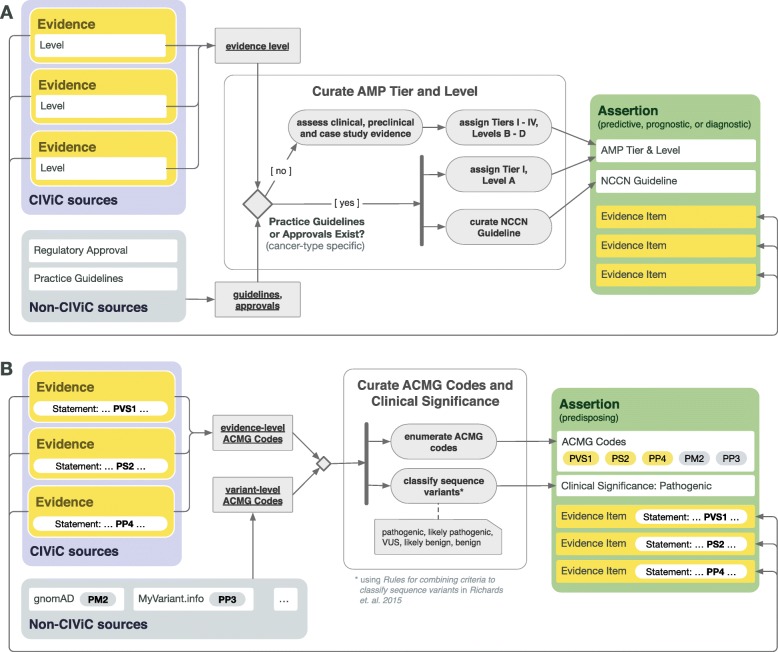
CIViC Assertion development by Assertion Type. CIViC Assertions summarize a collection of Evidence Items which reflect the state of literature for the given variant and disease. **a** For Assertion Types typically associated with somatic variants (Predictive, Prognostic, or Diagnostic), AMP-ASCO-CAP 2017 guidelines are followed to associate the Assertion with an AMP Tier and Level, which involves consideration of practice guidelines as well as regulatory approvals associated with specific drugs, as well as consideration of available clinical evidence in the absence of explicit regulatory or practice guidelines. **b** CIViC Predisposing Assertions utilize ACMG-AMP 2015 guidelines to evaluate the 5-tier classification for a variant in a given disease context, which is supported by a collection of CIViC Evidence Items, along with other data. ACMG evidence codes for an Assertion are supplied by a collection of supporting CIViC Evidence Items (e.g., PP1 from co-segregation data available in a specific publication), and additionally are derived from Variant data (e.g., PM2 from population databases such as gnomAD). ACMG evidence codes are then combined at the Assertion level to generate a disease-specific classification for the Assertion

CIViC currently supports the following **Assertion Types**: Predictive, Diagnostic, Prognostic, and Predisposing. As with the Evidence Item submission form, selecting an Assertion Type will alter available choices for **Clinical Significance**, as outlined in Fig. 4. Options for **Assertion Direction** include “Supports” and “Does not Support”. Predictive, Prognostic or Diagnostic Assertions (Fig. 5a, Additional file 1: Figures S38, S39, S40), utilize the somatic variant interpretation guidelines, providing an AMP-ASCO-CAP Tier (I-IV) and Level (A-D) [11]. Predisposing Assertions (Fig. 5b, Additional file 1: Figure S41) utilize the ACMG-AMP 2015 guideline classifications (Pathogenic, Likely Pathogenic, Likely Benign, Benign and Variant of Unknown Significance), their predicate ACMG evidence codes (i.e., PVS1, PP2, etc), and rules for combining criteria [10], as well as recommended updates [27–29]. Assertions are classified based on the combination of evidence [EIDs and public sources (e.g., gnomAD, CADD)], associated with the Assertion. The Assertion Description should specify the guidelines or classification system used.

Optional descriptive fields for Assertions include **Associated Phenotypes** and **NCCN Guideline(s)/Version(s)** (Additional file 1: Figure S35). If the Variant-Disease association described by the Assertion has a cleared/approved FDA companion diagnostic or a drug with **FDA Regulatory Approval**, then the appropriate box should be checked.

Each Assertion requires a one-sentence **Summary** and a longer, more complete **Description** of the Assertion. The Description is designed to capture special considerations or additional data (e.g., specific treatment regimens, source of ACMG codes) used by the Curator to assemble the Assertion. Important specific details from practice guidelines (e.g. NCCN) should be included in the Summary, including disease stage, and in the case of predictive Assertions, treatment line (e.g., first line, second line, salvage) which practice guidelines recommend.

The **Supporting Evidence** grid allows users to associate Evidence Items with Assertions. This collection of Evidence Items should cover the important clinically relevant findings for the CIViC Variant in the context of a specific cancer. For Predictive Assertions, the collection of Evidence Items should also consider the Drug(s) and their Drug Interaction Type. Assertions do not require Evidence Items for development; however, complete (revised and accepted) Evidence Items must be added to the Assertion before it can be accepted by CIViC Editors (Additional file 1: Figure S42). Minimal Evidence Item requirements for AMP-ASCO-CAP Tier and Level are shown in Additional file 1: Table S10.

## Conclusions

While an earlier publication has introduced the CIViC database [12], no publication has yet offered a comprehensive documentation and SOP of the complex curation practice developed over 4 years of work by the CIViC community worldwide. CIViC has been adopted by a growing number of external organizations. Various gene/disease curation task teams that are part of the ClinGen Somatic Cancer Working Group have now adopted CIViC as their preferred tool for curation of somatic variants in cancer [30]. Data Clients for CIViC now include WikiData, cBioPortal, GeneCards, UCSC Genome Browser, GoldenHelix, MyVariantInfo and many others (A complete list is found here: https://civic.readthedocs.io/en/latest/about/data-clients.html). Citation of CIViC has also been used as supporting evidence in ClinVar submissions (See ClinVar variant 523644). Submission of CIViC Assertions to ClinVar also requires documented curation protocols for variant tiering to obtain 1-star status. Finally, efforts to obtain FDA recognition for the CIViC database and ClinGen Somatic curation process are underway and would benefit from documented curation procedures for CIViC. Therefore, the need for a clear and peer reviewed CIViC SOP is apparent. We hope that this work may build on published SOPs for evaluation of evidence and curation in this space [31, 32] and also function as a template for other data curation efforts as they develop knowledgebases and methods for structured evaluation of evidence, similarly integrating them into the clinical cancer community and regulatory bodies.

Implementation of this SOP will promote standardization of data across CIViC, which is intended as a platform for the generation and dissemination of a large volume of structured data. Utility of large data sets is highly dependent on standardization of the manner in which data is encoded into the format [33, 34]. The free text section of an Evidence Item (EID) - the Evidence Statement - is clearly an area where guidelines for writing and structuring are essential. For instance, when followed, the Predisposing Evidence Statement format (Additional file 1: Figure S26) allows for rapid ascertainment and review of derived ACMG-AMP 2015 codes [10] for the disease and Variant from the particular Evidence Source. Although structured data fields are more constrained, they also require guidelines for standardization of curated data. For example, the Reduced Sensitivity annotation has been restricted to comparison of CIViC Variants under the same treatment scenario (Additional file 1: Table S6), whereas without explicit curation guidelines, this annotation might erroneously be used in scenarios comparing different drug efficacy against the same Variant, causing annotations which inconsistently classify clinical data.

Multiple efforts exist to aggregate, formalize and structure cancer variant data, or provide classification and clinical tiering of somatic or germline variation. Publication of SOPs can help clarify interrelation between these different efforts. For instance, considering the guidance described here for Predictive (Therapeutic) annotations in CIViC (Additional file 1: Table S6 and Figure S28), mappings of these annotations between CIViC and other knowledgebases can be made, such as to Sensitivity and Resistance categories of OncoKB [35] (Additional file 1: Figure S43). Peer reviewed SOPs can facilitate clear comparison between curation efforts and tiering guidelines, and in this particular instance, our SOP offers a standardized structure for CIViC data from initial curation through to the endpoint where collections of CIViC evidence are integrated into Assertions built on ACMG-AMP or AMP-ASCO-CAP guidelines, and can be developed to accommodate other guidelines emerging in the field.

## Supplementary information

**Additional file 1.** Supplementary Materials, contains supplemental figures and tables outlining curation SOP guidelines illustrated by workflow figures, tables, and screenshots from the CIViC interface.

## Data Availability

All data created using these knowledge models are available through a web interface (www.civicdb.org) and an application programming interface (API, http://docs.civicdb.org/en/latest/api.html).

## Abbreviations

ACMG: American College of Medical Genetics
AID: CIViC Assertion identifier
AMP: Association for Molecular Pathology
API: Application Programming Interface
ASCO: American Society of Clinical Oncology
CIViC: Clinical Interpretation of Variants In Cancer
DGIdb: Drug Gene Interaction database
DOID: Disease Ontology identifiers
EID: CIViC Evidence Item identifier
FDA: Food and Drug Administration
HGNC: Human Gene Nomenclature Committee
HPO: Human Phenotype Ontology
NCCN: National Comprehensive Cancer Network
NCI: National Cancer Institute
SOID: Sequence Ontology identifier
SOP: Standard Operating Procedure
