# Supplementary material for: Standard operating procedure for curation and clinical interpretation of variants in cancer
Source: Genome Med. 2019 Nov 29;11:76. doi: 10.1186/s13073-019-0687-x (PMC6883603; doi:10.1186/s13073-019-0687-x)
Supplement: Supplementary file 1 — Additional file 1. Supplementary Materials, contains supplemental figures and tables outlining curation SOP guidelines illustrated by workflow figures, tables, and screenshots from the CIViC interface. [file 13073_2019_687_MOESM1_ESM.pdf]

Additional File 1

Supplementary Materials

Table of Contents

Fig. S1. Conflict of interest statement for CIViC Editors
Fig. S2. Source Page
Fig. S3. Source Suggestion Page

Curating Gene-level entities
Fig. S4. Visualizing and curating the Gene knowledge model

Curating Variant-level entities
Fig. S5. Visualizing and curating the Variant knowledge model
Fig. S6. CIViC Evidence and Variant curation workflow
Fig. S7. Exemplary Variant that has been curated in CIViC
Fig. S8. Example of a Categorical CIViC Variant
Fig. S9. Example of a CIViC Variant that has clinical implications in pharmacogenetics
Fig. S10. Example of a CIViC Variant Summary with inclusion of ACMG-AMP evidence codes
Fig. S11. Defining Variant coordinates for SNVs and small indels
Fig. S12. Defining Variant Coordinates for categorical and large-scale variants
Fig. S13. Choosing a representative transcript

The Evidence Item data model
Fig. S14. Overview of CIViC Evidence Item entry form
Fig. S15. View of the Evidence Grid for a given CIViC Variant
Examples of Variant Origin
Fig. S16. Example of Evidence Item where the Variant Origin is not applicable (N/A)
Examples of Evidence Levels
Fig. S17. Example of a A-Level (Validated) Evidence Item
Fig. S18. Example of a B-Level (Clinical) Evidence Item
Fig. S19. Example of a C-Level (Case Study) Evidence Item
Fig. S20. Example of a D-Level (Preclinical) Evidence Item
Fig. S21. Example of an E-Level (Inferential) Evidence Item
Fig. S22. Level B and C Evidence Items from clinical trial data
Examples of Evidence Types
Fig. S23. Example of a Predictive Evidence Type
Fig. S24. Example of a Diagnostic Evidence Type
Fig. S25. Example of a Prognostic Evidence Type
Fig. S26. Example of a Predisposing Evidence Type
Fig. S27. Example of a Functional Evidence Type
Fig. S28. Interpreting clinical trial data to curate Predictive/Prognostic Evidence Items

Examples of Evidence Rating
Fig. S29. Evidence Item with 5-star Evidence Rating
Fig. S30. Evidence Item with 4-star Evidence Rating
Fig. S31. Evidence Item with 3-star Evidence Rating
Fig. S32. Evidence Item with 2-star Evidence Rating
Fig. S33. Evidence Item with 1-star Evidence Rating
Fig. S34 CIViC Drug Names curation

The Assertion data model
Fig. S35. Overview of CIViC Assertion entry form
Examples of Assertion Variant Origin
Fig. S36. Example of curation of Variant Origin
Examples of Disease in Assertions
Fig. S37. Selection of Disease Type for Assertions
Examples of Assertions
Fig. S38. Predictive Assertion
Fig. S39. Exemplary Prognostic Assertions
Fig. S40. Exemplary Diagnostic Assertions
Fig. S41. Exemplary Predisposing Assertion
Examples of Assertion Supporting Evidence
Fig. S42. Requirements for an Assertion to be accepted
Fig. S43. Comparing CIViC Predictive Clinical Significance to other classification systems
Supplementary Tables
Table S1. Roles in CIViC
Table S2. Activities in CIViC
Table S3. General curation notation for all items within CIViC
Table S4. Examples of Variants supported by the CIViC interface
Table S5. Examples of classification Sequence Ontology Term classifications
Table S6. Use cases for curation of Predictive, Prognostic and Diagnostic Evidence Items with different Evidence Direction, and in different contexts including primary and secondary mutations
Table S7. Definitions of Clinical Significance for all Evidence Types
Table S8. General guidelines and examples for Evidence Rating
Table S9. Markdown and Macros.
Table S10. Minimal Evidence Item requirements for AMP/ASCO/CAP-based CIViC Assertion to be accepted by CIViC Editors

References

Fig. S1. Conflict of interest statement for CIViC Editors

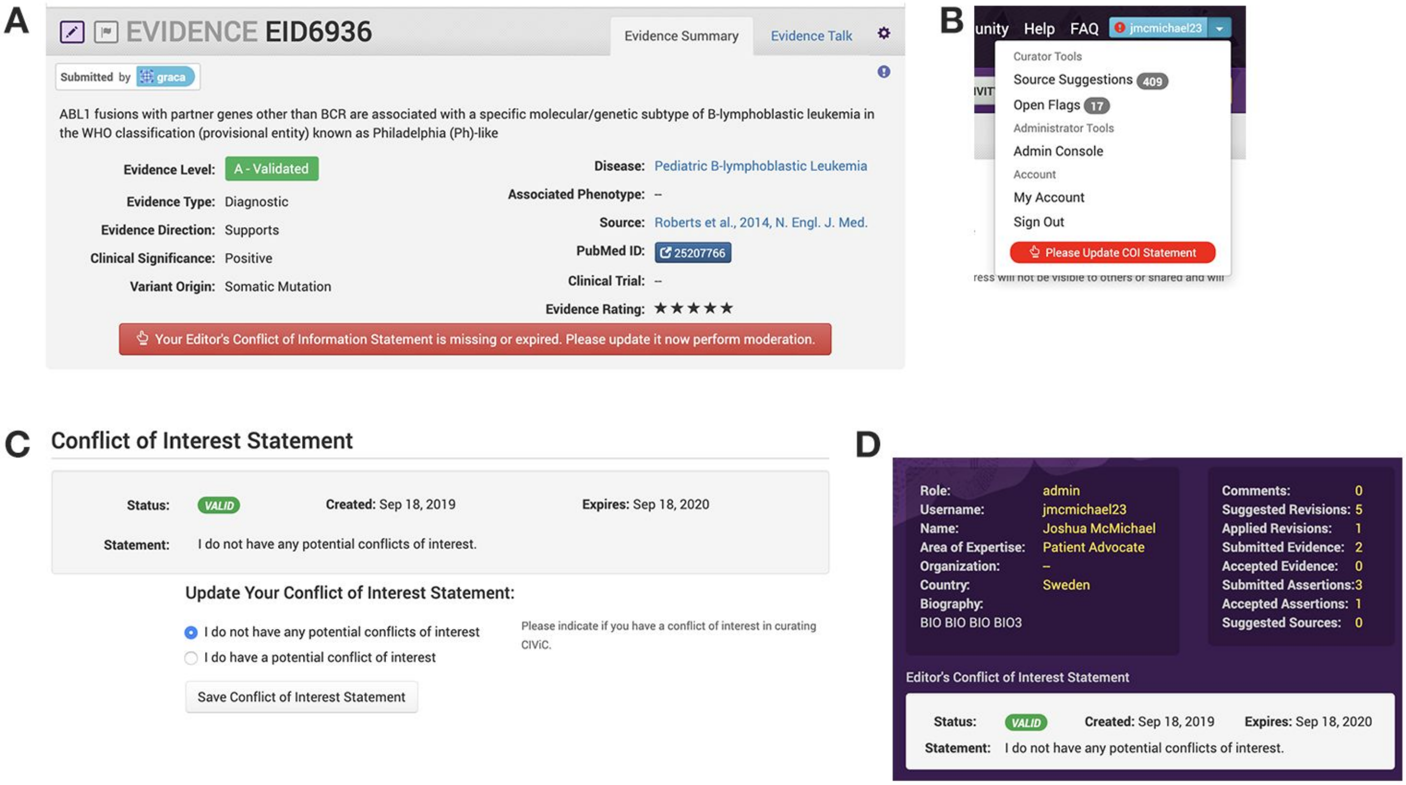

CIViC Editors are required to fill out a statement of potential conflict of interests (COI) once per year. **A:** Editor's view of COI warning statement when COI is not up to date. This statement appears in place of the Accept or Reject buttons used by Editors in the CIViC interface. **B:** A warning message appears in the user menu for a CIViC Editor without an updated conflict of interest (COI) Statement. **C:** Conflict of interest (COI) form. If the Editor has no potential COI, then they select "I do not have any potential conflict of interest". For Editors with a potential conflict of interest, the appropriate button is selected and a free-text statement is written by the Editor which enumerates potential COIs. **D:** The Conflict of Interest (COI) Statement is saved, timestamped and publically viewable for each Editor on their Profile Page.

Fig. S2. Source Page

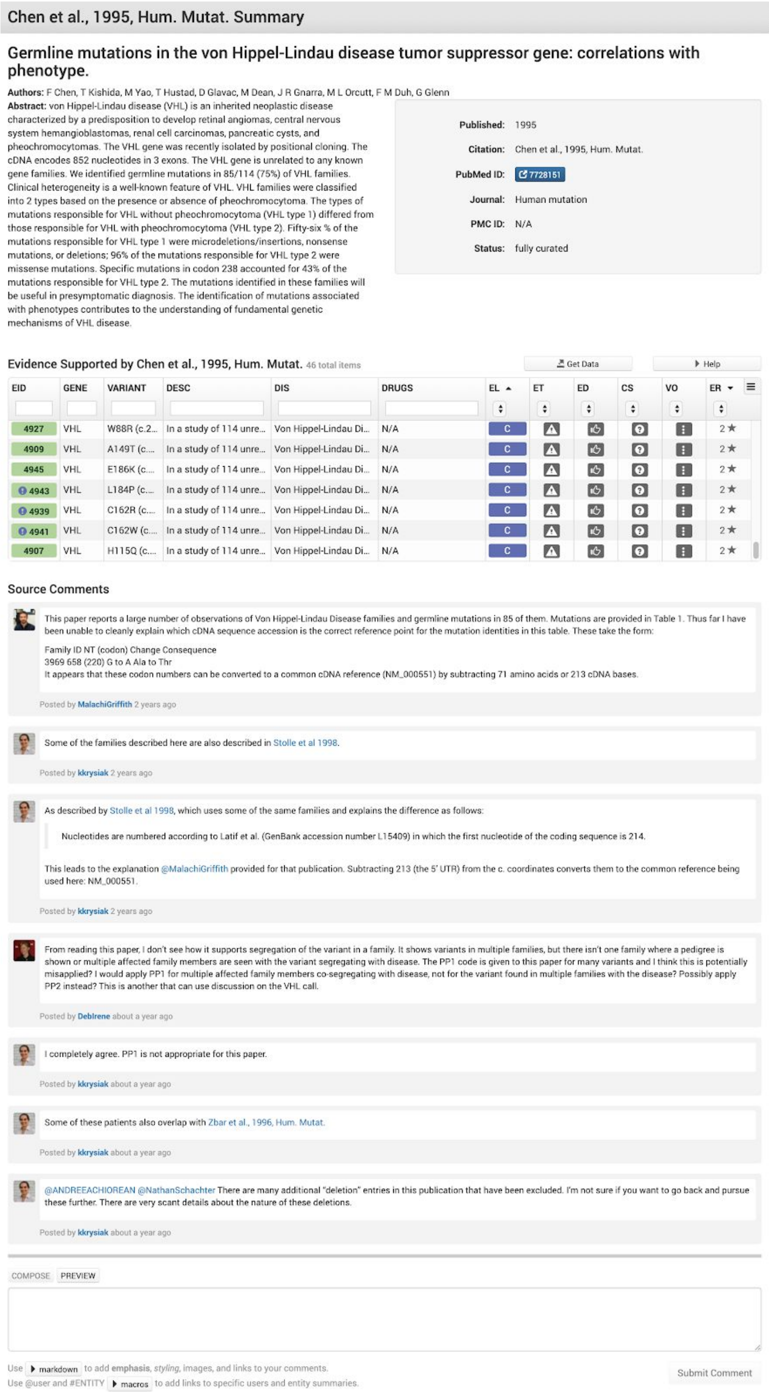

- All curated CIViC Evidence, Assertions, Variant Summaries, and Gene Summaries are based on cited sources from the biomedical literature, currently limited to those publications indexed in PubMed or the ASCO Meeting Library. As soon as any publication is cited in CIViC it appears as a separate Source Record and can be browsed or searched within the CIViC knowledgebase. Each Source Record includes details about the publication as well as a table linking to all CIViC evidence curated for that source.
- Comments can be added to any Source Record at any time. Source comments are a useful place to document general or specific notes to aid current and future curation. For example, the reference sequence used for determining the CIViC Variant's coordinates might be recorded.
- Source comments are a useful place to note study overlap with other literature, for instance if a family reported in a study has been reported in other publications.

Fig. S3. Source Suggestion Page

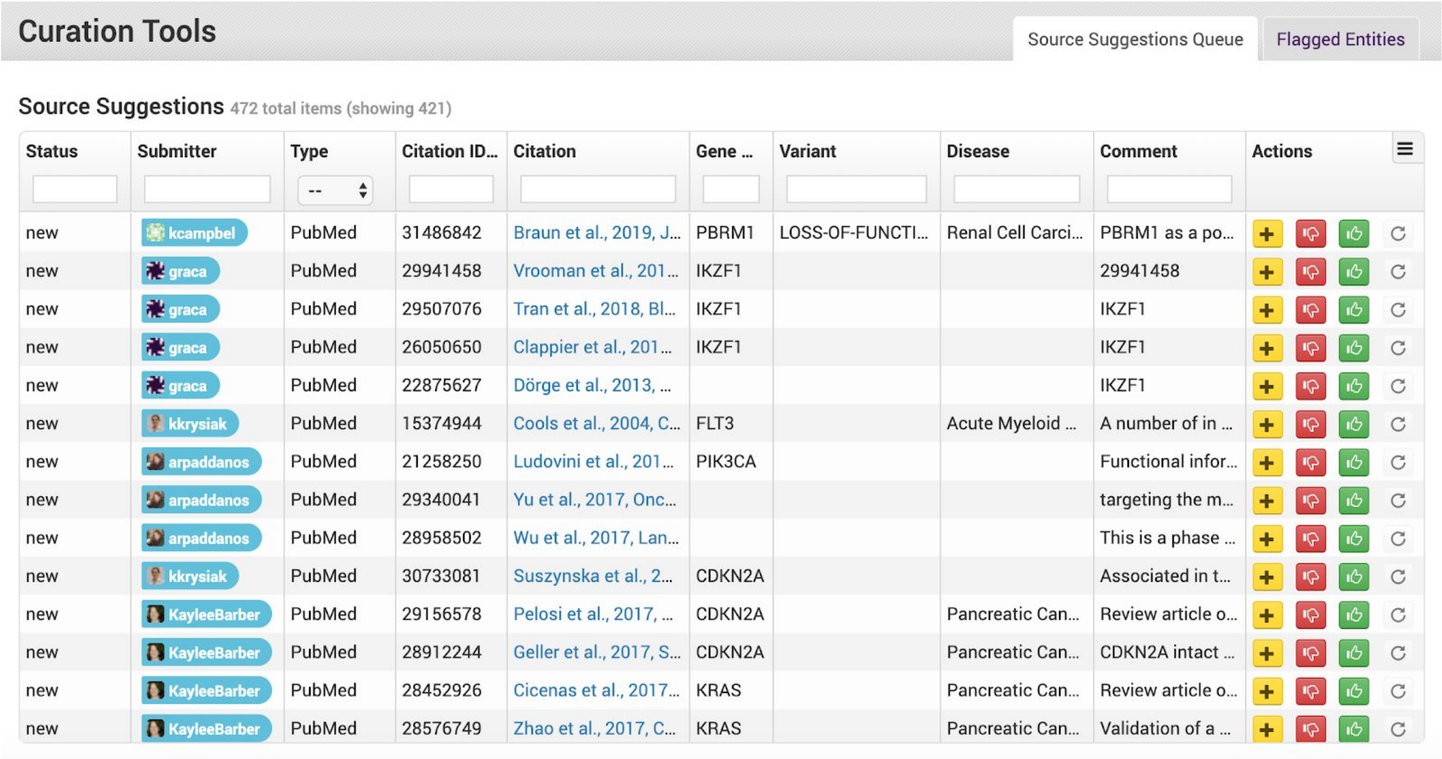

Curation Tools

Source Suggestions Queue | Flagged Entities

Source Suggestions 472 total items (showing 421)

Status	Submitter	Type	Citation ID...	Citation	Gene ...	Variant	Disease	Comment	Actions
		--							
new	kcampbel	PubMed	31486842	Braun et al., 2019, J...	PBRM1	LOSS-OF-FUNCTI...	Renal Cell Carci...	PBRM1 as a po...	
new	graca	PubMed	29941458	Vrooman et al., 201...	IKZF1			29941458	
new	graca	PubMed	29507076	Tran et al., 2018, Bl...	IKZF1			IKZF1	
new	graca	PubMed	26050650	Clappier et al., 201...	IKZF1			IKZF1	
new	graca	PubMed	22875627	Dörge et al., 2013, ...				IKZF1	
new	kkrysiak	PubMed	15374944	Cools et al., 2004, C...	FLT3		Acute Myeloid ...	A number of in ...	
new	arpaddanos	PubMed	21258250	Ludovini et al., 201...	PIK3CA			Functional infor...	
new	arpaddanos	PubMed	29340041	Yu et al., 2017, Onc...				targeting the m...	
new	arpaddanos	PubMed	28958502	Wu et al., 2017, Lan...				This is a phase ...	
new	kkrysiak	PubMed	30733081	Suszynska et al., 2...	CDKN2A			Associated in t...	
new	KayleeBarber	PubMed	29156578	Pelosi et al., 2017, ...	CDKN2A		Pancreatic Can...	Review article o...	
new	KayleeBarber	PubMed	28912244	Geller et al., 2017, S...	CDKN2A		Pancreatic Can...	CDKN2A intact ...	
new	KayleeBarber	PubMed	28452926	Cicenas et al., 2017...	KRAS		Pancreatic Can...	Review article o...	
new	KayleeBarber	PubMed	28576749	Zhao et al., 2017, C...	KRAS		Pancreatic Can...	Validation of a ...	

It is possible to directly submit a “Source Suggestion” for future consideration and curation. This is one of the simplest curation tasks. A CIViC Curator simply accesses the Add -> Source Suggestion form, selects a Source Type (PubMed or ASCO) and then enters a PubMed ID or ASCO Web ID. A comment must be included describing the relevance of this Evidence Source to CIViC and any additional details that might be relevant during subsequent curation of the source. Optionally, a Curator may also “pre-curate” the Gene Name, Variant Name, and Disease described by the source. More than one Source Suggestion can be made for a single source. For example, multiple lines of evidence might be proposed for curation for multiple genes, variants or diseases from the same publication. Once a Source Suggestion has been submitted it can be accessed from the “Source Suggestion Queue” (https://civicdb.org/curation/sources) or from the individual Source Record Page for that source. Each Source Suggestion can be curated using the ‘Add Evidence Item’ action, which activates the Add Evidence form and pre-populates with any Gene Name, Variant Name or Disease details pre-curated for the source. After each Source Suggestion has been curated, Curators can use the “Mark Suggestion as Curated” action. Once all Source Suggestions for a Source have been marked as curated, the Source itself attains a status of “Fully Curated”. Finally, if upon review the Source Suggestion is not determined to be suitable for curation in CIViC, it can be rejected using the “Reject Suggestion” action. CIViC Curators and Editors are encouraged to use Source Comments and Source Suggestions for overall management of curation projects and activities.

Curating Gene-level entities

Fig. S4. Visualizing and curating the Gene knowledge model

This example shows the Gene record for *BRAF* (top). The Gene knowledge model displays the Gene summaries with associated sources, a link to a DGIdb (Griffith et al. 2013; Wagner et al. 2016; Cotto et al. 2018) search for the associated gene, and information pulled from MyGene.info (Xin et al. 2015) (blue box) with a link to additional details and resources. Selection of the purple pencil in the upper left corner activates the Suggested Revision form (bottom). This form allows CIViC Curators to edit the Gene Summary and associated sources and also requires a Revision Description.

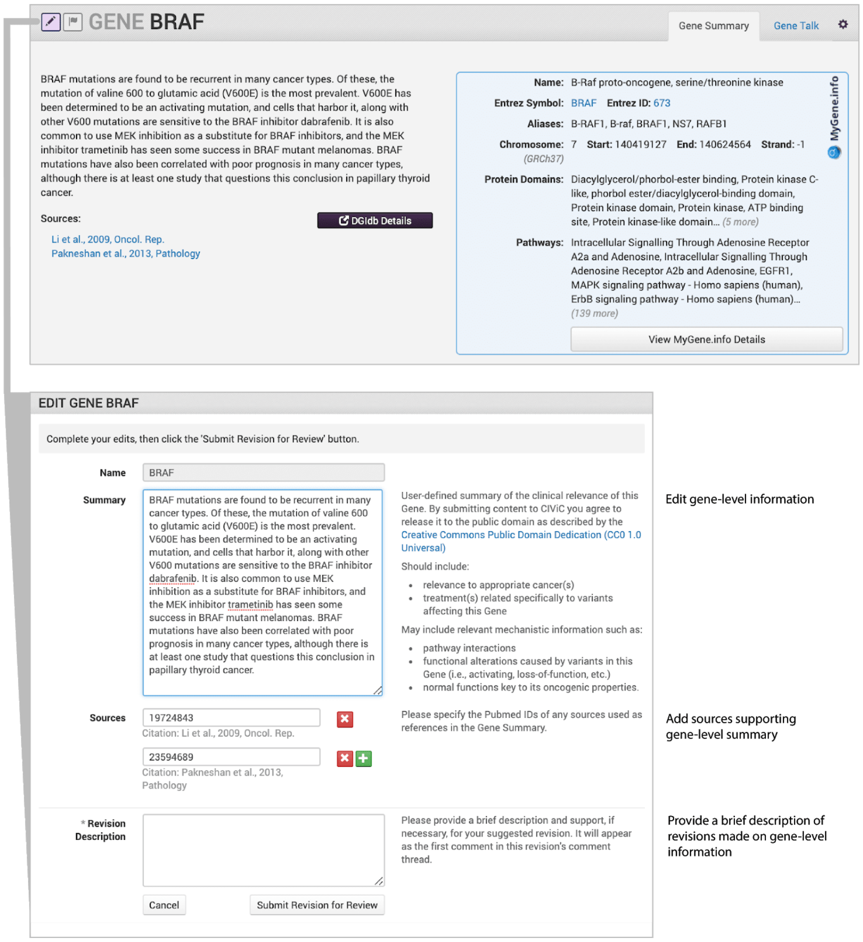

Curation Practices:

- Gene Summaries should include relevant cancer subtypes, specific treatments for the gene's associated variants, pathway interactions, functional alterations caused by variants in the gene, and normal/abnormal functions of the gene with associated roles in oncogenesis.
- A CIViC Gene Summary should generally be limited to one or two paragraphs and cite relevant reviews for a more extensive discussion of clinical relevance of the gene in cancer.
- The sources used for Gene Summaries should be derived from Pubmed and, unlike typical CIViC Evidence Items, may include review articles.
- Instructions for curation are provided in the right column of the Suggested Revision form.

Curating Variant-level entities

Fig. S5. Visualizing and curating the Variant knowledge model

Variants within a gene are displayed in a dynamic list (top left panel) that can be quickly filtered by name. Variant Groups provide user-defined grouping of Variants within and between genes that can be quickly created. The Variant knowledge model (bottom left panel) includes the Variant Name, Aliases, Variant Summary and associated Sources, HGVS Expressions (HGVS Sequence Variant Nomenclature), ClinVar IDs, Variant Types, and Primary Coordinates. Secondary Coordinates (not shown) can also be added to describe genomic context for fusion variants. Selecting any Variant from the list will show the Variant data and associated Evidence Items. Selecting the pencil in the top left corner allows individuals to enter or edit Variant data using a Suggested Revision form (right panel).

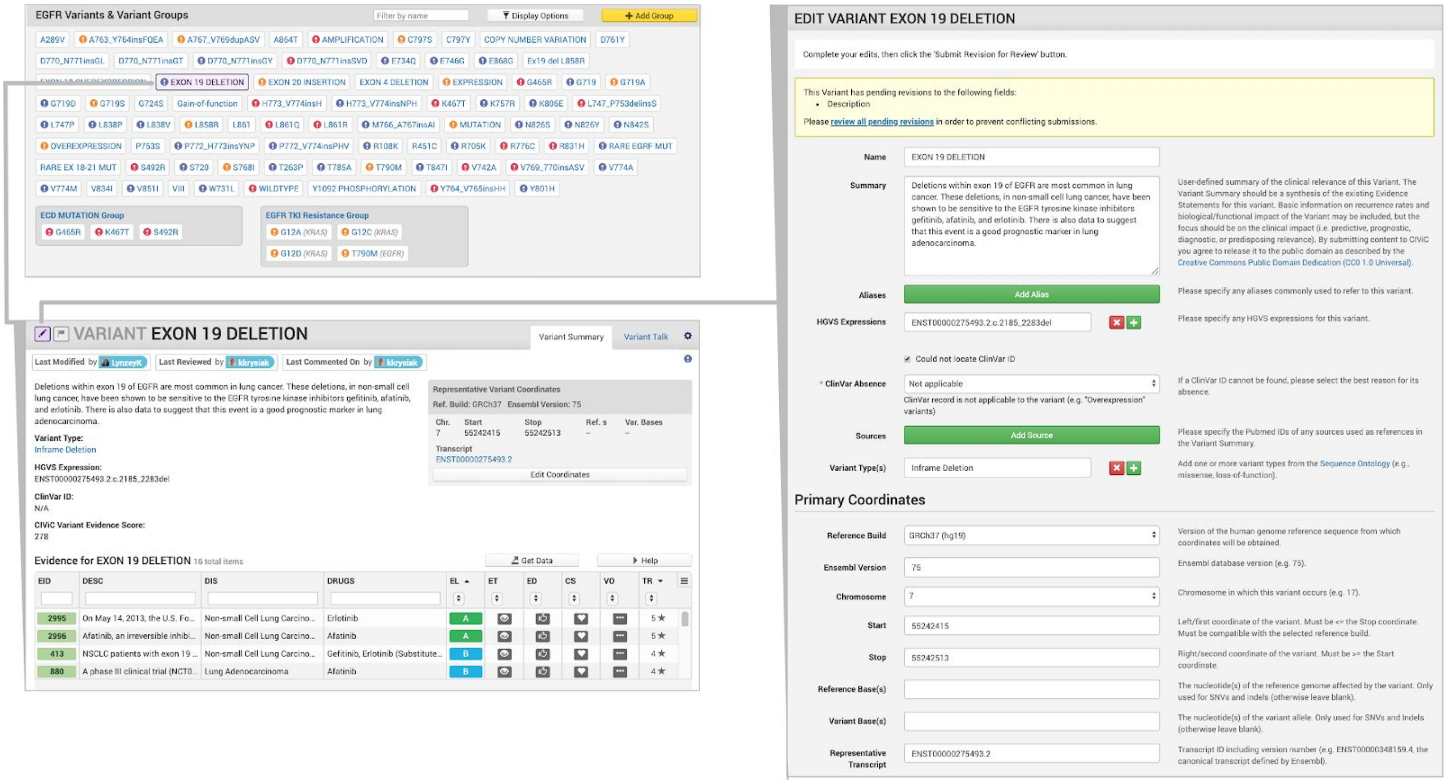

Curation Practices:

- Display options allow users to tailor their CIViC Variant viewing experience by displaying some or all Variants by their status.
- Status icons containing the exclamation point indicate CIViC Variants with active curation involving submitted evidence (orange), pending revisions (purple), or both (red).
- The Evidence Grid for each Variant provides quick filtering and sorting options with status filtering provided by the 3 line icon in the top right.
- Users can download the Evidence Grid by clicking “Get Data”.
- When adding ASCO Abstract Sources, it should be noted that the ASCO Web ID is not the ASCO abstract number. The ASCO Web ID is the identification number found in the URL of the abstract.

Fig. S6. CIViC Evidence and Variant curation workflow

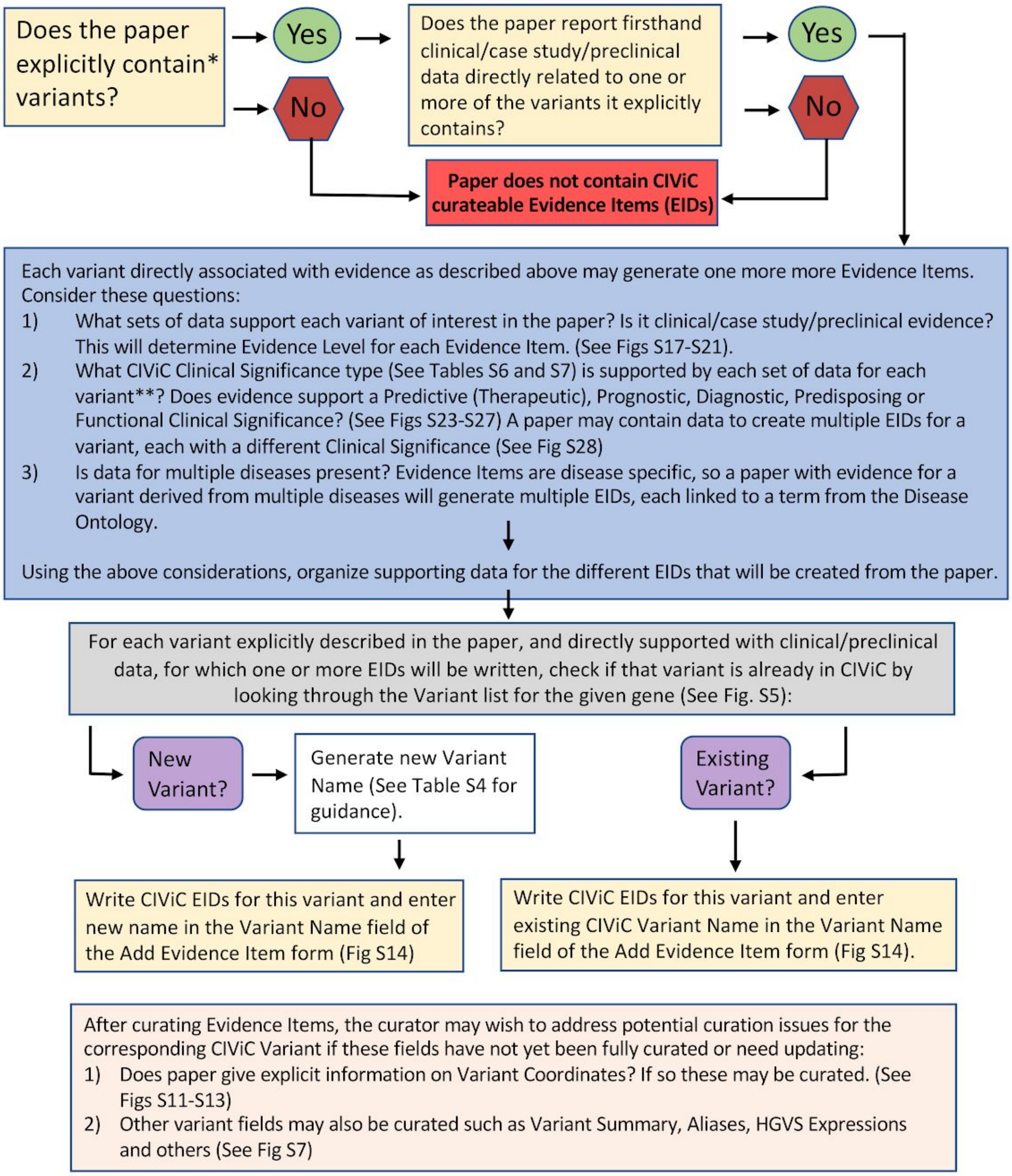

* Explicitly contained (explicitly described) variants in a paper may take on many forms. They may be specific amino acid changes or collections of variants associated with a statistical result (e.g. EGFR Mutation). In some cases the wild type form may be associated with clinical significance (e.g. KRAS in colorectal cancer) resulting in the WILDTYPE variant. See Table S4 for further variant examples. Generally, some papers describe the role of a gene in cancer but do not directly report predictive, prognostic, diagnostic, predisposing or functional evidence for specific variants of that gene, and therefore will not generate CIViC Evidence Items (EIDs).

** Not all clinical data on a cancer variant will be sufficient for creation of an EID. E.g. observation of a somatic variant in a tumor is alone not enough support for any type of CIViC Clinical Significance for a somatic variant EID.

Fig. S7. Exemplary Variant that has been curated in CIViC

This example shows the BCR-ABL T315I variant, which can be used to guide treatment decisions for patients with chronic myeloid leukemia (CML). The Variant Name describes the specific protein change (T315I) that can be observed in conjunction with the pathognomonic fusion (BCR-ABL) for CML. The Variant Summary describes how this variant combination confers resistance to imatinib but sensitivity/response to newer tyrosine kinase inhibitors such as dasatinib and ponatinib. Variant Aliases, HGVS Expression (HGVS Sequence Variant Nomenclature), Variant Types, and ClinVar IDs have all been manually curated with links to external databases, if applicable. The Representative Variant Coordinates describe the most common single base pair change that could result in a T315I variant and these coordinates were used to link to MyVariant.info. The CIViC Variant record currently has 40 submitted Evidence Items that support the Summary of the Variant, many of which are B-level (Clinical) Evidence Items. The status of an Evidence item is indicated by color with submitted in orange or accepted in green.

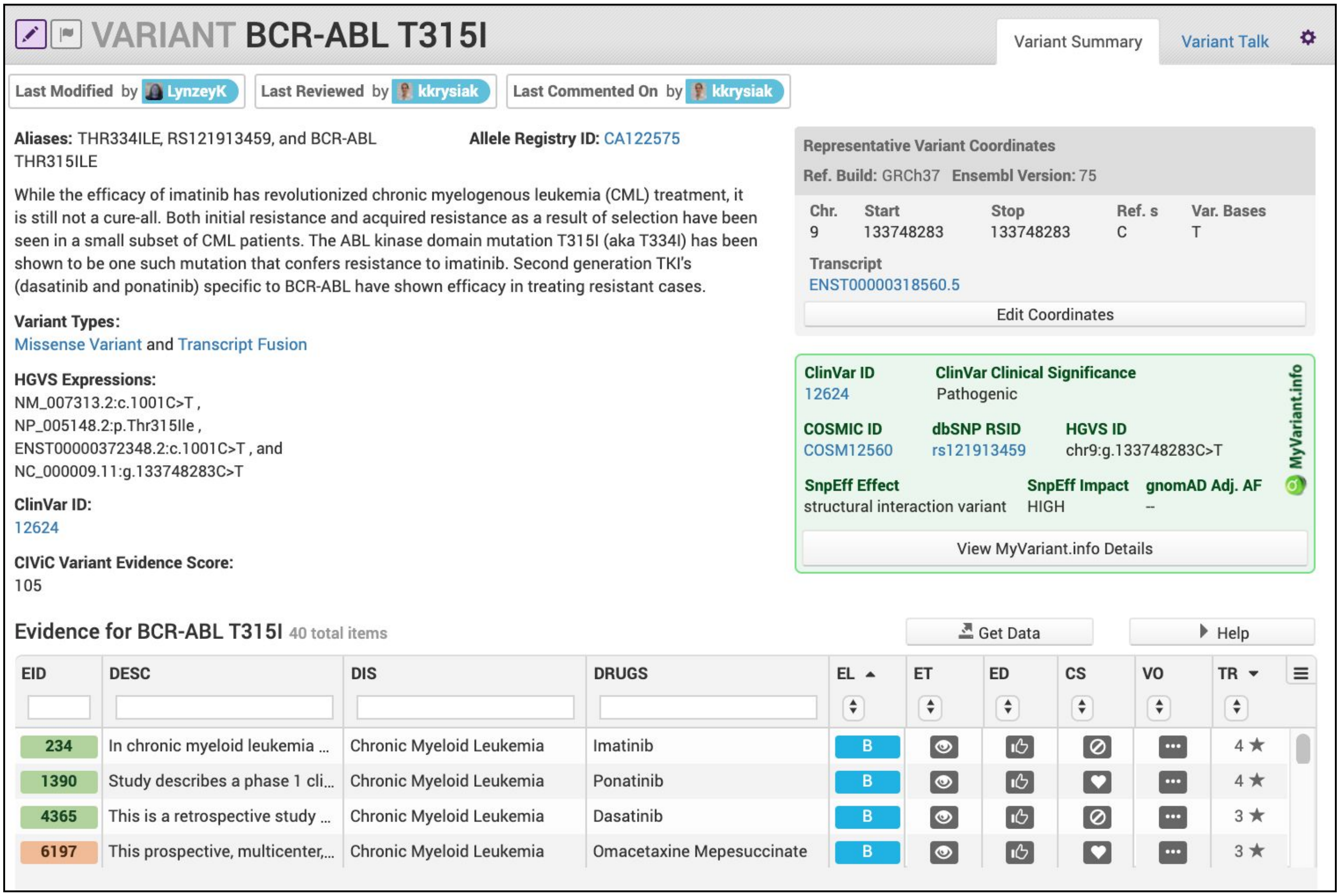

Curation Practices:

- The Variant Summary should attempt to describe all associated and accepted Evidence Items for that Variant. If possible, be specific about the Diseases being referenced or the implicated Drugs.
- Variants can be associated with multiple Variant Types; however, the most specific term should be used and multiple terms related to one another as ancestors or descendants should not be used.
- Note that moderated and accepted Evidence Items (EIDs) are labeled green (EIDs 234, 1390, and 4365) while EIDs that have not yet completed moderation are labeled orange (EID 6197).

Fig. S8. Example of a Categorical CIViC Variant

The image below depicts the *KRAS* - G12/13 Categorical Variant. The term *categorical variant* (sometimes called *bucket variant*) is given to any collection of variants that include some level of ambiguity that prevents assignment of an Evidence Item to a specific CIViC Variant. Due to factors such as the assay used, sample sizes, or experimental design, such non-specific groups of variants are prevalent throughout the literature. For example, studies often look at survival differences between patients with or without a mutation in a given gene to show a significant difference in clinical outcome between populations. However, the clinical outcome is either not significant or not enumerated for the specific variants observed for each individual within the study (See **Fig. S22**). Categorical CIViC Variants are associated with multiple ClinVar entries, if applicable. In the example below, each of the ClinVar IDs links to a specific missense variant that can change *KRAS* at amino acid positions G12 and/or G13.

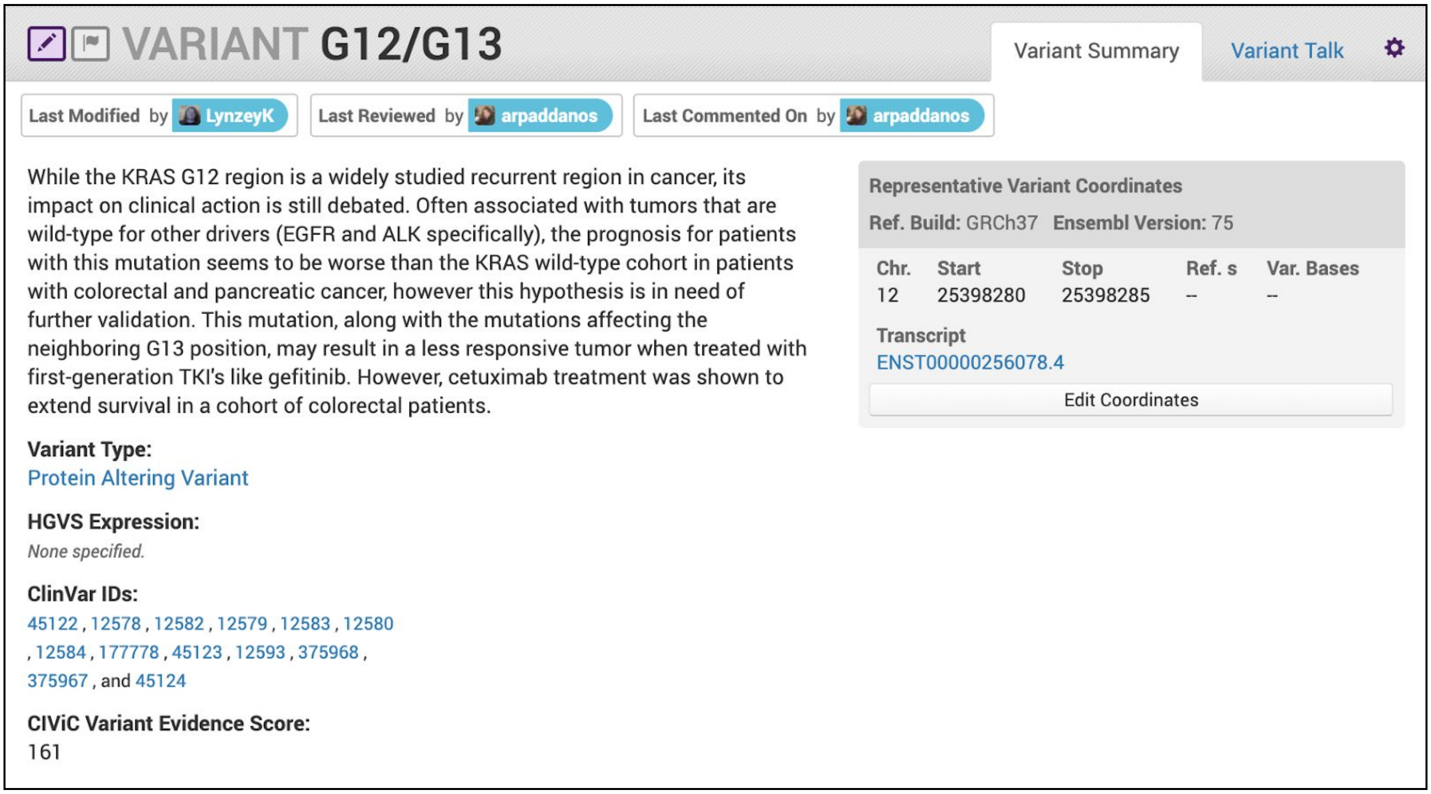

Curation Practices

- Curators can develop Categorical Variants for a variety of reasons. For example, Categorical Variants can be used in the literature to enhance statistical power of the gene or variant being analyzed. Alternatively, the Categorical Variant Name may reflect the assay used (e.g., FISH break-apart probe).
- Often, data used to generate Evidence Items that are associated with Categorical CIViC Variants can also be used to develop additional Evidence Items for individual case studies. For example, a group of individuals with a single categorical mutation (e.g., *EGFR* - Mutation) can be used to generate a B-level Evidence Item that supports response to a therapeutic. Additionally, the specific variants from this cohort (e.g., *EGFR* - G719D and *EGFR* - L861R) can be used to generate C-level Evidence Items to describe therapeutic response for individual cases (See **Fig. S22**).

Fig. S9. Example of a CIViC Variant that has clinical implications in pharmacogenetics

The image below depicts the DPYD*2A Variant, which is a common germline variant that has pharmacogenetic implications. Specifically, the Clinical Pharmacogenomics Implementation Consortium Guidelines does not recommend the use of 5-fluorouracil or capecitabine in patients with homozygous DPYD*2A, *13 or rs67376798 (Robarge et al. 2007).

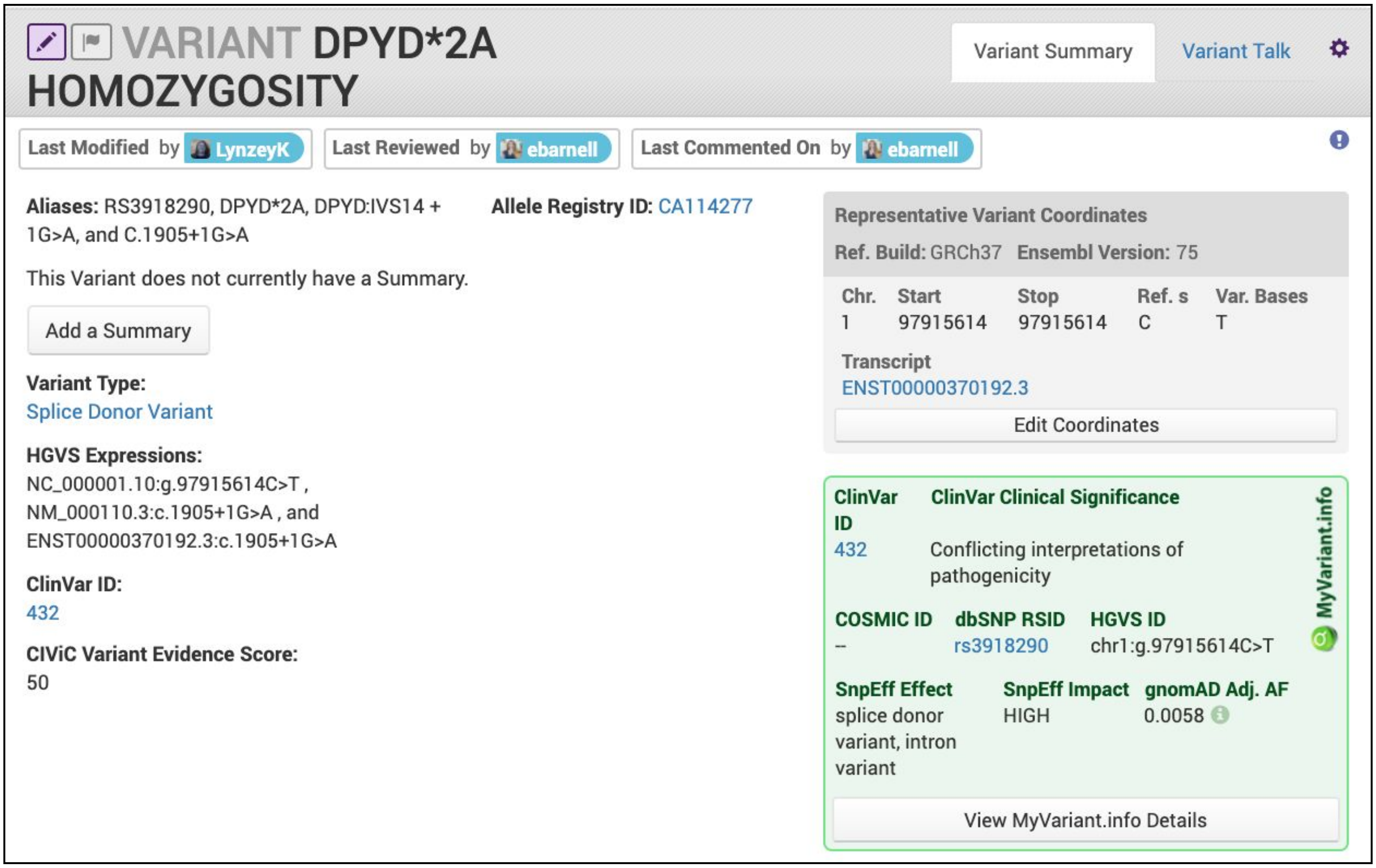

Curation Practices:

- Variant Names should be representative of the name described by the publication unless more current descriptions are applicable. In this case, the older name should be added to the list of Variant Aliases.
- Curators should follow naming guidelines provided by applicable associations. This includes recommendations by the Human Genome Variation Society (HGVS), the Human Variome Project (HVP), and the Human Genome Organization (HUGO).

Fig. S10. Example of a CIViC Variant Summary with inclusion of ACMG-AMP evidence codes

The image below depicts the *TP53* - R175H Variant which includes germline ACMG-AMP evidence codes. In this example, the PM2 code and the specific source of the information used to apply that code are included. This reduces the time needed to evaluate the source of this evidence and whether it warrants updating. Certain evidence codes relating to a variant's population frequency, *in silico* predicted effect, domain location, etc. rely on tools or databases and not specific publications. Such codes relate to the clinical importance of a variant and can appropriately be incorporated at the Variant-level.

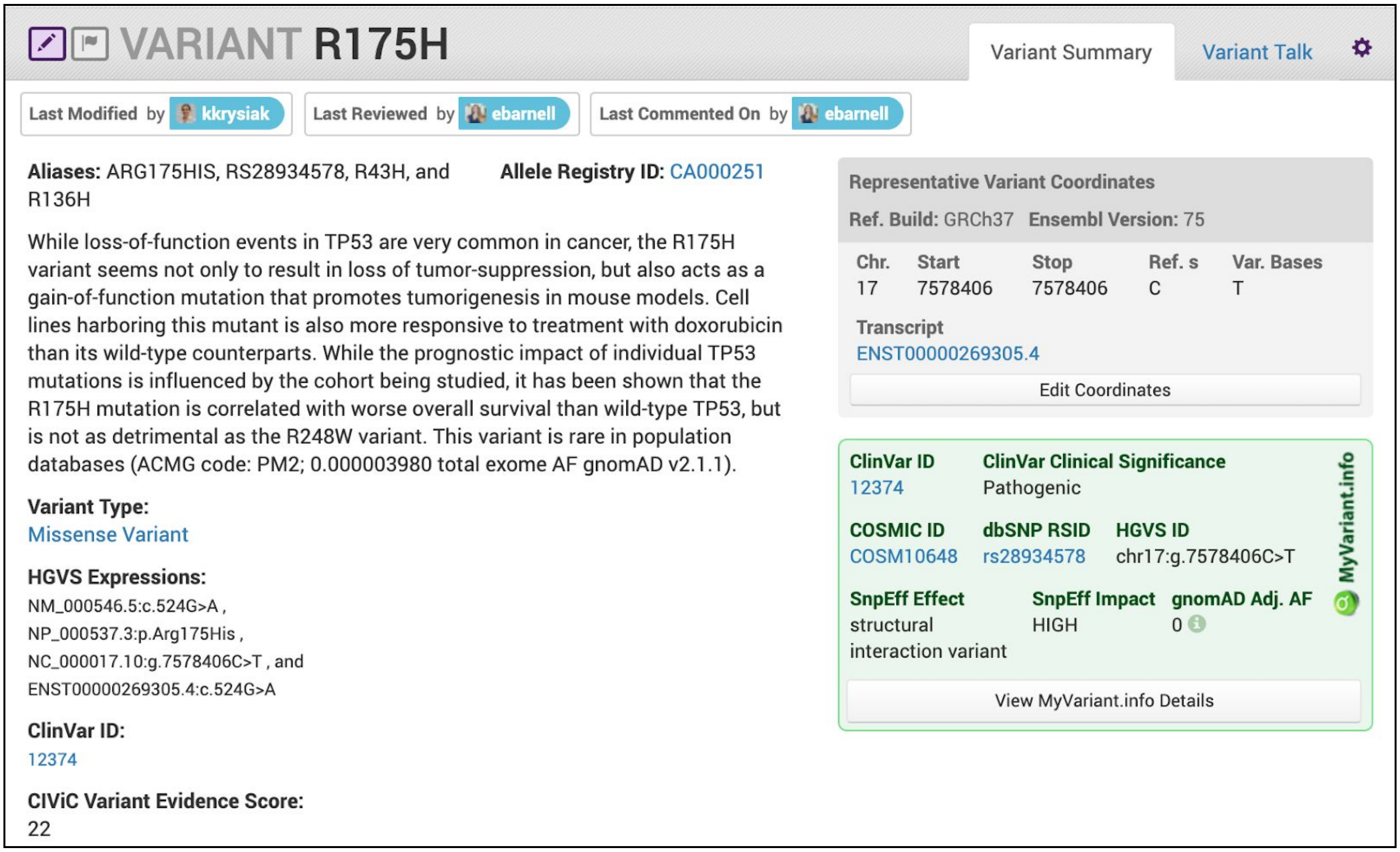

Curation Practices

- ACMG codes, which apply independent of disease type (e.g. population allele frequency PM2), may be listed in the Variant Summary.
- Addition of context-specific ACMG evidence codes may also be captured but should be explicit for circumstances in which the code should, or should not, be used (e.g., PM3 for use only in a recessive disorder).
- Variant-level cautions or warnings of inappropriate codes can be incorporated here (e.g., a warning that PVS1 should not be used for a variant due to an alternative start site after the variant's amino acid).
- Codes which are derived from evidence which has been used to make Evidence Items EIDs for the variant should be listed at the end of the EID Evidence Statements, with a brief sentence justifying the placement of each code (See Fig SSS).

Fig. S11. Defining Variant coordinates for SNVs and small indels

Variant annotation for single nucleotide variants (SNV) and small insertion/deletions (indels) follow a 1-based coordinate system and utilize left-shifted normalization. Reference positions are indicated in green and variant positions in purple. Below, each representation is the text that would be entered into the CIViC Reference Base and Variant Base fields in the Variant Suggested Revision form.

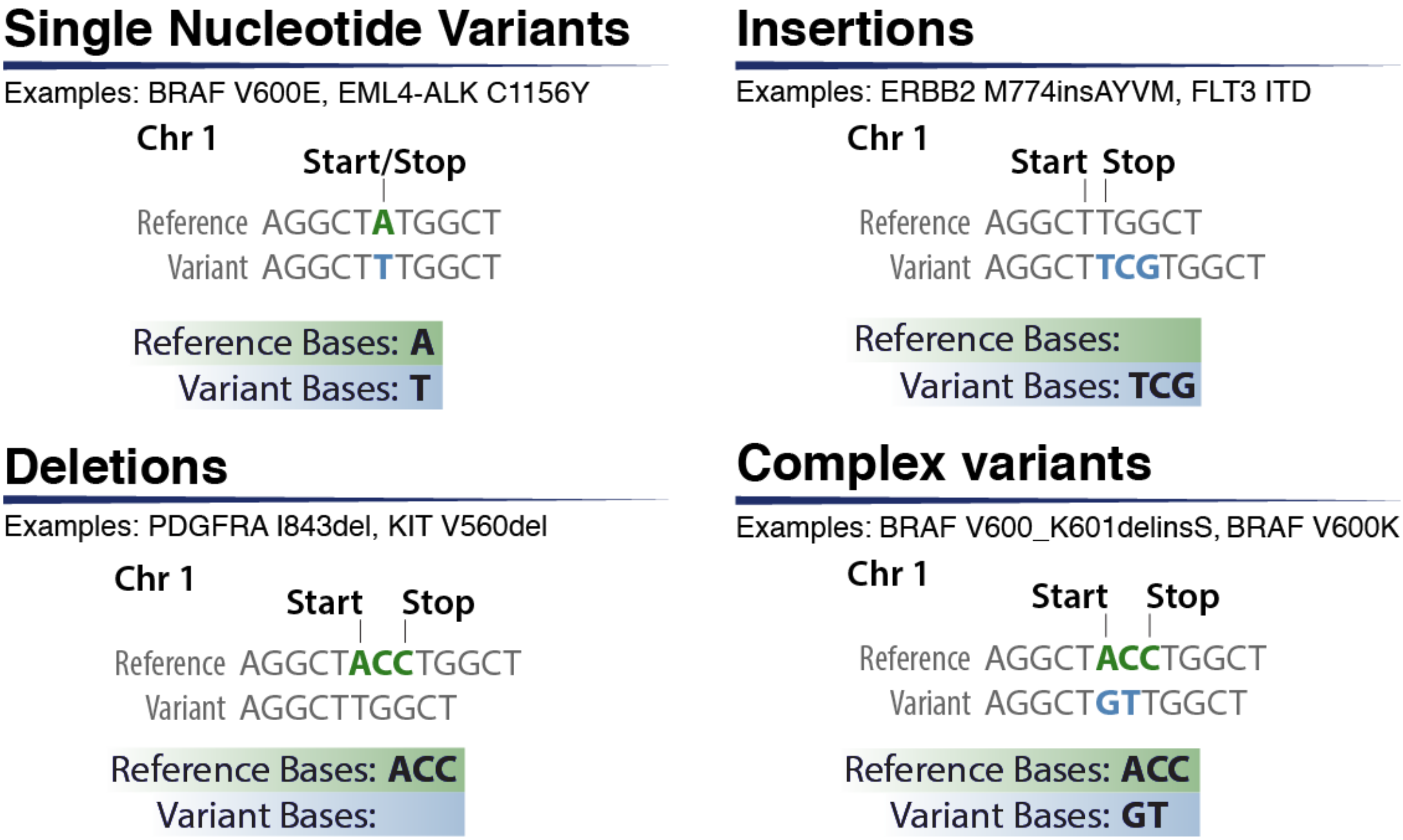

Curation Practices:

- When selecting a representative variant, utilize the most specific and recurrent variant, whenever possible. For example, there are more than 70 insertions listed in COSMIC (Tate et al. 2019) that lead to the highly recurrent *NPM1* W288fs mutation; however, one 4bp insertion (also known as NPM1-A) accounts for more than 90% of all variant entries. Therefore, the coordinates associated with the NPM1-A variant were chosen as the representative coordinates for the *NPM1* W288fs variant in CIViC.
- For complex variants such as SNVs in genes involved in fusions (e.g., EML4-ALK C1156Y), enter the genomic position of the SNV.
- Categorical CIViC Variants involving a single amino acid can be indicated by using the three or two base pairs of the corresponding triplet codon that could result in a SNV at that site (see BRAF V600).

Fig. S12. Defining Variant Coordinates for categorical and large-scale variants

CIViC Variant annotations for large-scale rearrangements or categorical variants (see **Fig. S8**) aim to utilize the minimum genomic space that encompasses the range of variants observed for that gene. For example, although *PIK3CA* amplification can encompass much larger genomic coordinates, the outermost coordinates of the gene *PIK3CA* are used to define the start and stop coordinates. Similarly, Categorical CIViC Variants that contain mutations within the same domain or exon use the outermost coordinates of that domain or exon. Fusion coordinates are based on the closest exon boundary included in the fusion. Although multiple breakpoints can occur, the most common breakpoint is prefered for the representative coordinate.

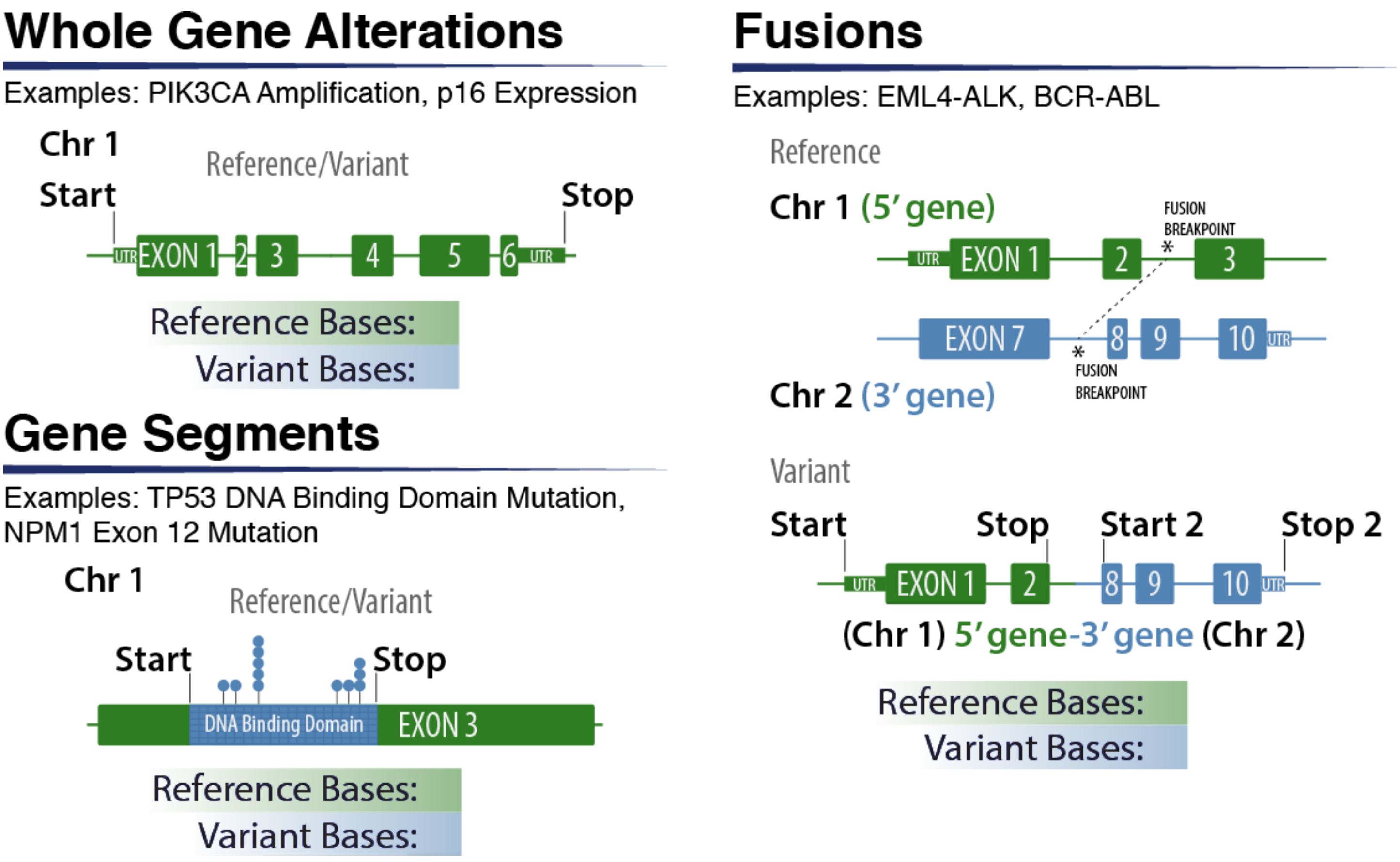

Curation Practices:

- Use coordinates that would encompass the most variants that fit the CIViC Variant name / description to aid others using coordinates to find relevant and similar CIViC Variants.
- For fusions:
  - Names are always written in a 5’ to 3’ order (e.g. 5’ *BCR-ABL* 3’);
  - The Variant is placed under the most ‘important’ gene - e.g. kinase domain - (not repeated under both) which is often the 3’ gene;
  - Coordinates represent the entire putative fusion transcript including start to end of 5’ transcript fusion partner (primary coordinates) and start to end of 3’ transcript fusion partner (secondary coordinates).
  - Both Reference and Variant Bases are left blank.

Fig. S13. Choosing a representative transcript

Genes often have multiple transcript representations. CIViC utilizes Ensembl v75 for transcript annotations. The representative transcript for *WT1* depicted in blue below was chosen because it has the widest outer coordinates with the most common exons compared to the other transcripts depicted in green. This transcript is further highlighted by *** because it is also designated as the “canonical transcript” by Ensembl using select criteria defined in their glossary of terms.

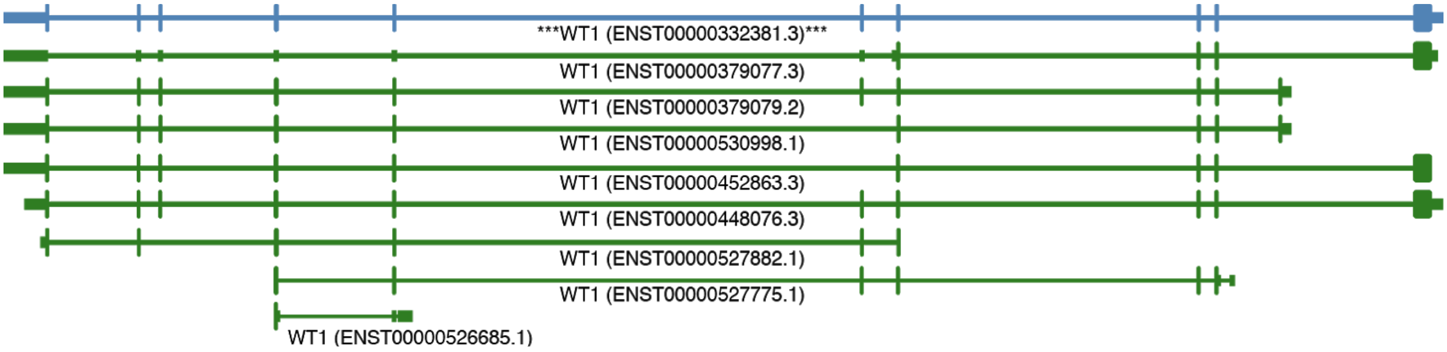

Curation Practices:

- There is no one 'right' answer for representative transcript.
- It must:
    - contain the CIViC Variant (except in rare cases like promoter mutations);
    - be based on an Ensembl transcript and include the transcript version.
- It may:
    - be the transcript with the longest ORF or most exons;
    - be the transcript that contains the 'canonical exons' that are used in many transcripts;
    - be the variant that has the greatest outer coordinates;
    - be the transcript that is widely used in literature;
    - be a transcript that is compatible with interpretation/visualization in the primary literature source.
- An IGV reference transcript file containing Ensembl (v75) transcripts can be obtained here: https://civicdb.org/downloads/Ensembl-v75_build37-hg19_UcscGenePred_CIViC-Genes.ensGene
    - Ensembl canonical transcripts are designated by ***.
- Selection of Representative Transcripts for intronic or regulatory variants follow a similar pattern as protein coding variants.

The Evidence Item data model

Fig. S14. Overview of CIViC Evidence Item entry form

The CIViC evidence entry form reacts to user input with type-ahead search, controlled vocabularies, reactive help text and field layouts updated based on user choices.

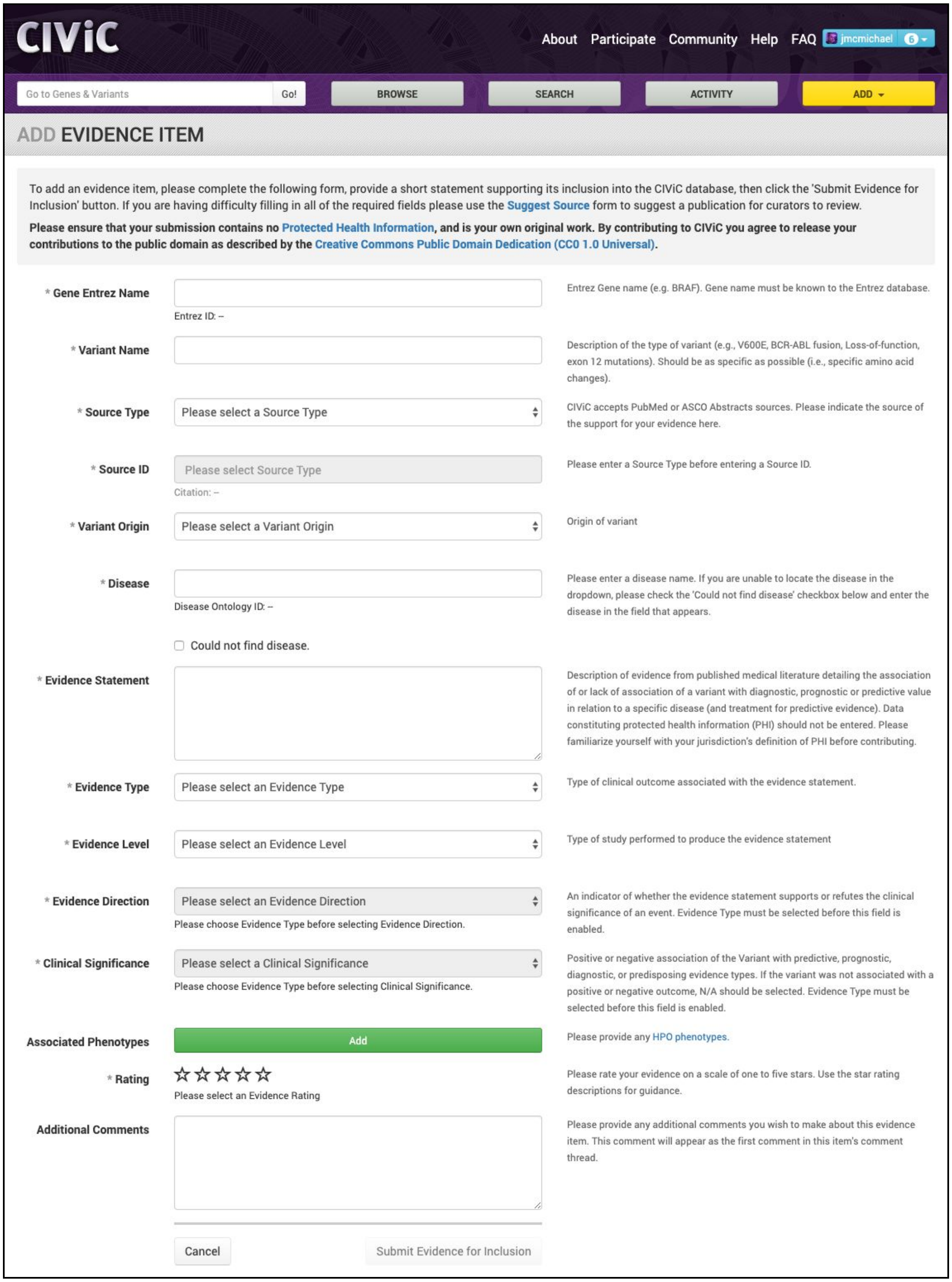

CIViC

About Participate Community Help FAQ jmcmichael 6

Go to Genes & Variants Go! BROWSE SEARCH ACTIVITY ADD

ADD EVIDENCE ITEM

To add an evidence item, please complete the following form, provide a short statement supporting its inclusion into the CIViC database, then click the 'Submit Evidence for Inclusion' button. If you are having difficulty filling in all of the required fields please use the Suggest Source form to suggest a publication for curators to review.

Please ensure that your submission contains no Protected Health Information, and is your own original work. By contributing to CIViC you agree to release your contributions to the public domain as described by the Creative Commons Public Domain Dedication (CC0 1.0 Universal).

Field	Input	Help
* Gene Entrez Name	Entrez ID: --	Entrez Gene name (e.g. BRAF). Gene name must be known to the Entrez database.
* Variant Name		Description of the type of variant (e.g., V600E, BCR-ABL fusion, Loss-of-function, exon 12 mutations). Should be as specific as possible (i.e., specific amino acid changes).
* Source Type	Please select a Source Type	CIViC accepts PubMed or ASCO Abstracts sources. Please indicate the source of the support for your evidence here.
* Source ID	Please select Source Type<br>Citation: --	Please enter a Source Type before entering a Source ID.
* Variant Origin	Please select a Variant Origin	Origin of variant
* Disease	Disease Ontology ID: --<br>Could not find disease.	Please enter a disease name. If you are unable to locate the disease in the dropdown, please check the 'Could not find disease' checkbox below and enter the disease in the field that appears.
* Evidence Statement		Description of evidence from published medical literature detailing the association of or lack of association of a variant with diagnostic, prognostic or predictive value in relation to a specific disease (and treatment for predictive evidence). Data constituting protected health information (PHI) should not be entered. Please familiarize yourself with your jurisdiction's definition of PHI before contributing.
* Evidence Type	Please select an Evidence Type	Type of clinical outcome associated with the evidence statement.
* Evidence Level	Please select an Evidence Level	Type of study performed to produce the evidence statement
* Evidence Direction	Please select an Evidence Direction<br>Please choose Evidence Type before selecting Evidence Direction.	An indicator of whether the evidence statement supports or refutes the clinical significance of an event. Evidence Type must be selected before this field is enabled.
* Clinical Significance	Please select a Clinical Significance<br>Please choose Evidence Type before selecting Clinical Significance.	Positive or negative association of the Variant with predictive, prognostic, diagnostic, or predisposing evidence types. If the variant was not associated with a positive or negative outcome, N/A should be selected. Evidence Type must be selected before this field is enabled.
Associated Phenotypes	Add	Please provide any HPO phenotypes.
* Rating	☆☆☆☆☆<br>Please select an Evidence Rating	Please rate your evidence on a scale of one to five stars. Use the star rating descriptions for guidance.
Additional Comments		Please provide any additional comments you wish to make about this evidence item. This comment will appear as the first comment in this item's comment thread.

Cancel Submit Evidence for Inclusion

Fig. S15. View of the Evidence Grid for a given CIViC Variant

Evidence for a given CIViC Variant is displayed in an Evidence Grid. This grid is highly customizable with each field allowing for text-based searching (e.g., EID, Description), entity filtering (e.g., Evidence Level), and sorting. A quick overview of the variety of evidence supporting a Variant can be quickly obtained using the combination of colors and icons (see “Help” for full legend). The current curation state of the Evidence Item including status and any pending revisions are indicated by the color of the EID and the presence of the Pending Revisions icon. This curation state (Submitted vs. Accepted) should be considered when viewing data for any Variant in CIViC. The default view governing which combination of Accepted and Submitted evidence is shown can be changed by using the “Grid Options Menu” (top right). The contents of the Evidence Grid can be downloaded using the “Get Data” or “Grid Options Menu.”

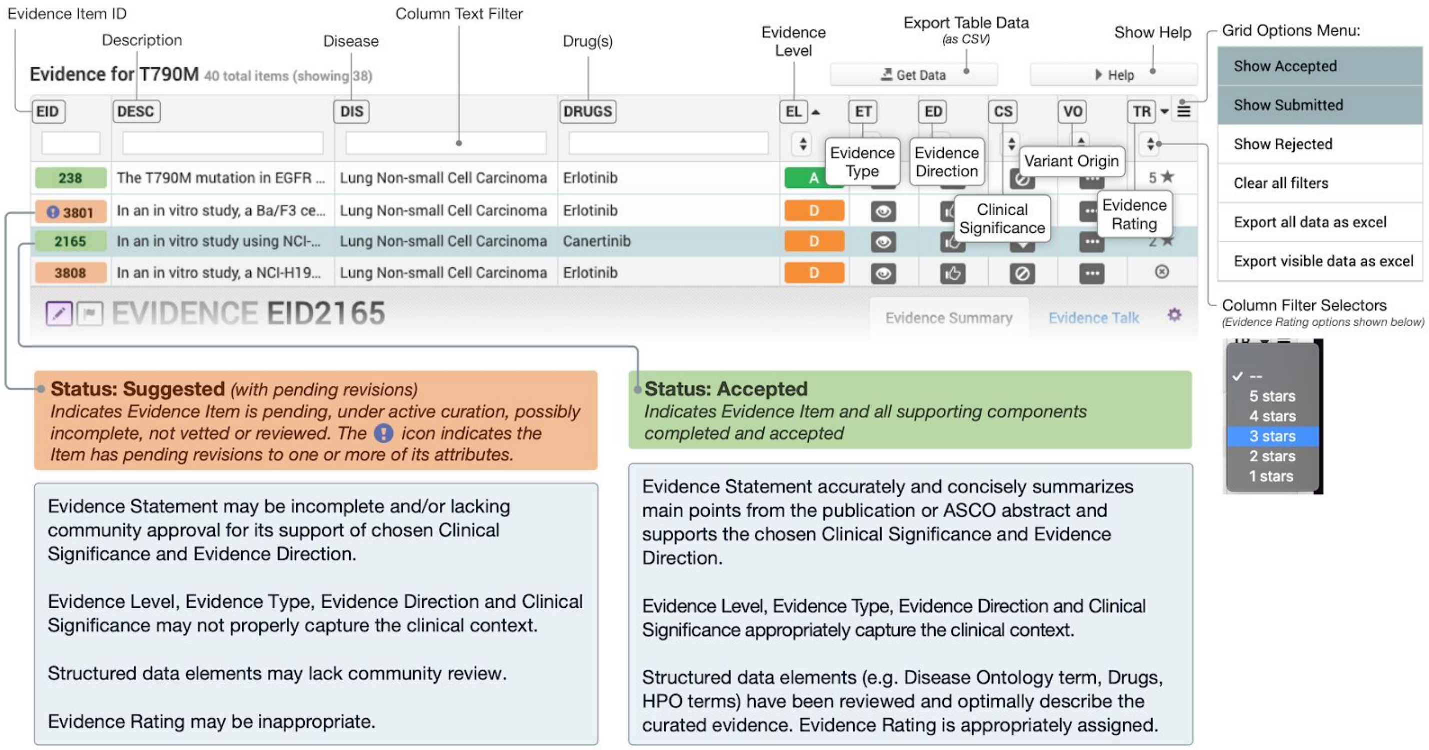

Curation Practices:

- Evidence Items should generally be prepared from primary literature rather than from review articles. It is recommended that CIViC Curators use reviews to identify primary literature referenced in the review and curate individual Evidence Items based on the review cited articles. Reference articles can also be used to develop Gene and Variant summaries.
- When curating new evidence, the Curator should keep in mind the existing evidence for that Variant and Evidence Type.
    - For clinical trials and case reports (Levels A, B, and C), overlapping patient populations should be avoided, if possible, or carefully noted to alert users of this nuance and avoid conclusions that mistake these studies as independent.
    - Disease stage, prior treatments, and other experimental details influencing evidence interpretation should be captured within an Evidence Item to maximize user comprehension of the underlying study and the appropriate context in which it is relevant. Such details are critical parts of clinical guidelines and can impact which clinical guidelines should be used, and can impact drug sensitivity annotation as well (see EID1008 and EID1009 which have different Clinical Significance but same drug and disease).

Examples of Variant Origin

Fig. S16. Example of Evidence Item where the Variant Origin is not applicable (N/A)

Below is an Evidence Item that supports sensitivity/response to Trastuzumab for a patient with breast cancer and an *ERBB2* - Amplification variant. In this example, the clinical trial describing this Evidence Item refers to patients with either Amplification or Overexpression of ERBB2. In the case of over-expression (measured by IHC) or Amplification (measured by FISH), typically only tumor samples are assayed. Thus it is not possible to definitely state that the variant is of somatic origin. Furthermore, in some cases over-expression may be driven by epigenetic causes where a variant does not apply. For these reasons, the Variant Origin has been entered as N/A.

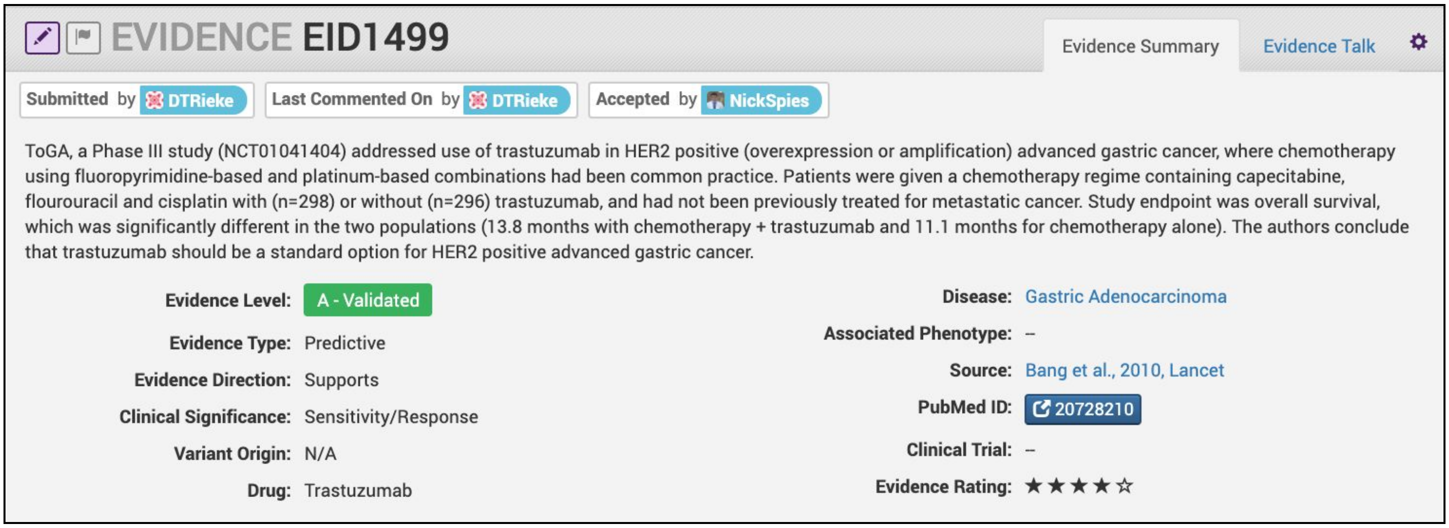

Curation Practices:

- N/A Variant Origin is particularly used in cases that involve differences in expression, methylation, or other post-translational modifications.
- Variants characterized in Functional Evidence Items with *in vitro* data will usually have N/A Variant Origin.
- Variant Origin is chosen from the following options:
  - Somatic
  - Rare Germline
  - Common Germline
  - Unknown
  - N/A
- Common Germline variants, recurrent in human populations, are generally not associated with concrete clinical actions or interpretations, but may be entered if there is relevant clinical evidence (e.g. pharmacogenetic variants that predict adverse drug responses or variable sensitivity to cancer drugs).

Examples of Evidence Levels

Fig. S17. Example of a A-Level (Validated) Evidence Item

Below is an example of an A-level (Validated) Evidence Item for the *BRAF* - V600E Variant. In this example, the Evidence Item is describing the Phase 3 randomized clinical trial that was submitted to the FDA for therapeutic approval of Vemurafenib with Dacarbazine for treatment of untreated, metastatic melanoma.

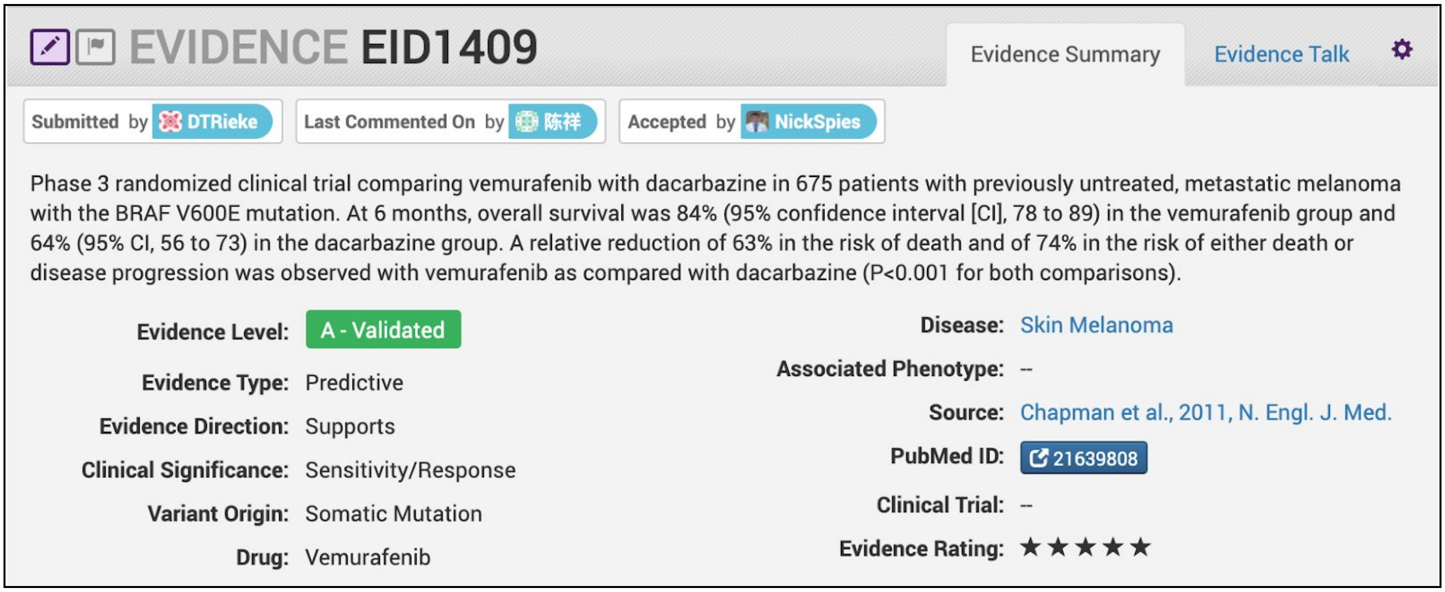

Curation Practices:

- Typically, A-level Validated Evidence Items describe Phase III Clinical Trials (for therapeutics or companion diagnostics), which are subsequently submitted to the FDA for pre-market approval.
- In general, Evidence Items derived from any study cited in approvals, established practice guidelines, or considered the definitive practice-changing study, may be labeled Level A. In some cases Phase I trials can meet this requirement (see EID1187).
- Evidence Statements should include the gene/variant being evaluated, the study population, disease state, study size, statistical significance (e.g., p-value, confidence interval), duration of the study, and other relevant information that is required to assess the evidence for variant interpretation.
- Evidence Items derived from publications describing practice guidelines (e.g. WHO diagnostic criteria) are labeled A-Validated Evidence Level.

Fig. S18. Example of a B-Level (Clinical) Evidence Item

Below is an example of a B-level (Clinical) Evidence Item for the *BRAF* - V600E Variant. In this example, the Evidence Item is describing a Phase 2 randomized clinical trial that was used to assess preliminary efficacy of the use of Vemurafenib for treatment of patients with previously treated skin melanoma.

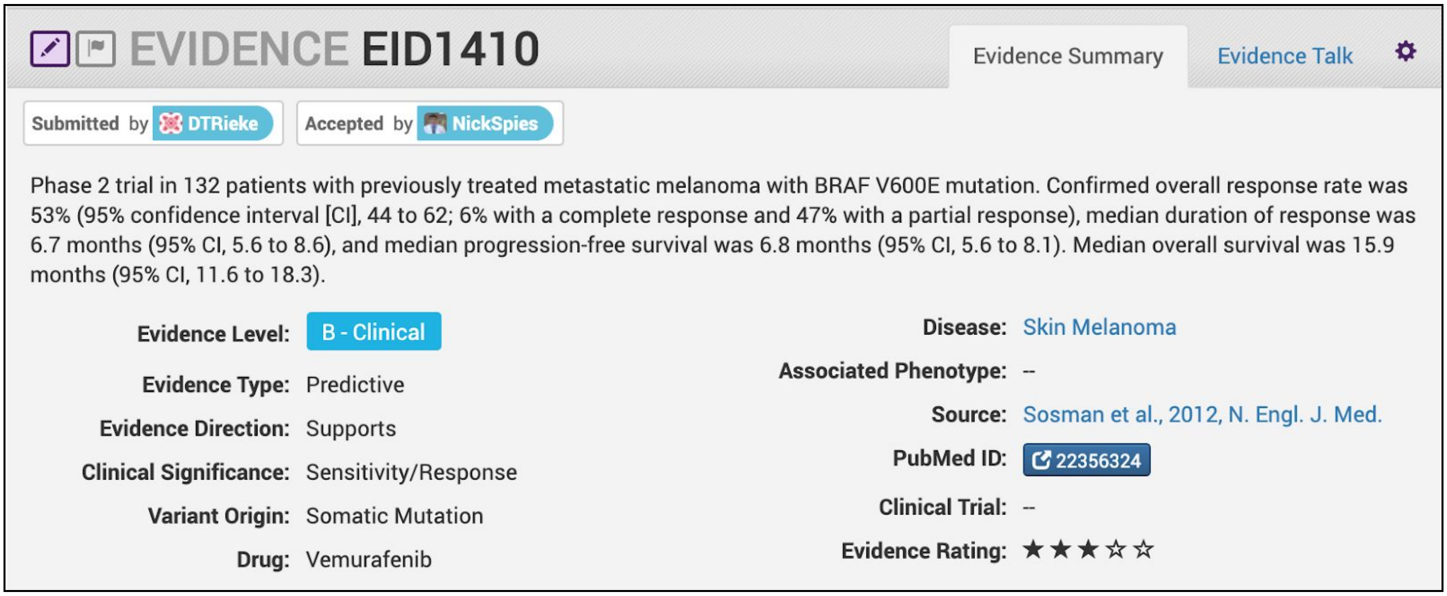

Curation Practices:

- B-Level Evidence Items can describe trials submitted to the FDA during the approval process; however, relative to A-Level Evidence Items, B-Level Evidence Items typically have a smaller sample size or assess less significant outcomes (e.g., response rate instead of overall survival).
- Phase I, II, and III clinical trials make up a significant percentage of Level B Evidence Items.
- For curation of Phase I evidence, notes on treatment related adverse events may be added to the main evidence statement describing the variant-positive patient subgroup response to treatment, as dosing and adverse events are among the main focuses of Phase I studies.
- B-Level Evidence Items do not have to be derived from clinical trials but also can describe studies which attain a sufficient sample size to be considered more informative than a series of case studies, and ideally have some component of statistical conclusions in their results.
- Greater than five patients is typically a minimum requirement for an Evidence Item to be considered B-Level, although B-Level rating is generally not based on patient number alone, but also on the consistency of conclusions across the patients studied, ideally with a statistically significant result.
- B-Level Evidence Statements should include the gene/variant being evaluated, the study population, disease state, study size, statistical significance (e.g., p-value, confidence interval), duration of the study, and other relevant information that is required to assess the evidence for variant interpretation.
- Categorical CIViC Variants (sometimes called bucket variants colloquially) often appear in B-Level Evidence Items describing clinical trials, which pool together patient populations with mutations of a certain class (e.g. “EGFR mutation”, See **Fig. S22**), in order to attain a disease specific, statistically significant, clinical results across the patient population (e.g. Trastuzumab resistance in HER2 positive breast cancer).

Fig. S19. Example of a C-Level (Case Study) Evidence Item

Below is an example of a C-level (Case Study) Evidence Item for the *BRAF* - V600E Variant. In this example, the Evidence Item is describing a single patient with the *BRAF* - V600E Variant who demonstrated sensitivity/response to Pictilisib in the disease context of melanoma. This Evidence Item was classified as a Case Study because it described results for a single patient with advanced melanoma who had been enrolled in a larger Phase I clinical trial that evaluated 60 patients with advanced solid tumors and any *BRAF* variant for sensitivity to Pictilisib.

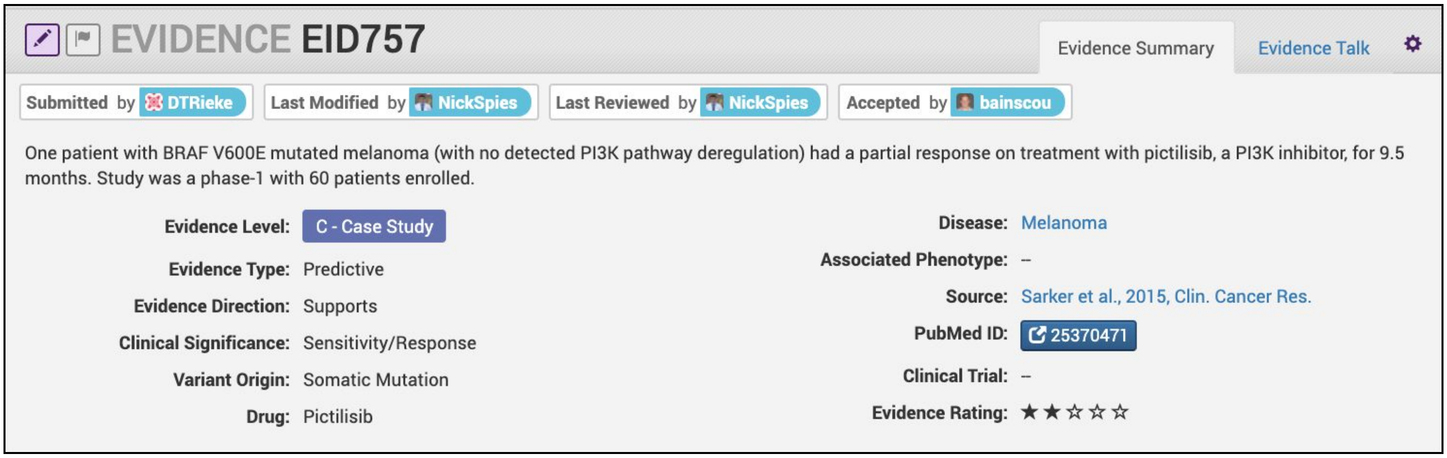

Curation Practices:

- C-level Evidence Items (EIDs) should describe a specific CIViC Variant and likely will not apply to a categorical variant (**Fig. S8**).
- In some cases a clinical trial employing a categorical variant (e.g. EGFR mutation) will contain additional supplementary information on individual patient mutations and outcomes (e.g. CR, PR, SD or PD as best response). In such cases, along with the B-level Evidence Item based on the categorical variant, individual C-level case study Evidence Items can be curated for each listed variant. (See **Fig. S22**)
- Evidence Items involving fewer than around five patients, and in the absence of control patients without the variant, are typically considered to be C-level Evidence Items.
- Evidence Statements should include the gene/variant being evaluated, the study population, disease state, study size, statistical significance (e.g., p-value, confidence interval, if applicable), duration of the study, and other relevant information that is required to assess the evidence for CIViC Variant annotation.
- Case study EIDs may receive a higher Evidence Rating when more evidence associating the variant with the clinical annotation is presented. For instance, a Case Study EID which supports variant A causing resistance to drug X may receive a higher Evidence (Star) Rating when the report describes a previously sensitive patient who had sequencing prior to and after resistance to drug X was observed, with variant A only appearing at appreciable levels in the post resistance tumor sequencing data. In contrast, a similar report where sequencing is only performed after resistance may receive a lower Evidence Rating.

Fig. S20. Example of a D-Level (Preclinical) Evidence Item

Below is an example of a D-level (Preclinical) Evidence Item for the *BRAF* - V600E Variant. In this example, 49 BRAF-mutant melanoma cell lines exhibited resistance to a combination of dactolisib and selumetinib treatment. Note that older drug names were used in this study, BEZ238 and AZD6244, but since then, the drug names have been updated to dactolisib and selumetinib. To reduce confusion, the more current names are used in the drug field and the Curator has included both the old and new names in the Evidence Statement.

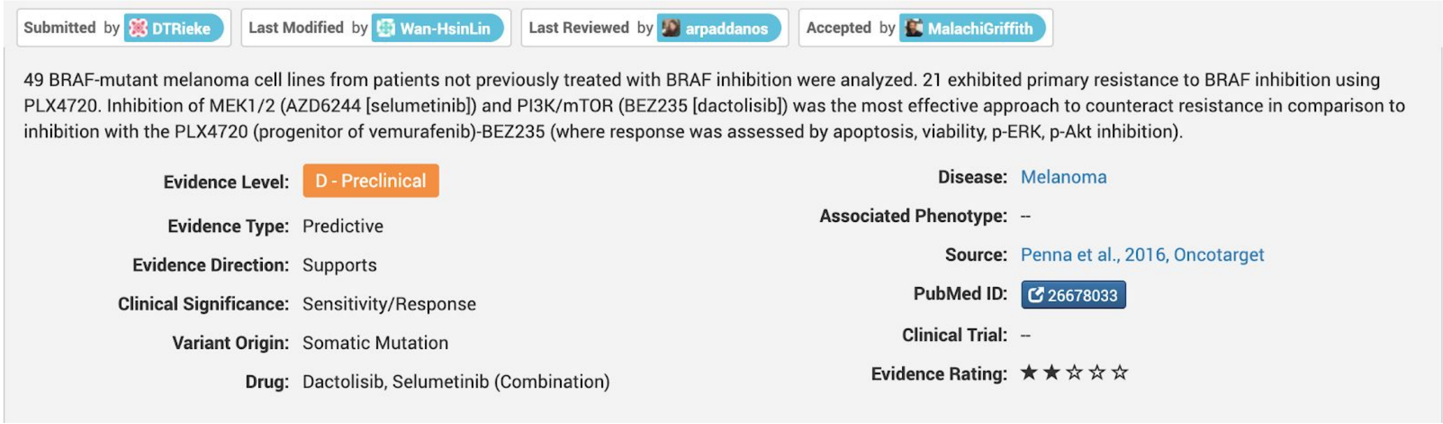

Submitted by DTRieke | Last Modified by Wan-HsinLin | Last Reviewed by arpaddanos | Accepted by MalachiGriffith

49 BRAF-mutant melanoma cell lines from patients not previously treated with BRAF inhibition were analyzed. 21 exhibited primary resistance to BRAF inhibition using PLX4720. Inhibition of MEK1/2 (AZD6244 [selumetinib]) and PI3K/mTOR (BEZ235 [dactolisib]) was the most effective approach to counteract resistance in comparison to inhibition with the PLX4720 (progenitor of vemurafenib)-BEZ235 (where response was assessed by apoptosis, viability, p-ERK, p-Akt inhibition).

Evidence Level: D - Preclinical
Evidence Type: Predictive
Evidence Direction: Supports
Clinical Significance: Sensitivity/Response
Variant Origin: Somatic Mutation
Drug: Dactolisib, Selumetinib (Combination)

Disease: Melanoma
Associated Phenotype: --
Source: Penna et al., 2016, Oncotarget
PubMed ID: 26678033
Clinical Trial: --
Evidence Rating: ★★☆☆☆

Curation Practices:

- D-level Evidence Items typically describe animal models or cell line studies. The sample size for these studies can influence the Evidence Rating, whereby increased numbers of mice or independent biological replicates used should increase the Evidence Rating.
- A concise description of the experiments performed should be prepared by the Curator, supporting the Evidence Item Clinical Significance, and describing the controls that were used, and the significant findings that were observed.
- Evidence Statements should include the gene/variant being evaluated, the study population, disease state, study size, statistical significance (e.g., p-value, confidence interval), duration of the study, and other relevant information that is required to assess the evidence for CIViC Variant annotation.
- When choosing a disease for Preclinical Evidence Items, it should reflect the context of the preclinical experiments which were performed. .
    - In some cases the preclinical work described in a study may apply broadly, beyond the specific cell line used, such as EID1356, using Ba/F3 cells where the selected Disease is "Cancer" (Disease Ontology ID 162).
    - Preclinical studies done using cells derived from patients with a specific disease will generate EIDs with the Disease and DOID specific to the patients.

Fig. S21. Example of an E-Level (Inferential) Evidence Item

Below is an example of an E-level (Inferential) Evidence Item for the *BRAF* - V600 Amplification Variant. In this example, the Evidence Item is describing how *BRAF* - V600E Amplification could be a mechanism of selumetinib resistance in patients with colorectal cancer.

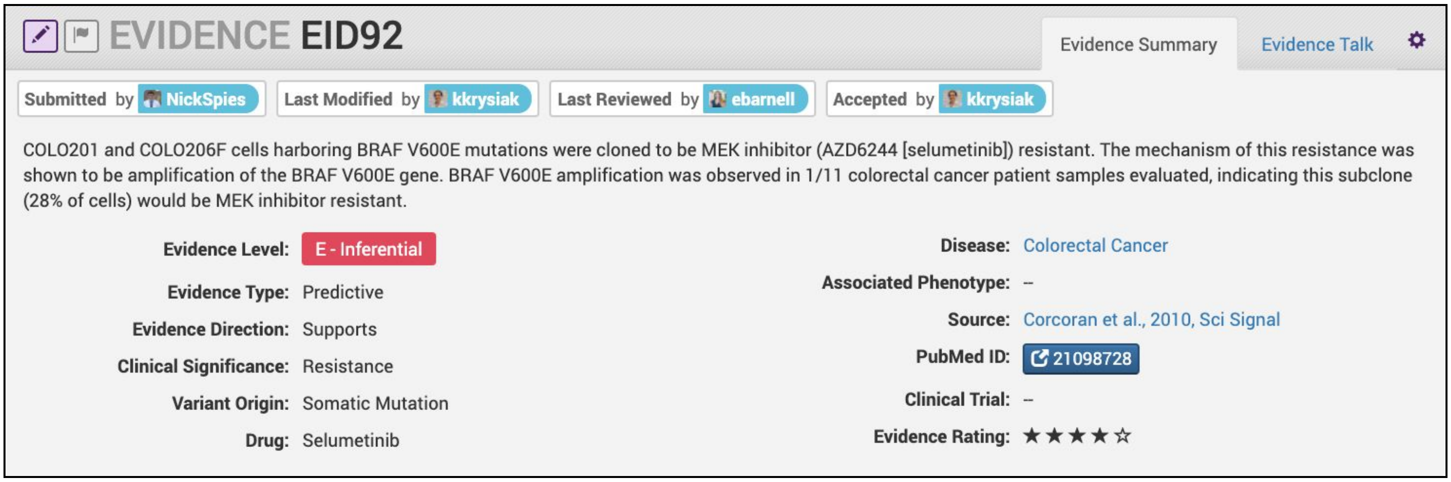

Curation Practices:

- E-Level Evidence Items provide inferential annotation for the associated variant. This could mean that the variant in question was not directly observed, or that the results from the study do not directly evaluate the claims made by the Evidence Item.
- E-Level Evidence Items can be derived from *in silico* predictions, cell lines, animal models, or human studies.
- Evidence Statements should include the gene/variant being evaluated, the study population, disease state, study size, statistical significance (e.g., p-value, confidence interval), duration of the study, and other relevant information that is required to assess the evidence for CIViC Variant annotation. Often these data are not available for E-Level Evidence Items.

Fig. S22. Level B and C Evidence Items from clinical trial data

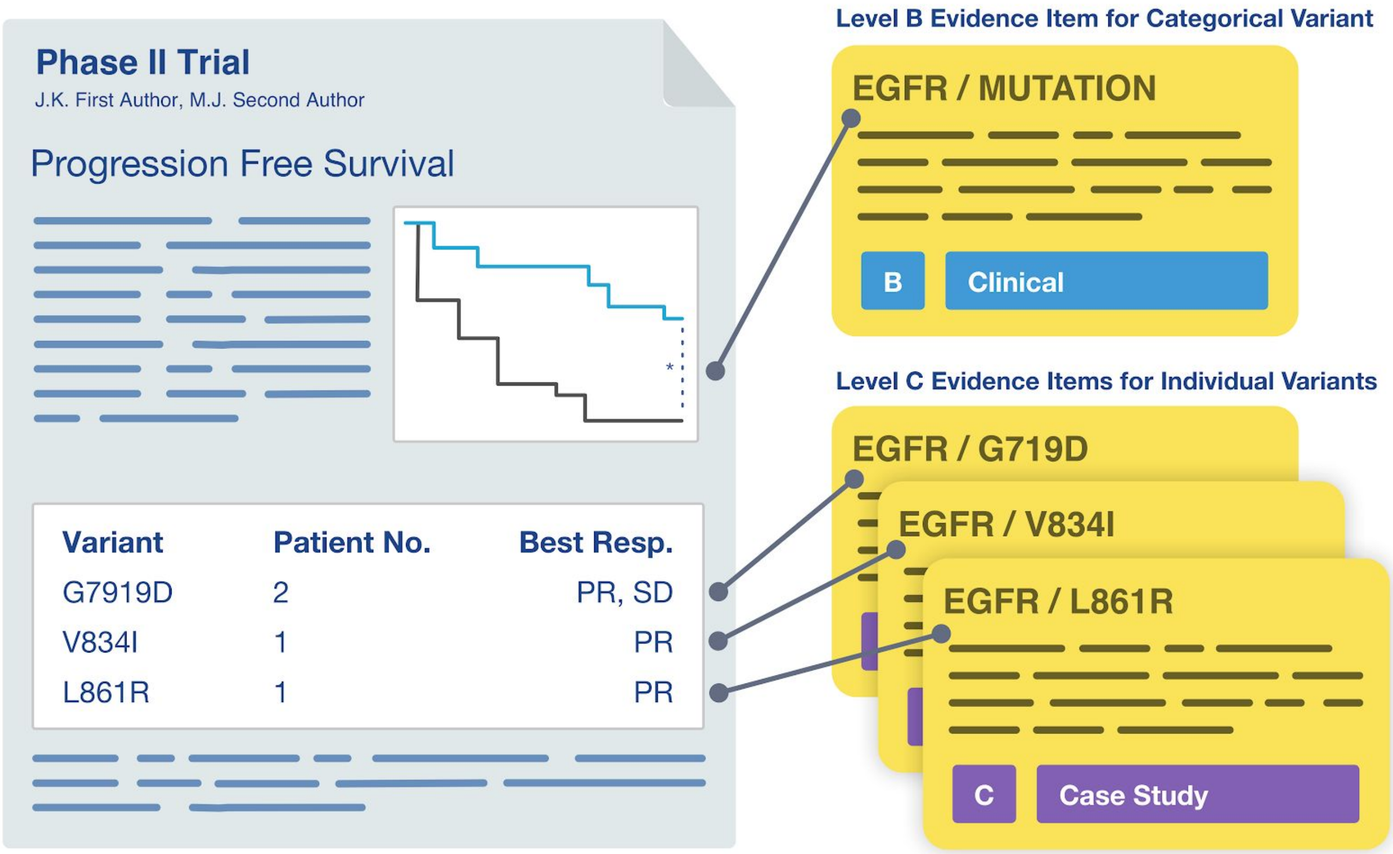

When curating evidence obtained from clinical trials on groups of patients where data is pooled into categorical variants (e.g. EGFR MUTATION, See **Fig. S8**), Level B clinical results may be obtained, which for example could report a statistically significant difference on a clinically relevant parameter such as partial response (PR) between pooled wildtype vs. mutant patients. Sometimes the trial will also report individual patient parameters such as variant, age, sex, and individual outcomes such best response, overall survival, etc are also reported. In these cases, the study data may be used to create multiple CIViC Evidence Items (EIDs) as described below. Note this figure is loosely based on an EID set in CIViC obtained from PMID:21531810, which can be seen in CIViC on the Evidence Source page https://civicdb.org/sources/1503/summary.

- Statistical results may be obtained from the study to annotate a Categorical CIViC Variant (sometimes called bucket variant), which pools together a category of sequence variants (for example EGFR MUTATION). Significantly longer progression free survival (PFS) may be observed in the mutant group (grouped under the Categorical CIViC Variant) vs. the wildtype group, when given a certain drug. In this case, this result may be reported as a CIViC Level B Evidence Item under the CIViC Categorical Variant EGFR MUTATION, with Evidence Direction and Clinical Significance “Supports Sensitivity/Response” to the drug used.
- When a sufficient level of individual patient detail is present, including the individual patient variants along with an important clinical parameter such as their best response, then this data set can be used to generate a set of CIViC Level C Evidence Items for the patients, each one associated with the respective CIViC Variant that was observed in the individual patient, along with the outcome. Note that even if the entire group showed statistically significant improvement with the Categorial Variant, this does not mean every patient did better, e.g. if a patient with variant X123Y had progressive disease as best response, then this would result in a Level C EID with Evidence Direction and Clinical Significance of “Does not support Sensitivity” for the CIViC Variant X123Y.

Examples of Evidence Types

Fig. S23. Example of a Predictive Evidence Type

Below is an example of an Evidence Item that illustrates the Predictive Evidence Type. This example describes the CLEOPATRA trial (NCT00567190), which evaluated 808 patients with HER2-positive metastatic breast cancer. These patients demonstrated significant sensitivity/response when treated with combination therapy of docetaxel, pertuzumab and trastuzumab.

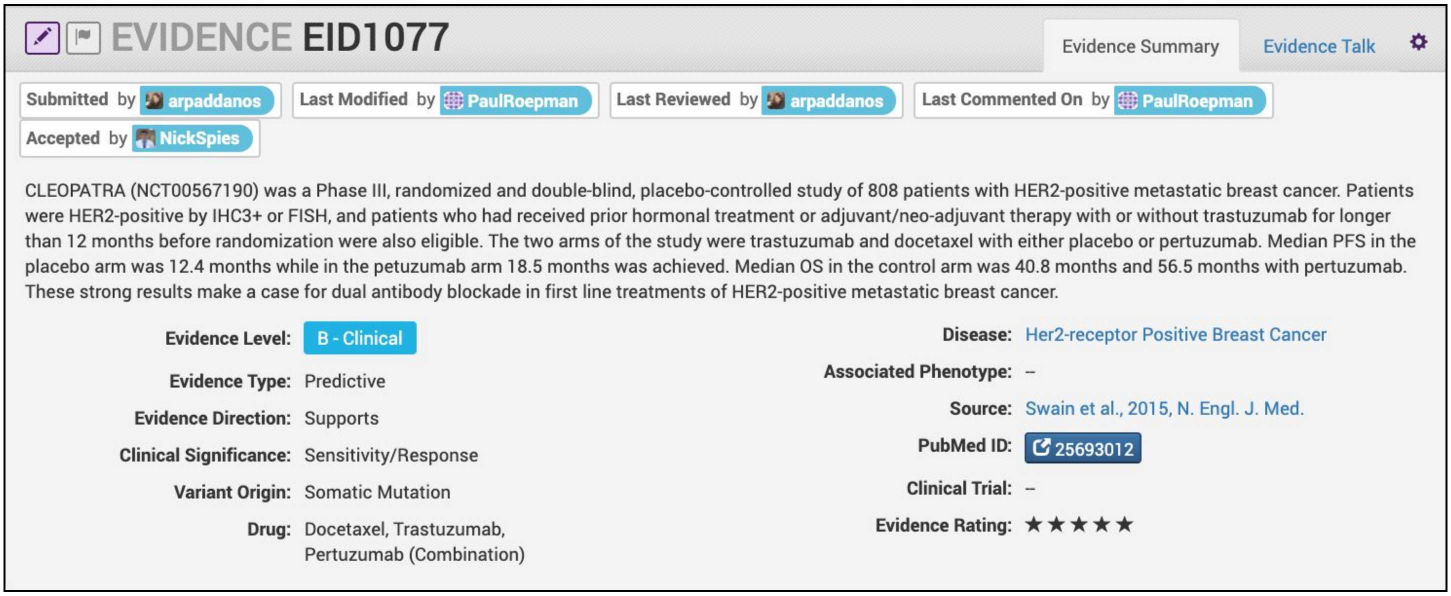

Curation Practices:

- Predictive Evidence Items should include the NCI Thesaurus Drug Name(s) when available (See **Fig. S34**) and Drug Interaction Type (for multiple drugs).
- The most current name of the Drug (excluding trade names) should be used in the Drug field to reduce duplication. The Evidence Statement should contain the drug name used in the study with the current name in brackets, when applicable (see **Fig. S20** for an example).
- Drug Interaction Types are required anytime more than one drug is mentioned for a given study. If multiple drug interaction types are at play (e.g., combinations and substitutes), Curators should consider separating these concepts into more than one Evidence Item.
- If applicable, the Clinical Trial name and ID should be included in the Evidence Statement. Any clinical trial IDs available in PubMed for the Source linked to this Evidence Item will be automatically imported and linked to this Evidence Item when the PubMed Source is imported into CIViC.
- The duration of exposure to the drug and confounding interactions (e.g., wash-out periods, previous treatment, cancer stage) should be listed.
- Assigning a Clinical Significance of Sensitivity/Response can depend on factors such as response rate, which will vary significantly with disease and treatment. In some cases a response rate of 15% may represent a significant improvement, and merit a valuation of Sensitivity/Response. A general guideline for CIViC curation is to follow the author's published (and peer-reviewed) interpretations and conclusions of the results.
- Extensive guidelines, use cases, and examples for curation of predictive evidence are given in **Fig. S28** and **Table S6**.

Fig. S24. Example of a Diagnostic Evidence Type

Below is an example of an Evidence Item that illustrates the Diagnostic Evidence Type. This example describes the World Health Organization guidelines for classifying chronic myelomonocytic leukemia (CMML). Specifically, if a patient has a PCM1-JAK2 fusion or a rearrangement involving PDGFRA, PDGFRB, or FGFR1, especially in the setting of eosinophilia, the patient does not have CMML.

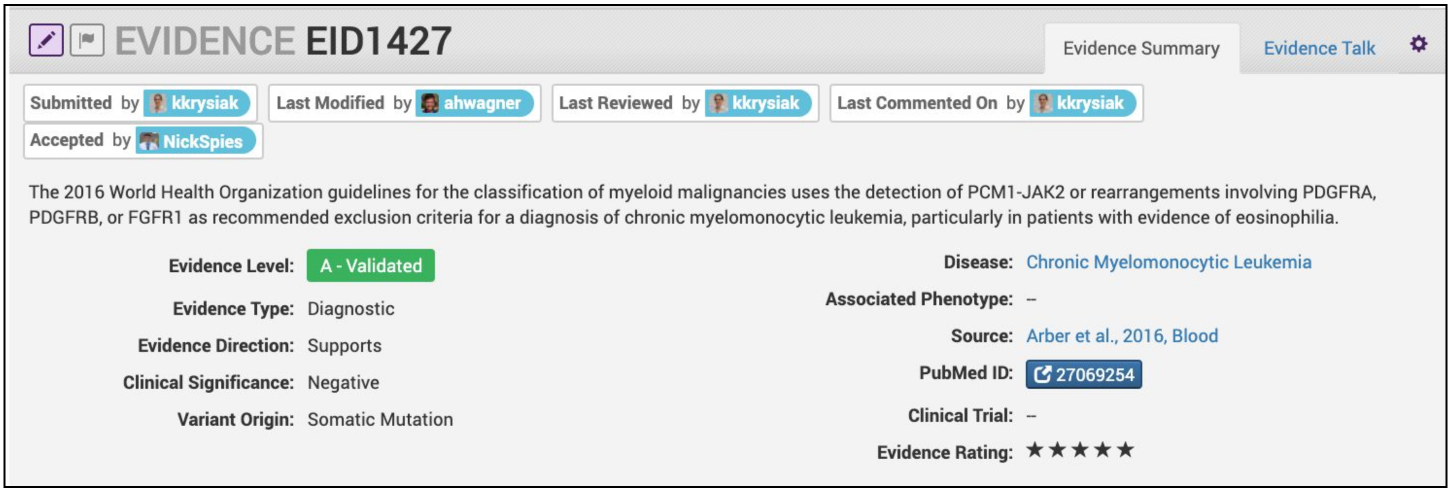

Curation Practices:

- Diagnostic Evidence Items (EIDs) should only be used if the variant assists in labeling the patient with a specific disease or disease subtype and should not be used to denote that the particular variant is prevalent in a specific disease.
- Generally, Diagnostic Evidence Items annotate CIViC Variants that can help accurately diagnose a cancer type or subtype with high sensitivity and specificity, for which diagnosis may otherwise be challenging.
- Diagnostic Evidence Items are very closely tied to the terms of the Disease Ontology (DO) in CIViC. The Disease Ontology works to actively generate mappings to other highly used ontologies, but the terms in the DO are generally accepted diseases which are part of medical practice. Therefore, literature proposing a novel disease type - for instance studies suggesting a novel cancer subtype defined by the presence of a specific oncogenic variant - are not generally admitted as part of the CIViC data model. Alternatively, if a CIViC Curator with expertise in the field feels that the novel subtype has met with a sufficient level of acceptance (or, if an accepted disease term is simply missing from the DO), the Curator may submit an Evidence Item with this novel disease, using the non-DO term (checking the "Could not find disease" box in the Add Evidence form, **Fig. S14**), and submit this new term to the Disease Ontology Term Tracker for addition of the new disease term (http://disease-ontology.org/faq/).
- Literature describing diagnostic practice guidelines (such as those of the World Health Organization) may be used in EID curation, and submitted as A-Level Evidence Items.
- Literature describing small numbers of observations in patient samples of a certain variant, where the authors state that the variant may have diagnostic value, may be admitted as lower star Case Study (C-level) data. Similar literature employing larger numbers could be labeled as Clinical (B-Level).
- Guidelines and use cases for curation of diagnostic evidence are given in **Table S6**.

Fig. S25. Example of a Prognostic Evidence Type

Below is an example of an Evidence Item that describes a Prognostic Evidence Type. This example describes a 406-patient trial whereby observation of any somatic *TP53* mutation in chronic lymphoblastic leukemia conferred poor prognosis relative to wildtype *TP53*.

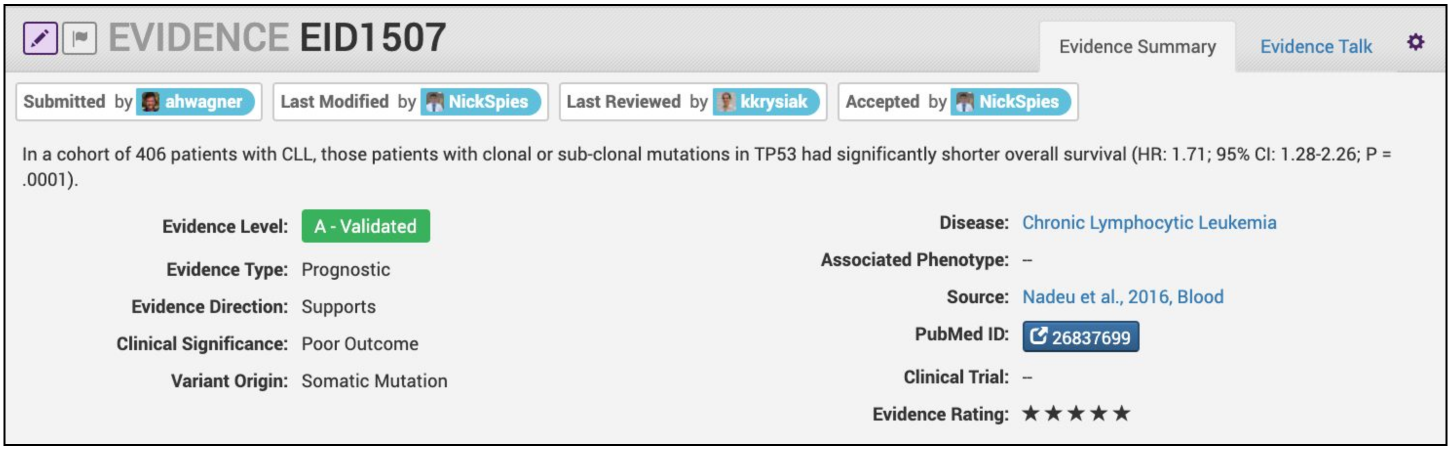

Curation Practices:

- Prognostic Evidence Items should include the measured outcome (e.g., overall survival, complete response, partial response), number of subjects and applicable statistics.
- If described in the literature, a definition of the measured outcome should be given.
- Prognostic evidence is characterized by either better outcomes for patient subpopulations with the given variant, which are not specific to any particular treatment context, or worse outcomes which are not indicative of variant resistance to a specific treatment. Instead, the change in outcome should be generally correlated to the presence of the variant, but independent of any specific treatment type.
- In some cases, a variant subpopulation with worse outcome may benefit from subsequent therapy targeted to that variant (e.g., HER2 amplification in breast cancer).
- Guidelines, use cases, and examples for curation of prognostic evidence are given in **Fig. S28** and **Table S6**.

Fig. S26. Example of a Predisposing Evidence Type

Below is an example of an Evidence Item that describes a Predisposing Evidence Type. This example describes a study where the *VHL* - R167Q (c.500G>A) Variant was described in a set of patients and evidence for the PP1 ACMG-AMP criteria was documented. Hemangioblastoma and pheochromocytoma were seen in patients and are reported as Associated Phenotypes, while the Disease is Von Hippel-Lindau Disease.

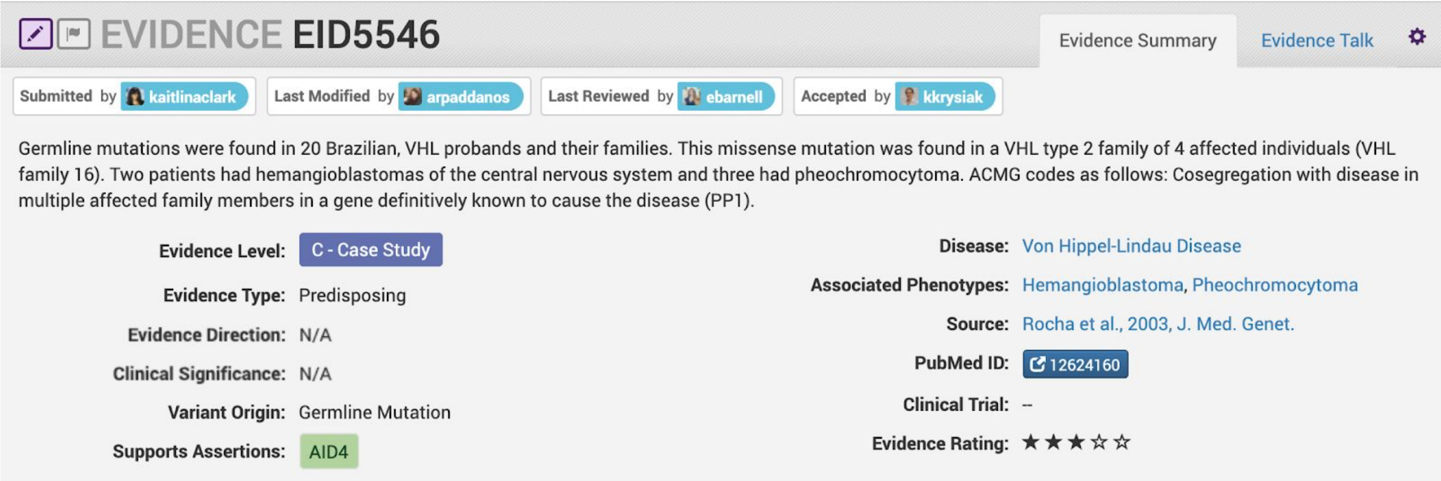

Curation Practices:

- Typically, but not always, Predisposing Evidence Items are written for rare or common germline variants. In rare circumstances, the patient can have a predisposing variant that develops as a result of a somatic mutation or mosaicism during embryogenesis that is widespread but not necessarily heritable.
- ACMG-AMP evidence codes (Richards et al. 2015) (ACMG criteria) are derived from the evidence presented in the specific Source and are listed at the end of the Evidence Statement with a brief justification for each code's use.
- ACMG evidence codes not directly derived from the Evidence Source associated with the Evidence Item (e.g. population databases for PM2) are captured at the Variant Summary (**Fig. S10**) or Assertion level (**Figures 4 and 5B**).
- The above Predisposing Evidence Item (EID) lists the ACMG code PP1 as derived from the literature source, which alone results in a ACMG-AMP classification of VUS. Therefore, this Evidence Item is combined with other *VHL* - R167Q (c.500G>A) Evidence Items for Von Hippel Lindau Disease, in order to create CIViC Assertions, where the ACMG codes from the different Evidence Items are combined and evaluated for pathogenicity (See **Figures 4**, **5B**, and **S41**). The EID depicted here is part of Assertion number 4 (AID4), where the Evidence Items combine to support a pathogenic annotation. Therefore Predisposing Evidence Items are not given Clinical Significance or Evidence Direction in isolation, and these fields are labeled **N/A**.
- In some instances, a publication will contain relevant germline variant evidence for curation into CIViC and EID creation, but that evidence will not be sufficient to fulfill any of the ACMG criteria (especially in some cases where the gene or disease-specific criteria may be more stringent). In this case Curators should indicate this at the end of the Evidence Statement, by adding a brief statement such as "No ACMG criteria met", in order to indicate to Editors and future Users that the evidence had been analyzed for the presence of ACMG codes during the curation process.
- If the Evidence Source provides incomplete but partial justification for some ACMG criteria, then those criteria should be listed at the end of the Evidence Statement, clearly labeled as only partially met, with a brief sentence justifying the inclusion of each ACMG code. Multiple partially met instances of an ACMG criteria from different EIDs supporting the same variant may then be assessed during creation of an Assertion for the variant to see if together they justify full support for the given ACMG code, leading to its inclusion in the Assertion.

Fig. S27. Example of a Functional Evidence Type

Below is an example of an Evidence Item that describes a Functional Evidence Type. This example summarizes the impact of a novel *KIAA1549-BRAF* fusion event on the function of the *BRAF* protein. Specifically, the fusion product showed gain of function activity in cell lines relative to wildtype kinase. This activity was also demonstrated to be comparable to a known gain of function variant, *BRAF* V600E.

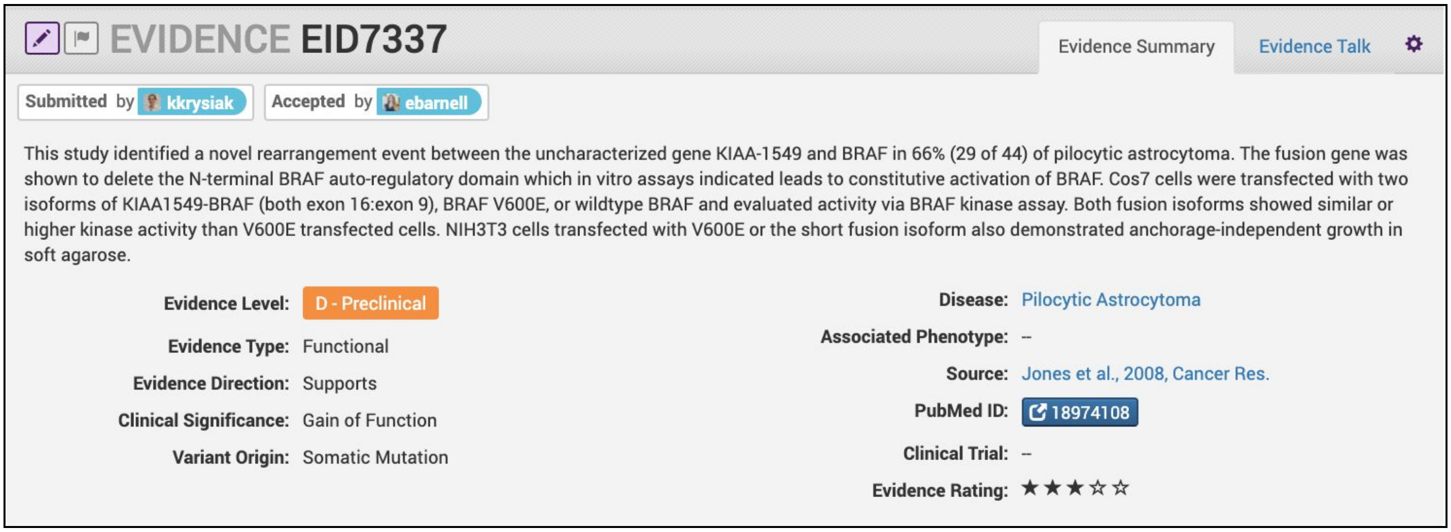

Curation Practices:

- Functional Evidence Items should describe how the variant alters biological function from the reference state. This can include a change in function or lack of change in function.
- Clinical Significance for Functional Evidence Types adhere to the following rules:
  - Gain of Function = A variant whereby enhanced function is conferred on the gene product;
  - Loss of Function = A variant whereby the gene product has diminished or abolished function;
  - Unaltered Function = A variant whereby the function of the gene product is unchanged;
  - Neomorphic = A variant whereby the function of the gene product is a new function relative to the wildtype function;
  - Dominant Negative = A variant whereby the gene product negates the function of a wildtype allele.
  - Unknown = A variant that cannot be precisely defined by gain-of-function, loss-of-function, neomorphic, dominant negative or unaltered function.
- Functional Evidence Items may be used to support certain ACMG codes (e.g. PM1). In these cases, the ACMG code should be listed in the Evidence Statement along with a brief justification for its inclusion.
- In some cases, Functional Evidence Items may appear as supporting evidence for a Predisposing Assertion, for instance in support of a PM1 evidence code.

Fig. S28. Interpreting clinical trial data to curate Predictive/Prognostic Evidence Items

This figure provides hypothetical examples for interpreting clinical trial data to curate Predictive and Prognostic Evidence Items. Each box represents a different hypothetical 2-armed clinical trial (Treatment vs. Control) where each arm contains wildtype (WT) and mutant (MT) patient populations. Each of the seven studies contains different values for progression free survival (PFS), which are assumed to represent significant differences between populations. The yellow boxes indicate recommended selections that can be made for the Evidence Type, Evidence Direction, and Clinical Significance. The text below these selections describes the study and data being evaluated.

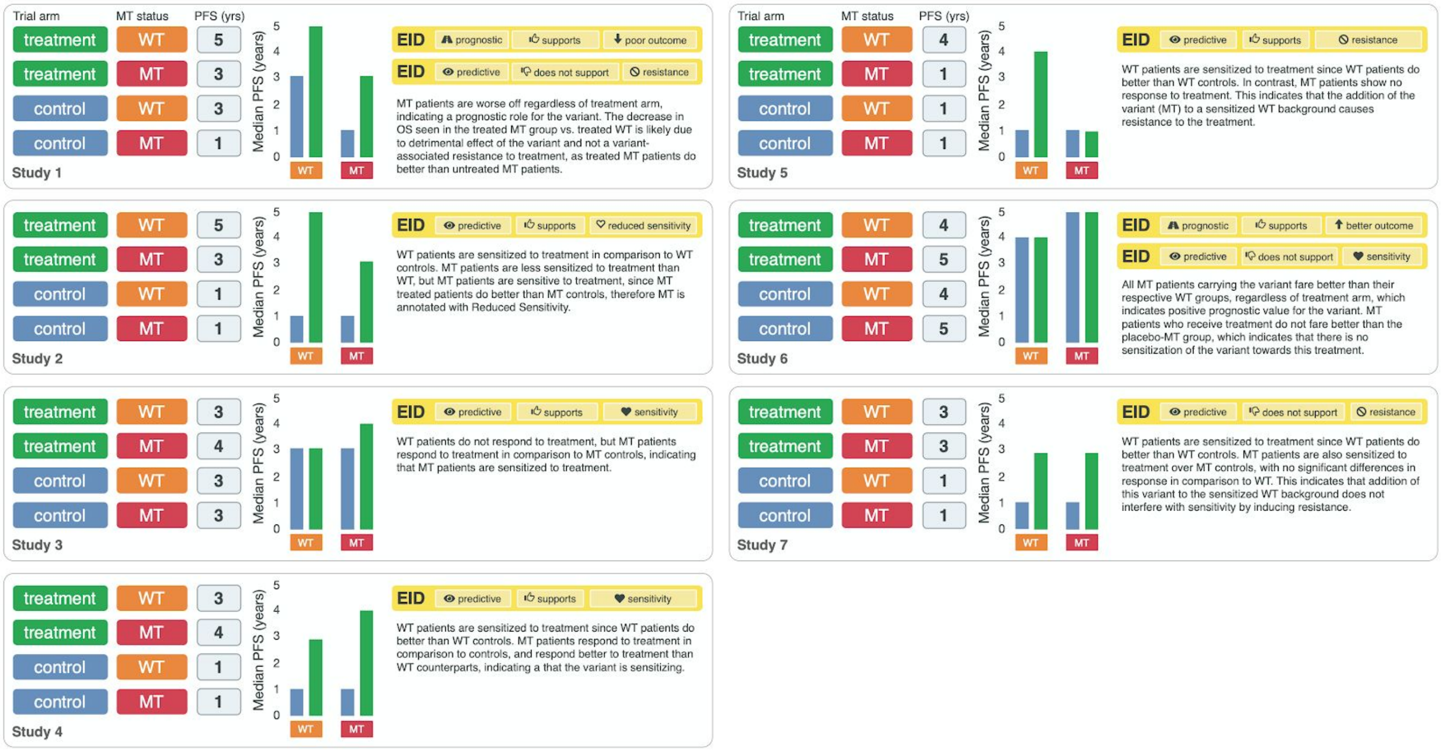

Curation Practices:

- These examples do not show all possible ways to obtain the listed Clinical Significances.
- It is recommended to always follow the authors' interpretations of the data and results when writing EIDs since these interpretations are peer reviewed when derived from PubMed ID, and are often a function of such disease specific parameters such as response rate.
- In these examples, studies with a Resistance annotation could be viewed as trials assessing secondary resistance mutations, in which case the WT group would be a sensitized background with a primary sensitizing mutation (e.g. EGFR L858R in NSCLC treated with TKI) and the MT group would be testing a secondary mutation for resistance to treatment (e.g. EGFR T790M).
- Note that in study 2 the Reduced Sensitivity annotation is used in the context of a mutation which is reducing drug response but not causing complete resistance, which could be for example a secondary mutation on a sensitized background. Another use case for the Reduced Sensitivity annotation is in comparison of a primary sensitizing mutation to an established sensitizing mutation for a given drug and disease type (described in **Table S6**). If this had been the case in this figure, then a better labeling of the Study 2 groups would be MT1 for WT, and MT2 for MT, since two different sensitizing mutations (Mutant1 and Mutant2) are being compared under the same disease and treatment.

Examples of Evidence Rating

Fig. S29. Evidence Item with 5-star Evidence Rating

The example Evidence Item below describes a Phase III clinical trial (PROFILE 1014) that evaluated the impact of the *ALK* - Fusions Variant on therapeutic response with crizotinib for patients with lung non-small cell carcinoma. The clinical trial was a randomized, double-blinded, placebo-control study of 343 patients that evaluated progression free survival, objective response rate, and quality of life. The results were published in the New England Journal of Medicine.

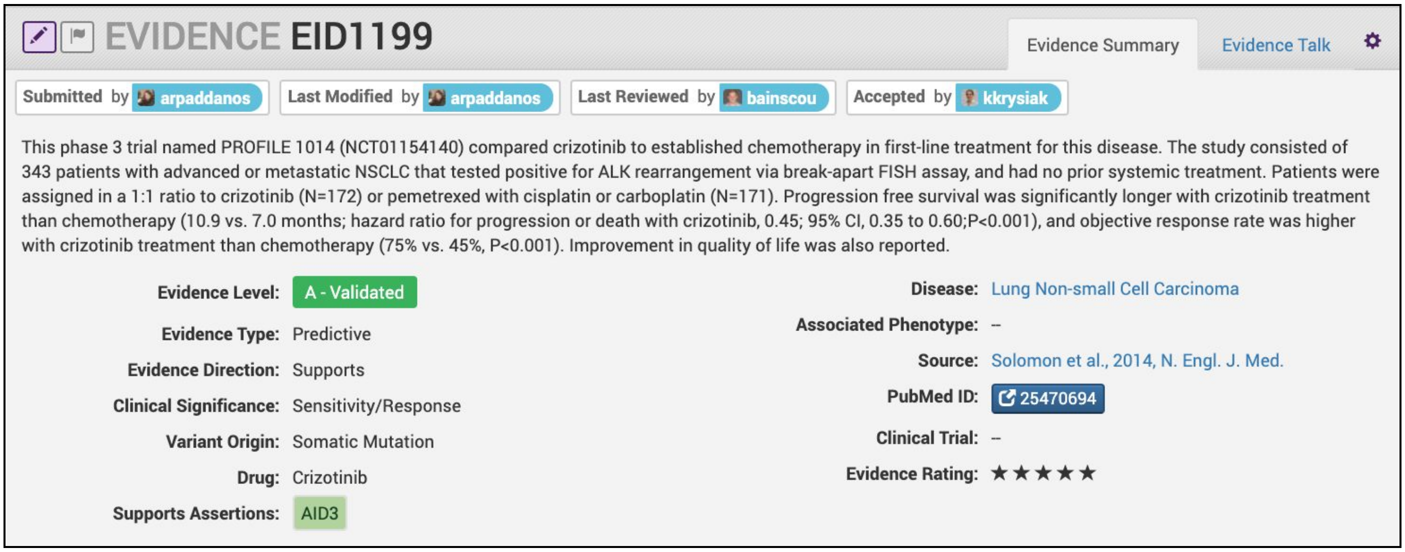

Curation Practices:

- Evidence Items with a 5-star Evidence Rating should be strong, well-supported evidence. Experiments should be well controlled, and results should be clean and reproducible across multiple replicates. Evidence should be confirmed using independent methods and the study should be statistically powered whenever possible.
- In general, Evidence Rating is a rating of the unit of evidence extracted from a data source (publication or ASCO abstract) and is not a rating of the publication or abstract itself. Thus, an isolated supplementary figure in a high quality and well-researched publication may yield a relevant piece of clinical information on a CIViC Variant of interest, and an Evidence Item (EID) could be prepared from this figure. Due to the limited nature of the data supporting this type of Evidence Item, it would receive a lower Evidence Rating. This is because this rating applies only to the evidence used to create this single EID, not to the publication as a whole.
- Different Evidence Ratings may cause evidence from a single source to be split into multiple Evidence Items (EIDs). For example, preclinical work might show a variant responding equally well to two different drugs in parallel sets of experiments, allowing a Curator to write a single EID describing both results, and using the two drugs as substitutes. Alternately, if both drugs demonstrate a similar result, but the experiments show one responds appreciably better than the other, then two EIDs should be written, one for each drug, and with different Evidence Ratings.

Fig. S30. Evidence Item with 4-star Evidence Rating

The example Evidence Item below describes a Phase 2A clinical trial with multiple arms that included evaluation of the therapeutic effect of pertuzumab and trastuzumab on 37 patients with *HER2* - Amplified colorectal cancer. The study was sufficiently powered to demonstrate an increase in patient response and remission provided their advanced, refractory state and relatively rare molecular alteration for that tumor type. The study was part of a clinical trial that was registered through the NIH and was published in the Journal of Clinical Oncology.

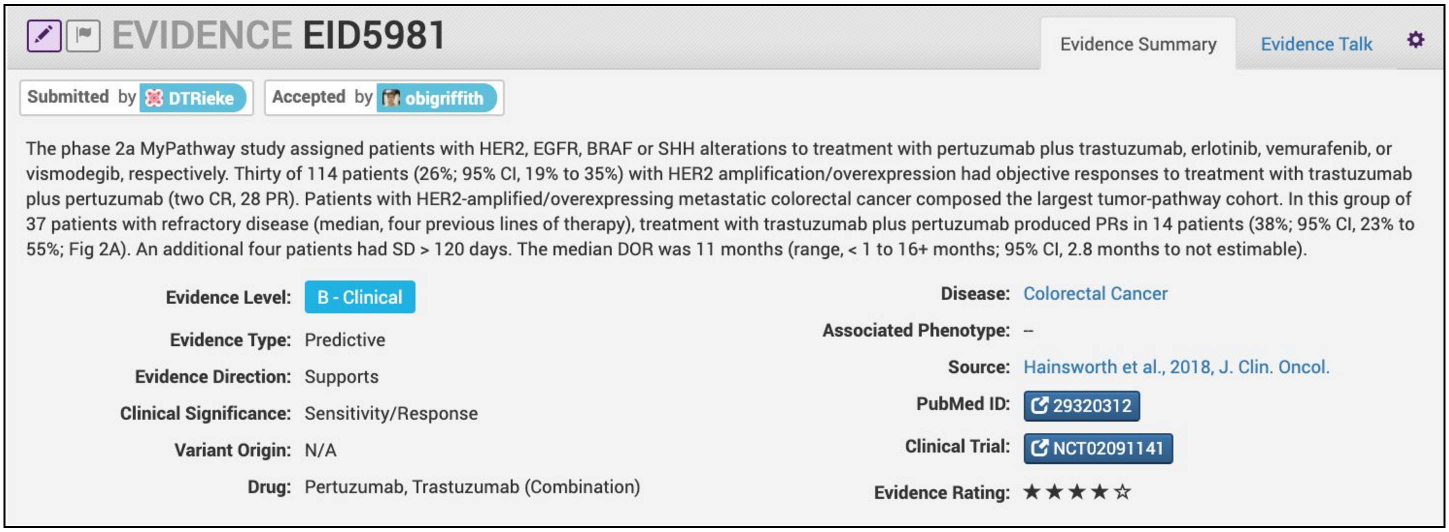

Curation Practices:

- Evidence items with a 4-star rating should be strong, have well-supported evidence, well-controlled experiments, and convincing results. Any discrepancies from expected results are well-explained and not concerning.
- This example was similar in design as the 5-start example, however, the reduced sample size contributed to the reduction in the start rating.

Fig. S31. Evidence Item with 3-star Evidence Rating

The example Evidence Item below describes the same Phase 2A clinical trial from **Fig. S30**, but differs in the advanced solid tumor type being evaluated. In the subset of patients with bladder cancer, three of nine patients showed response to combination therapy with trastuzumab and pertuzumab, which supports sensitivity/response. Although this evidence item is derived from the same clinical trial, the reduction in Evidence Rating is representative of the smaller number of patients and large 95% confidence interval.

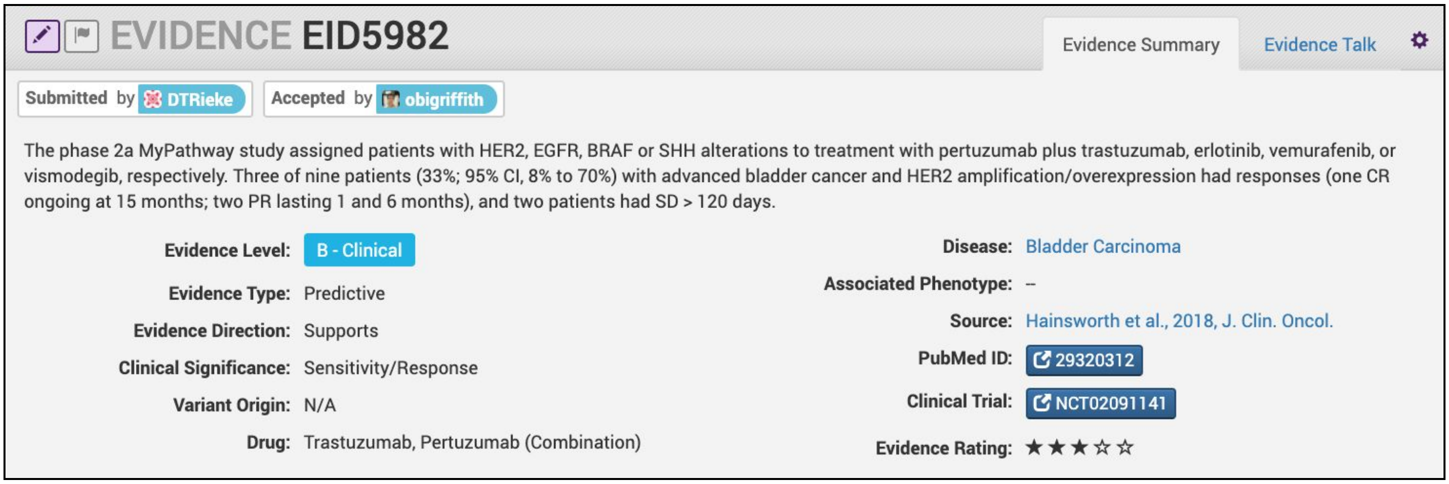

Curation Practices:

- Evidence Items with a 3-star rating are convincing but not supported by a breadth of experiments. These Evidence Items be smaller scale projects, or novel results without many follow-up experiments.
- Even though these Evidence Items might contain reduced amount of data discrepancies from expected results should still be explained and not concerning.

Fig. S32. Evidence Item with 2-star Evidence Rating

The example Evidence Item below describes a Phase II clinical trial that evaluated 29 patients with breast cancer who were being treated with either afatinib, lapatinib, or trastuzumab. In this study, 18 patients showed response to one of the therapeutics. The Evidence Rating for this Evidence Item was only 2 stars due to lack of evidence supporting the clinical claim (supports sensitivity/response). Specifically, the sample size was low for each of the three arms, there was no reported statistical significance. Additionally, the clinical endpoint for the study was objective response rate, which is not as strong of an endpoint as other metrics such as overall survival.

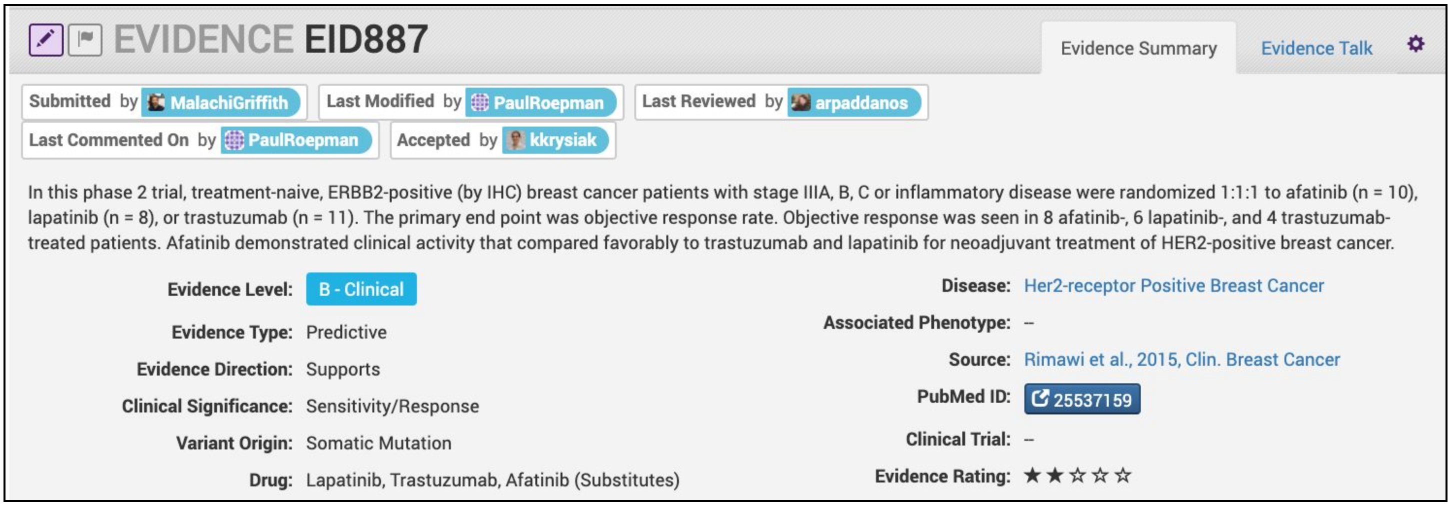

Curation Practices:

- Evidence items with a 2-star rating are not well supported by experimental data, and little follow-up data is available.
- Typically, Evidence Items received a 2-star rating if the experiments lack proper controls, have small sample size, or are not statistically convincing.

Fig. S33. Evidence Item with 1-star Evidence Rating

The example Evidence Item below describes a B-Level clinical study that evaluated 6 patients with *ERBB2* - Amplification for response to capecitabine, oxaliplatin, and chemoradiotherapy, with or without cetuximab. There was no difference in outcome between the 6 patients with the variant when compared to the 135 patients with no visible *ERBB2* - Amplification on FISH / IHC. The Evidence Item a heterogenous combination of variant detection methods, a low number of patients in the experimental arm (n=6) and overall low statistical power. Therefore, despite being a B-level Evidence Item, the Curator assigned the EID a 1-star Evidence Rating.

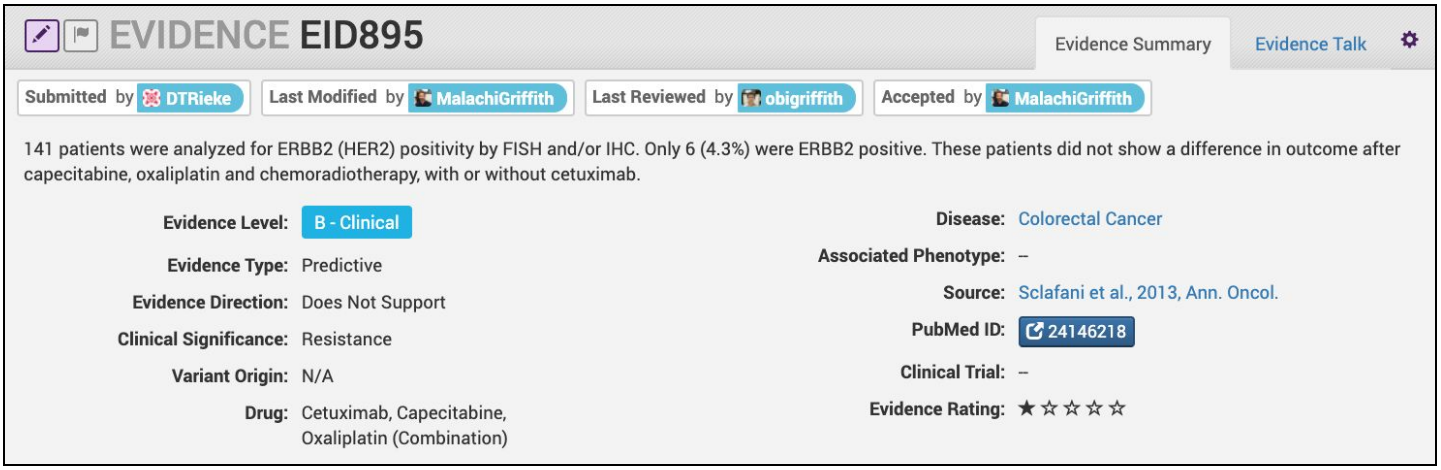

Curation Practices:

- Evidence items with a 1-star rating contain claims that are not well-supported by experimental evidence. Typically, the results are not reproducible and/or have very small sample size. No follow-up is done to validate novel claims.
- Typically, Evidence Items received a 1-star rating if the experiments lack proper controls, have small sample size, or are not statistically convincing.

Fig. S34 CIViC Drug Names curation

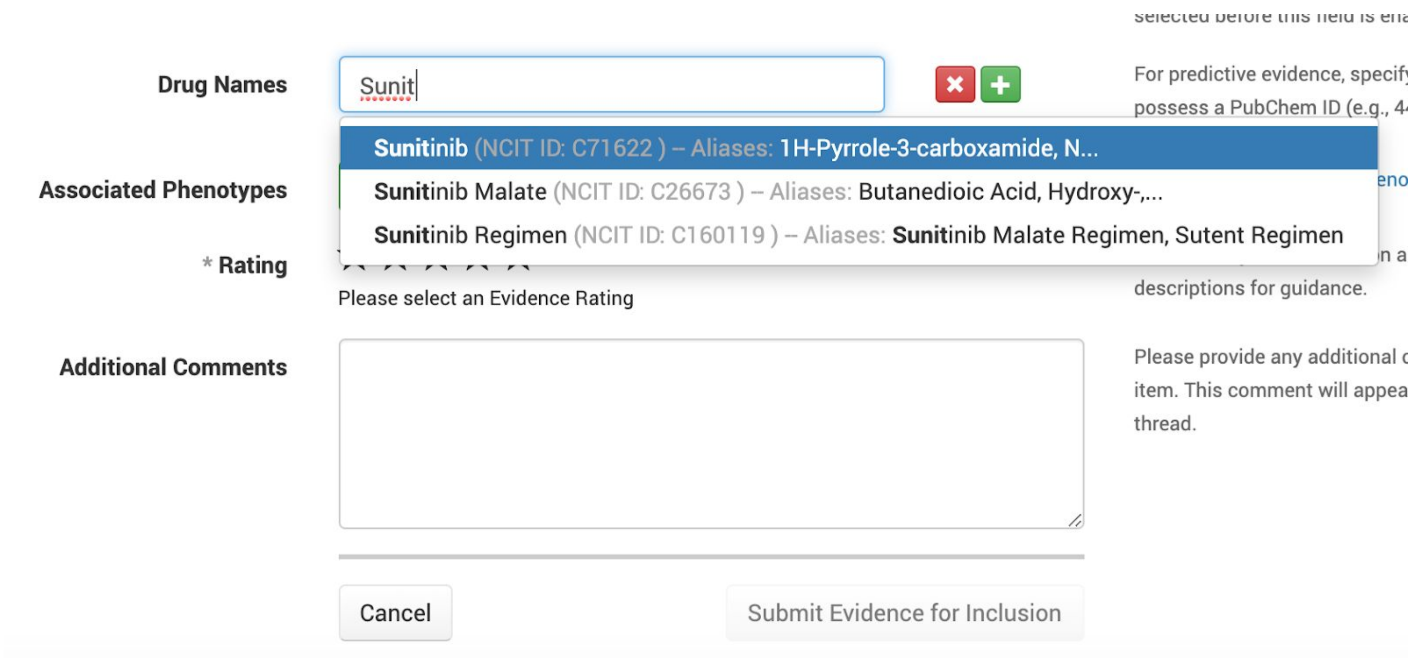

During curation of Predictive Evidence Items (EIDs), the curator is required to enter a treatment into the Drug Name field. CIViC Drug Names are drawn from the The NCI Thesaurus (https://ncit.nci.nih.gov/) whenever available. For drugs or treatments that do not have an NCIT ID, the curator is given the option to enter a new Drug Name into CIViC. When this occurs, it is requested that the curator also submit this new term to the NCIT (https://ncitermform.nci.nih.gov/ncitermform/).

- For the large majority of Evidence Items the Drug Name will be a drug treatment, but other intervention types are also admissible into the Drug Name field, as there are cases where variants may be associated with increased response to other treatment types (e.g. Radiation Therapy, NCIT Code C15313)
- In some cases, as in the figure, multiple versions of the drug treatment are available as NCIT terms. In this case, curators are advised to choose the basic drug name, in this case Sunitinib, unless the treatment specifies a special form of the drug is required. Well defined regimens such as FOLFOX (Code C11197) and FOLFIRI (Code C63593) are admissible Drug Names.

The Assertion data model

Fig. S35. Overview of CIViC Assertion entry form

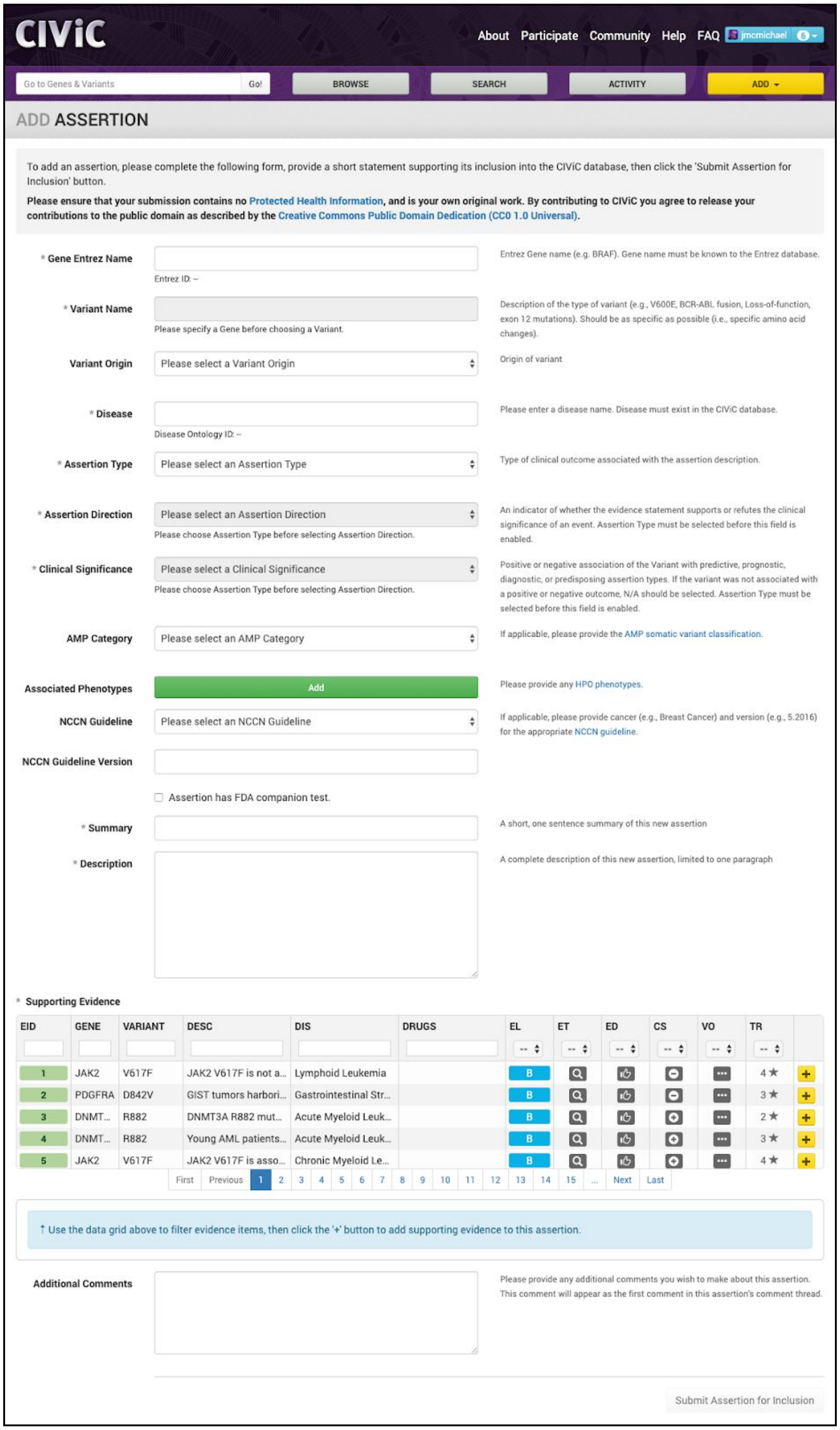

Curation Practices:

- The Supporting Evidence grid allows users to associate Evidence Items with Assertions. This can be accomplished by filtering on the provided fields (e.g., EID, Gene, Variant) and selecting the yellow plus sign on the right.
- A sufficient amount of evidence should be added to an Assertion so that the collection of Evidence Items represents that state of the field for the disease, variant, and type of Evidence Statement.
- As new evidence emerges which is relevant to a CIViC Variant which already has an accepted Assertion, then additional Evidence Items (EIDs) can be curated from this new evidence and added to the Supporting EIDs for the existing Assertion, potentially raising its ACMG classification or AMP Tier and Level.
- Practice guidelines, which are standard for the field from which the Assertion is derived, should be thoroughly consulted. Approved disease stage, and approved treatment lines should be outlined in the Assertion Description, if applicable.
- Approved companion diagnostics (e.g. Vysis Break-Apart Fish diagnostic for ALK-fusions) should be listed in the Assertion Description.

Examples of Assertion Variant Origin

Fig. S36. Example of curation of Variant Origin

This is an example of an Assertion whereby the Variant Origin reflects the Evidence Items supporting the Assertion. In this example, the *VHL* - Q195* (c.583C>T) Variant is implicated as Pathogenic based on ACMG Codes PVS1, PM2, and PP4. All Evidence Items supporting this Assertion are Case Studies from families that implicate this specific germline variant in the resulting disease.

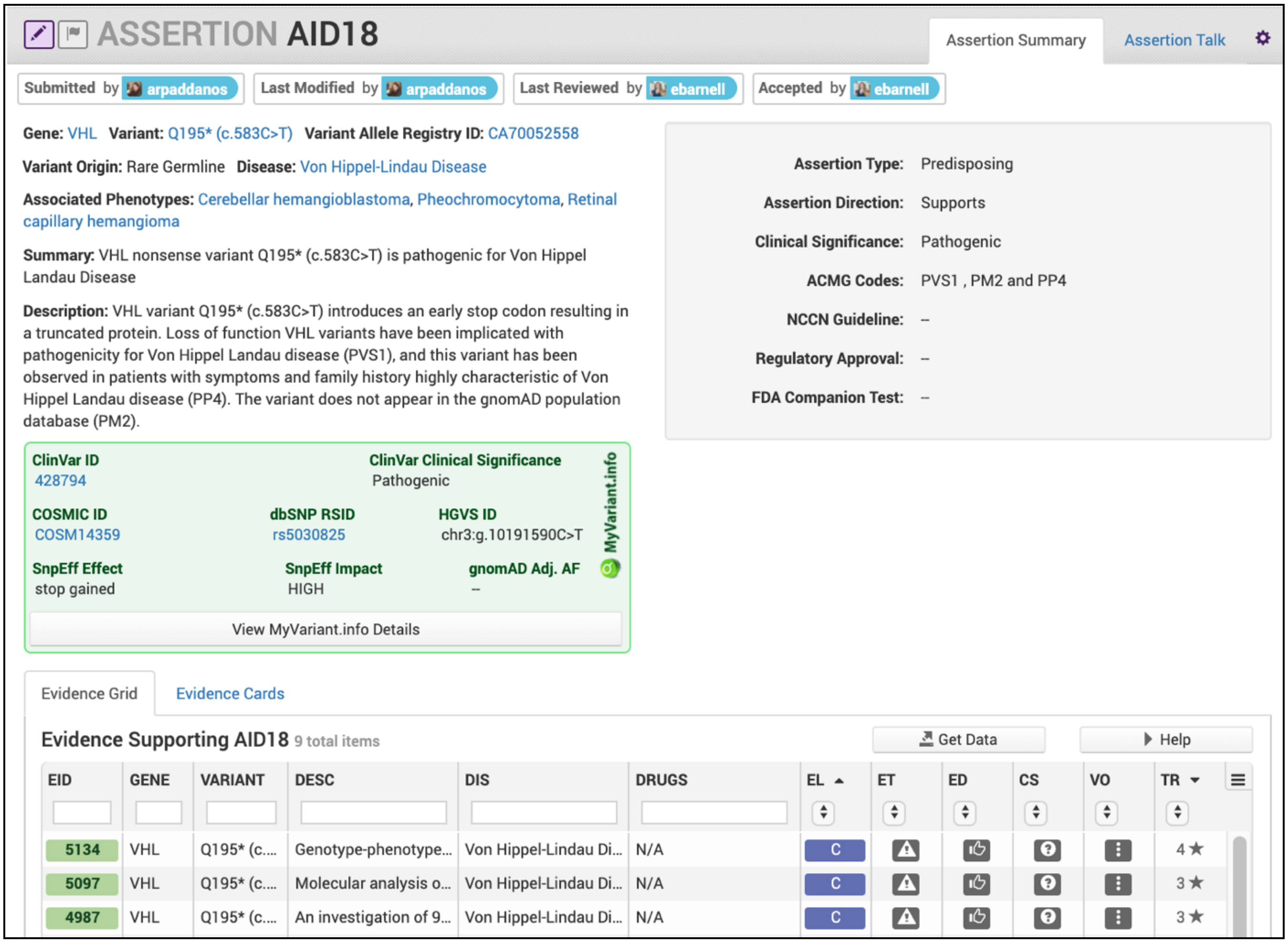

Curation Practices:

- Typically, Rare Germline or Common Germline Variants are associated with Predisposing Assertions, however, this is not always the case.
- Clicking on the Evidence Cards tab of the Evidence Grid will display all underlying Evidence Items in their entirety.
- Predisposing Rare Germline Assertions utilize ACMG criteria for evaluation of Clinical Significance (See **Fig. S41**)

Examples of Disease in Assertions

Fig. S37. Selection of Disease Type for Assertions

Only one Disease is permitted for each Assertion. It is recommended that the Disease be as specific as possible while still holding true for all Evidence Items associated with the Assertion. The example Assertion below is composed of Evidence Items whose Disease is either “Melanoma” or “Skin Melanoma”. At the Assertion level, the less specific Disease type (Melanoma) is selected to represent all supporting Evidence Items.

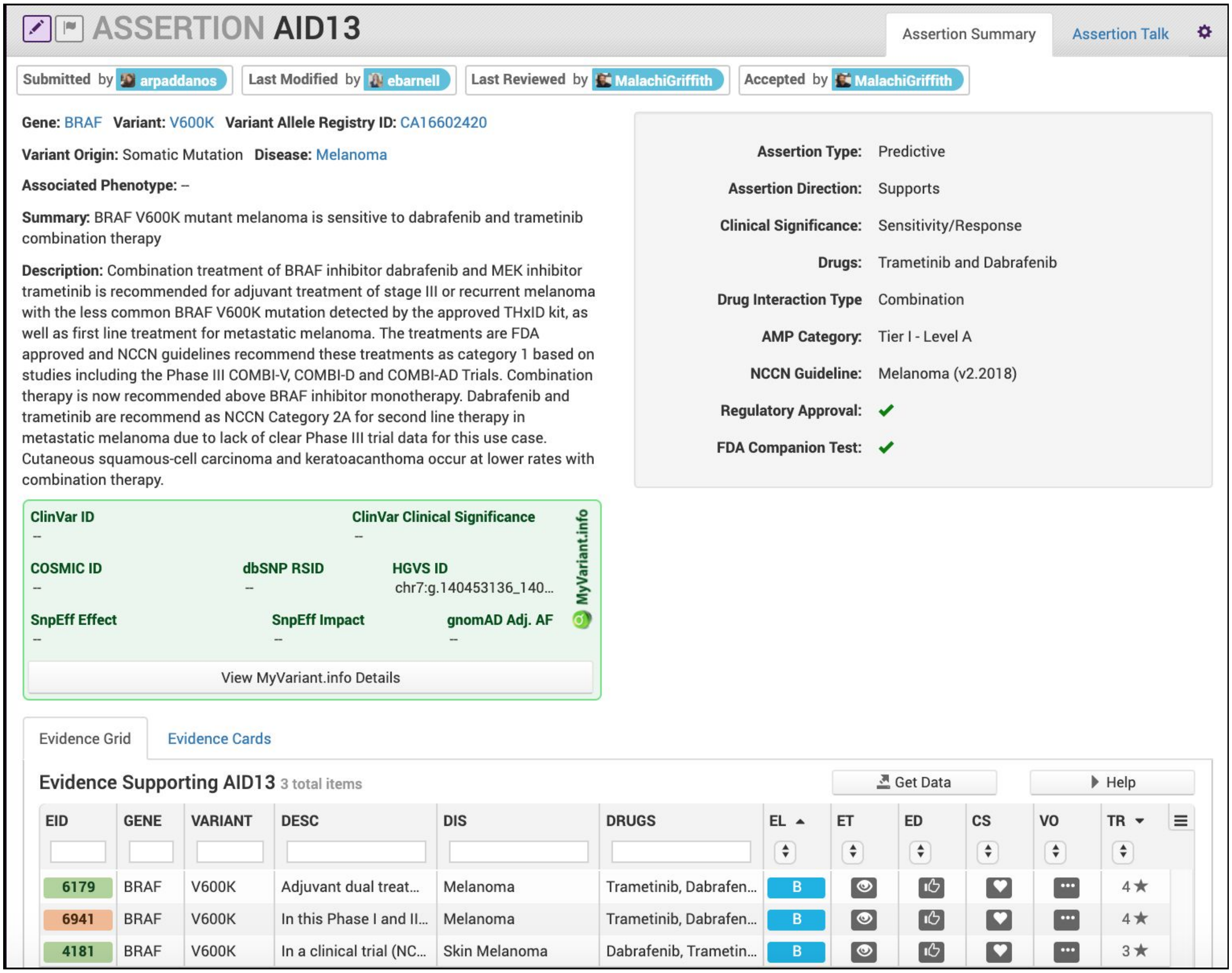

Curation Practices:

- Disease stage to which the assertion applies, as well as the line of treatment (e.g. first line, salvage, etc) should be made explicit in the Assertion Description. While the body of supporting Evidence Items may be derived from studies with differing patient populations with regard to stage and line of treatment, as well as preclinical studies in disease models, practice guidelines (e.g. NCCN etc) should be consulted for approved use cases to be described in the Assertion.

Examples of Assertions

Fig. S38. Predictive Assertion

This Predictive Assertion describes that *BRAF* V600E confers sensitivity to combination therapy of dabrafenib and trametinib for patients with melanoma. The AMP-ASCO-CAP Category is Tier I - Level A for this CIViC Variant, Disease and Drug Sensitivity Assertion. This AMP-ASCO-CAP Tiering is a consequence of the presence of this variant and treatment in the Melanoma NCCN Guidelines (v2.2018).

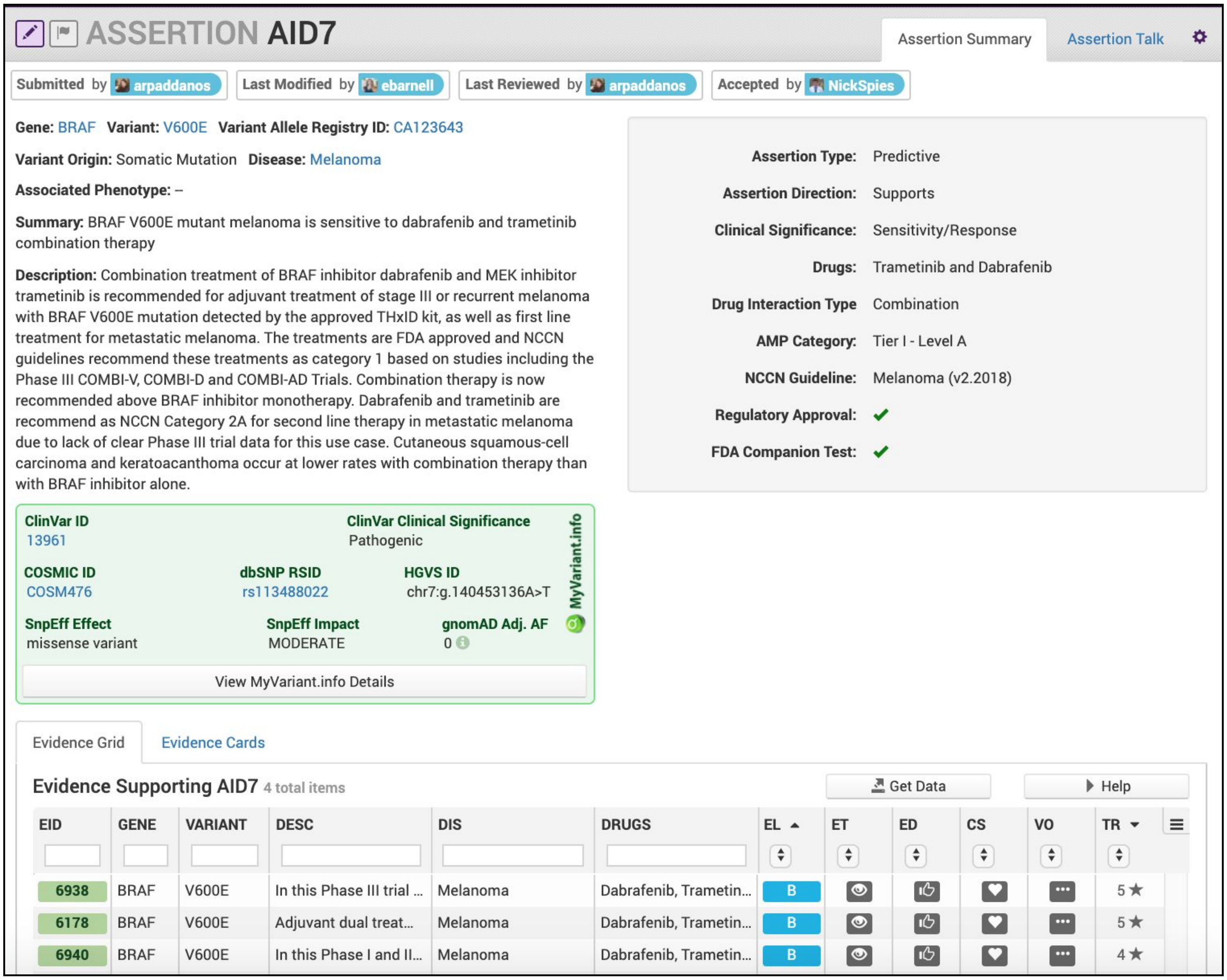

Curation Practices:

- All Evidence Items relevant to the Assertion should be associated to it, even if they disagree with the Assertion Summary. Disagreements can be discussed in the Description section and rationale for discounting discrepant evidence should be recounted.
- AMP Level and Tier should be associated with each Predictive, Diagnostic and Prognostic Assertion (Li et al. 2017). For methods on assigning AMP-ASCO-CAP Tier / Level, See **Figure 5A**.
- Practice guidelines, which are standard for the field from which the Assertion is derived, should be thoroughly consulted. Approved disease stage, and approved treatment lines should be outlined in the Assertion Description, if they are in place in guidelines. It is recommended to consult guidelines (e.g. NCCN) first, to allow them to structure creation of high Tier Assertions.
- Lower AMP-ASCO-CAP Tier Assertions can be written in the absence of practice guidelines, using Curator and Editor's overviews of the field. It is recommended to have a good overview of recent reviews in this case.

Fig. S39. Exemplary Prognostic Assertions

The figure below shows a Prognostic Assertion with an exemplary Assertion Summary and Assertion Description. In this example, the Assertion describes that the *BRAF* V600E Variant confers poor outcome for patients with colorectal cancer. This variant has an associated FDA companion diagnostic test, is listed in the NCCN Guidelines for colorectal cancer (v2.2017), and falls under the Tier I - Level A AMP category.

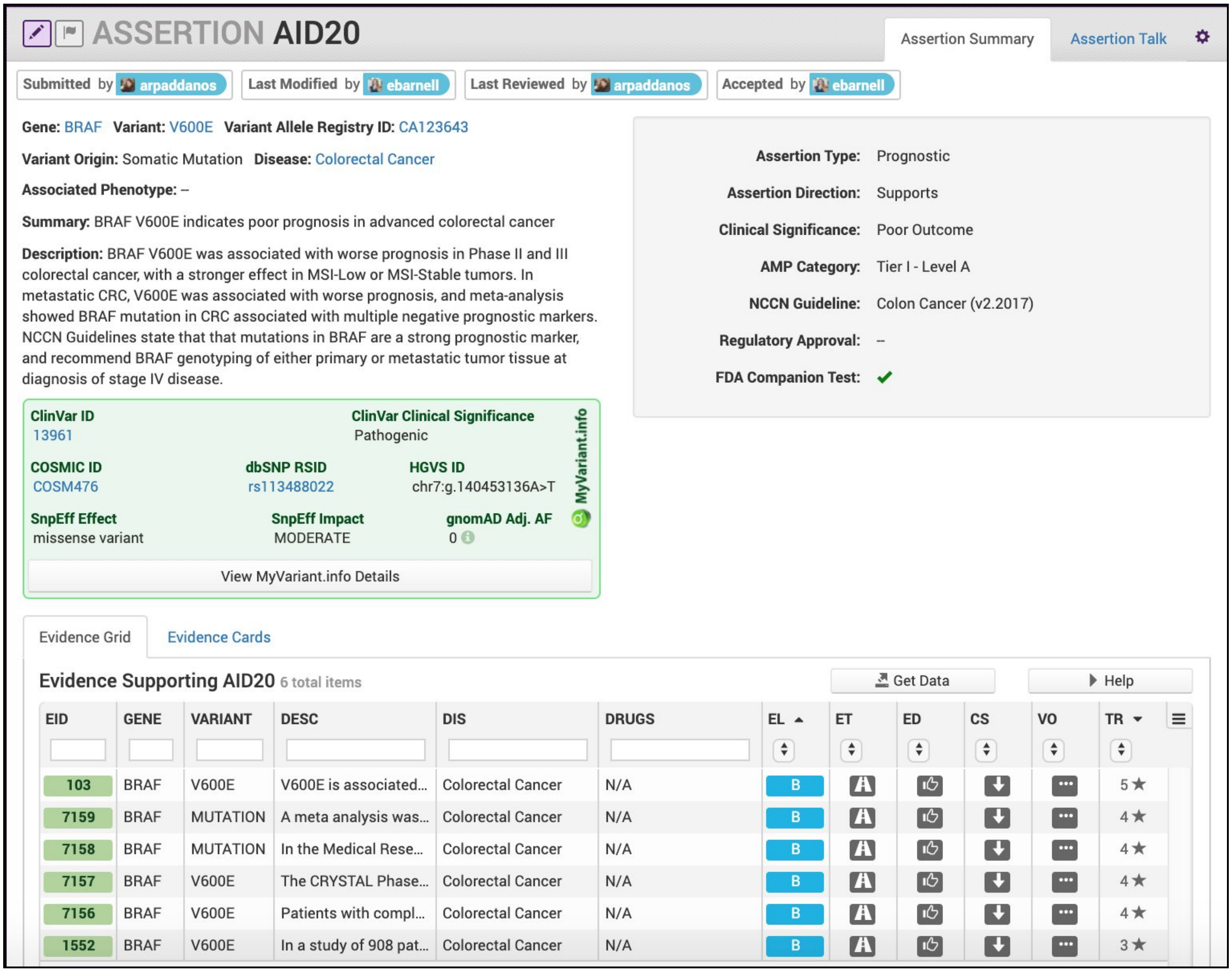

Curation Practices:

- All Evidence Items relevant to the Assertion should be associated to it as supporting evidence, even if they disagree with the Assertion Summary. Disagreements can be discussed in the Description section and rationale for discounting discrepant evidence should be recounted.
- Prognostic evidence in CIViC demonstrates variant association with better or worse patient outcome in a general manner, that is independent of any specific treatment context. Therefore, a larger collection of evidence showing similar prognostic outcomes under a range of different treatment or untreated regimes is ideal.
- Application of AMP-ASCO-CAP Tier and Level (Li et al. 2017) is dependant on practice guidelines (e.g. NCCN) ascribing prognostic value to the variant for the given disease, or failing this, the Tier will depend on the quality and level of evidence supporting the Assertion (**Figure 5A**).

Fig. S40. Exemplary Diagnostic Assertions

Below is an example of a Diagnostic Assertion with an exemplary Assertion Summary and Assertion Description. In this example, the Assertion describes how an in-frame fusion between *DNAJB1* and *PRKACA* can be used to diagnose a specific subtype of hepatocellular carcinoma (HCC). Presence of this fusion can be used to clarify that the patient has fibrolamellar HCC.

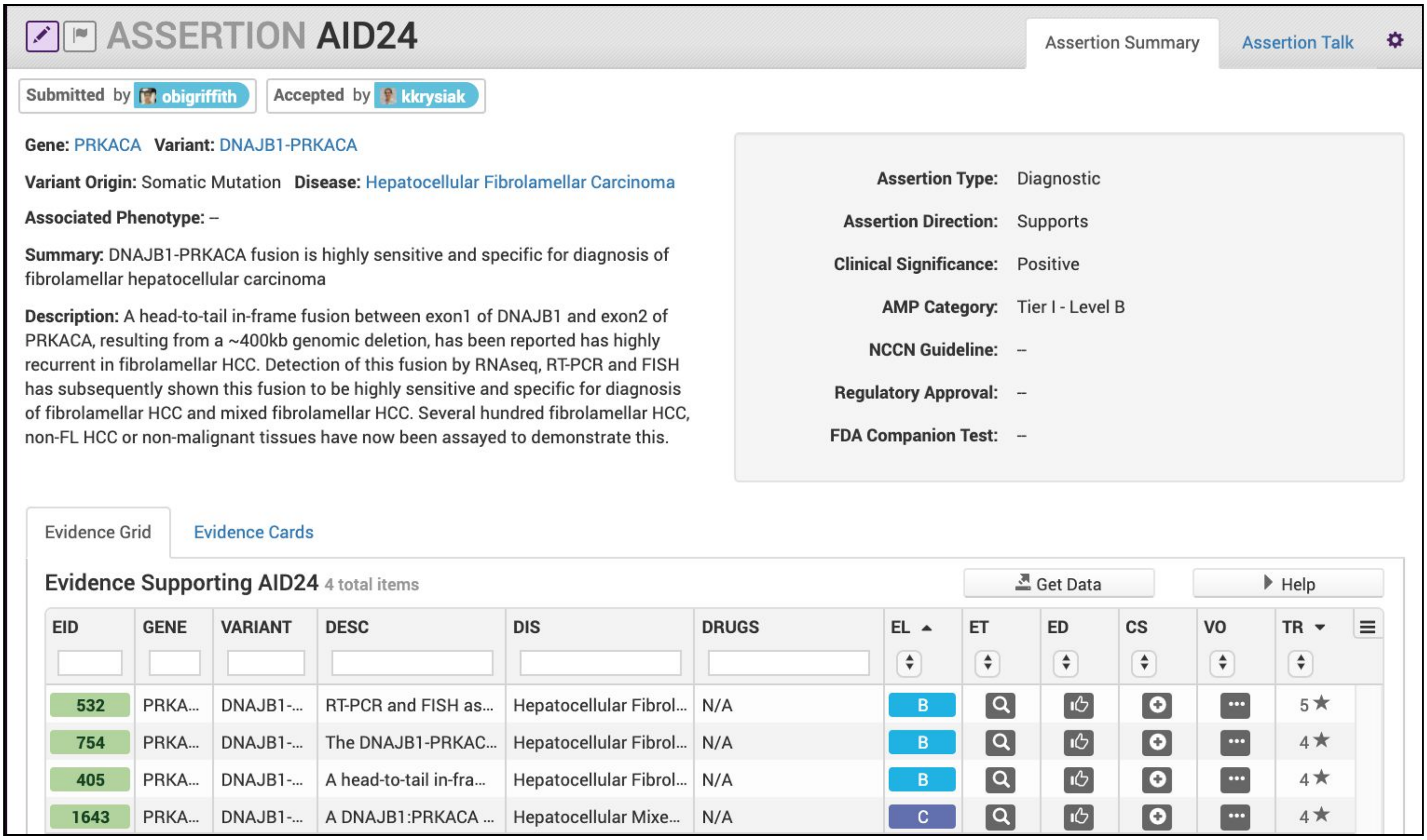

Curation Practices:

- All Evidence Items relevant to the Assertion should be associated to it as supporting evidence, even if they disagree with the Assertion Summary. Disagreements can be discussed in the Description section and rationale for discounting discrepant evidence should be recounted.
- The evidence supporting the Assertion should sufficiently cover what is known regarding the diagnostic power for the variant in the specific disease context.
- For Tier I Level A Diagnostic Assertions, details from relevant practice guidelines should be given, along with any additional specific information which is applicable (e.g., disease stage).
- Lower Tier and Evidence Level Assertions may be created for Diagnostic CIViC Variants not currently in practice guidelines. Variants backed by stronger clinical data may be Tier I Level B as above. Variants with smaller amounts of evidence for diagnostic potential will receive lower Tiers and Evidence Levels (**Figure 5A**).

Fig. S41. Exemplary Predisposing Assertion

Below is an example of a Predisposing Assertion. In this example, an inframe deletion repeatedly observed in the literature is considered pathogenic for Von Hippel-Lindau Disease. Utilizing the ACMG guidelines (Richards et al. 2015), evidence codes were assembled from the literature (PP1, PS2) and Variant-level information (PM4, PM2) to be categorized as Pathogenic. Specific evidence is associated with codes in the Description and all evidence evaluated when producing the Assertion is associated with the Assertion.

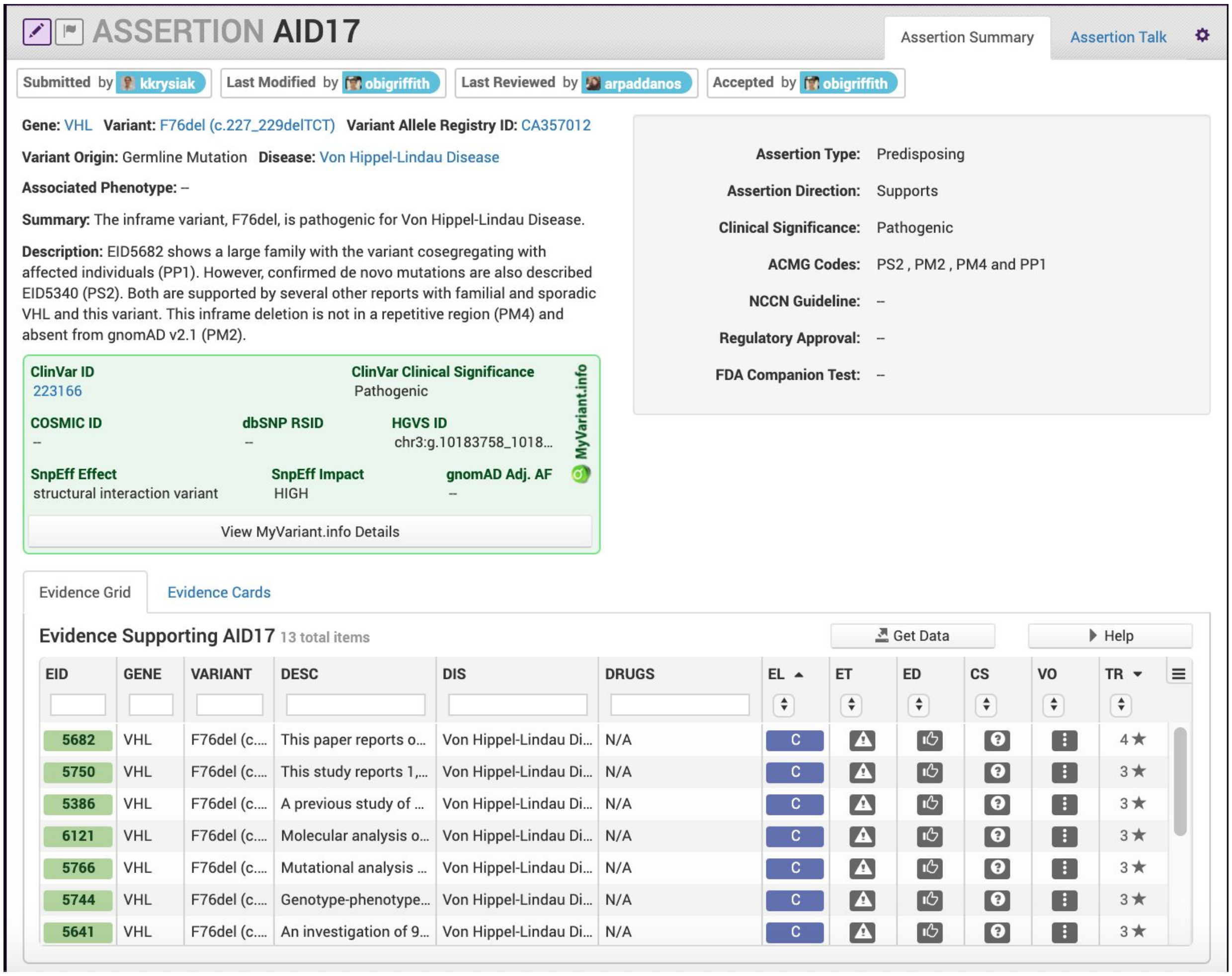

Curation Practices:

- ACMG-AMP codes (Richards et al. 2015) supporting the Predisposing Assertion are derived from supporting Evidence Items, and other sources such as population databases (See **Figure 5B**). Any evidence codes applied should be explained in the Description section, allowing others to rapidly re-evaluate the evidence used.
- All Evidence Items relevant to the Assertion should be associated, even if they disagree with the Assertion Summary. Disagreements can be discussed in the Description section and rationale for discounting discrepant evidence should be recounted.
- Thoroughly evaluated Assertions can have a clinical significance of Variant of Unknown Significance using ACMG-AMP criteria. This permits other users to quickly re-evaluate this variant in the context of new evidence, potentially leading to reclassification, but reducing future curation burden if the variant is observed again.
- If supporting EIDs contain partially met ACMG criteria, then the Curator will assess whether the instances of partial support for the given ACMG code across different EIDs is sufficient for its inclusion in the Assertion and therefore the calculation for the ACMG classification.

Examples of Assertion Supporting Evidence

Fig. S42. Requirements for an Assertion to be accepted

A complete list of Assertions can be found on the Assertions tab of the Browse page.

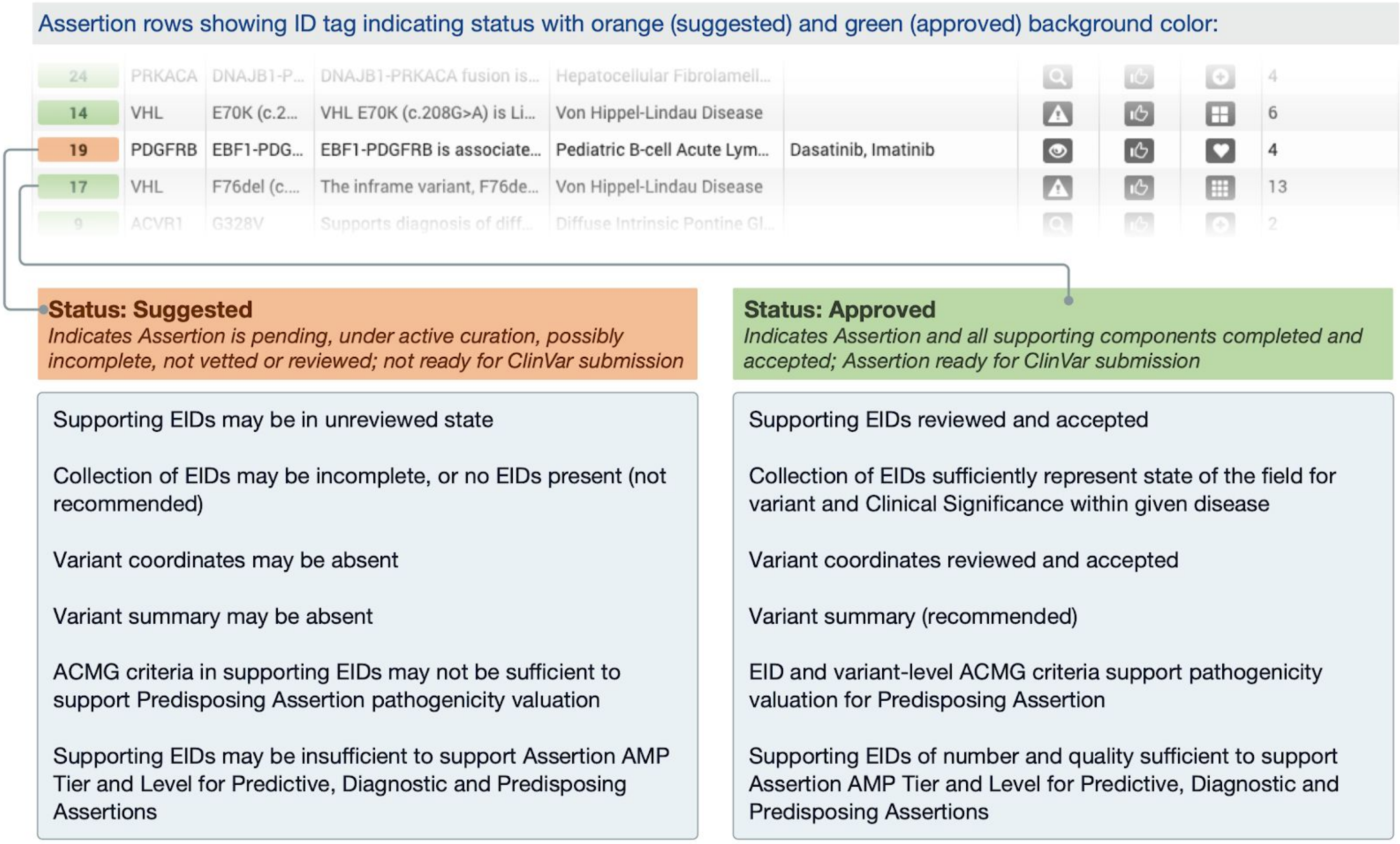

Curation Practices:

- Assertions can be curated without being associated with any Evidence Items; however, they cannot be accepted until at least one Evidence Item is linked to the Assertion.
- Minimal evidence requirements for each AMP-ASCO-CAP Tier are listed in **Fig. S10**. Note that these evidence requirements are not necessarily sufficient for the Assertion to be accepted, as the supporting EID collection for an accepted Assertion should summarize the important aspects of what is known for the CIViC Variant in the context of the given disease, and with respect to the Assertion's Clinical Significance.
- A new Assertion can only be created for a variant/gene that already exists in CIViC.
- Evidence Items that are associated with Assertions must be accepted prior to the Assertion being accepted; however, these Evidence Items may still be revised and edited after the Assertion has been accepted.

Fig. S43. Comparing CIViC Predictive Clinical Significance to other classification systems

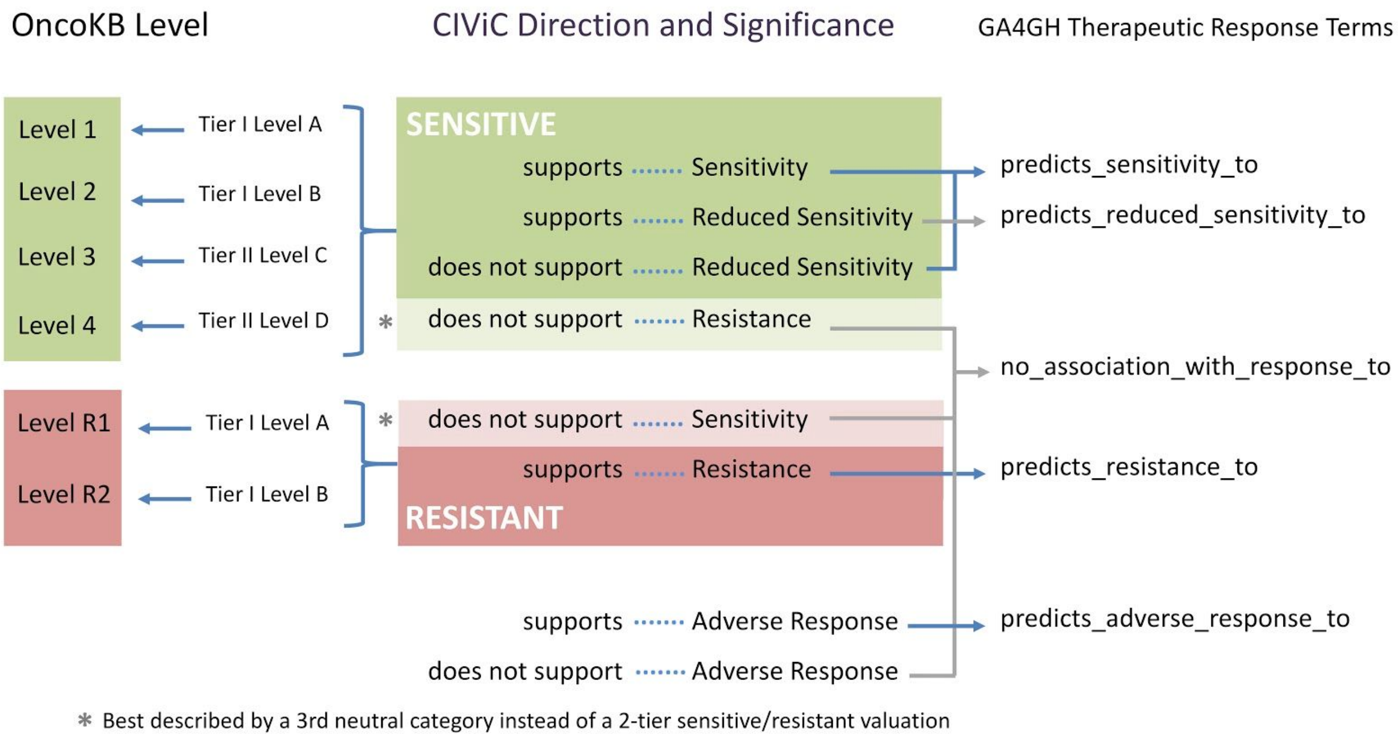

The CIViC Predictive (Therapeutic) Clinical Significance is a clinical annotation for cancer variants which can be compared across different systems for structure annotation and variant tiering.

- To the left of are listed AMP-ASCO-CAP 2017 Tier and Level, which are incorporated into CIViC in the form of Assertion (**Figure 5a**). To the left of that are OncoKB Levels used for tiering variants, which can be related to CIViC through CIViC Assertions labeled with AMP-ASCO-CAP Tier and Level.
- Note that some CIViC Assertion Direction and Significance values which are consistent with an overall valuation of Sensitive or Resistant in the central block of the figure (does not support Resistance and does not support Sensitivity/Response) do not map well into the OncoKB Levels.
- To the right of the central block consisting of CIViC Predictive structured annotation are comparisons with GA4GH Therapeutic Response Terms, where arrows point from CIViC terms to the current state of those of GA4GH, and indicate when multiple CIViC terms map into one from GA4GH. This set of GA4GH terms is not finalized and public discussion of them is accessible: https://github.com/ga4gh-gks/variant-annotation-model/issues/31#issuecomment-493514331

Supplementary Tables

Table S1. Roles in CIViC

NAME	DESCRIPTION	NOTES
User	Any person engaging with the CIViC knowledgebase through the interface or API or downloaded dataset is a User. Users have unrestricted access to all CIViC data, with no restrictions on use. If a User wishes to contribute curation to CIViC, then they must create an account to gain the role of a Curator.	login not required for CIViC data access
Curator	Any person creating a free login to CIViC may curate new content by adding Evidence Items (EIDs), Assertions, and Source Suggestions or suggest revisions to existing content, including coordinates and Gene/Variant Summaries. Submitted, unmoderated EIDs and Assertions are visible to the public and displayed in orange. Submitted changes are only visible in the Talk tab of each element until moderated. Curators may also comment on content and Suggested Revisions as well as Flag entities with significant issues.	Self-selected CIViC role, login required
Editor	Editors are Curators who are granted added powers to engage in moderation of CIViC by accepting or rejecting EIDs, Assertions, and other suggested changes submitted by Curators or other Editors. Editors may also resolve Flags. Editors are unable to accept their own submissions or suggested revisions. Editors are drawn from Curators with experience and good competency with the CIViC knowledge model, and will undergo extra training before a decision is made to grant Editor status. Any experienced Curator may request to be considered for Editor. If the Curator's experience is deemed sufficient (relevant expertise, multiple accepted EIDs, and some level of curation of Variant fields) then the Curator will be considered for Editor training. Editors will have a PhD, MD, Master's, or other relevant background. Editors also have all the rights and privileges of Curators and their content must be reviewed by another Editor. Editors must supply an email address and fill out a Conflict of Interest statement.	Assigned role by CIViC, login required
Administrator	Administrators are certain members of the CIViC team, including developers, given additional ability to edit parts of CIViC not accessible to curation, such as the creation of Organizations and the addition of Curators to those Organizations, done at the request of those Curators and Organizations. Administrators have full editorial powers, and are therefore by default Editors. Some Administrators are part of the CIViC development team and do not engage in moderation of CIViC.	Assigned role by CIViC, login required
Domain Expert	Domain Experts can have any CIViC role but are assigned special status. They are usually scientists or physician scientists (PhD or MD), with demonstrated expertise (publication records) relevant to curation of knowledge for precision medicine in specific areas of cancer. Domain Experts will typically take responsibility for curating the literature related to a specific cancer type, gene/pathway, or mutation type.	CIViC designation, login required

Table S2. Activities in CIViC

NAME	DESCRIPTION	COMMUNITY RECOGNITION
Commenting	Sources, Flags and editable CIViC entities allow Curators and Editors to add comments. Comments can be used, for example, to discuss a suggested revision or the reason for flagging an entity. Any CIViC Curator (or Editor) may leave comments in CIViC.	'Commentor' badges are awarded, and a 'Top Commentor' leaderboard is displayed on the Community page.
Suggesting	Any CIViC Curator (and therefore Editor) may suggest a Source for curation if they feel it contains material sufficient to warrant a new Evidence Item.	'Suggestor' badges are awarded.
Submitting	Any CIViC Curator (and therefore Editor) may submit Evidence Items and Assertions to CIViC, and these actions count towards the Submissions total.	'Submittor' badges are awarded, and a 'Top Submittor' leaderboard is displayed on the Community page.
Revising	Revisions to any curatable field in CIViC can be submitted to CIViC by Curators or Editors. These Revisions will then await moderation by Editors (an Editor may not moderate their own Revisions)	'Revisor' badges are awarded, and a 'Top Revisor' leaderboard is displayed on the Community page.
Moderating	CIViC Editors have the ability to accept or reject Evidence Items, Assertions, and Revisions to any field in CIViC as long as they themselves did not submit them.	'Moderator' badges are awarded, and a 'Top Moderator' leaderboard is displayed on the Community page.

- Activities in CIViC are actions that Curators and Editors are able to do on elements of the knowledgebase when logged in. For each Curator or Editor, total actions are counted, and actions of each type listed in the table are also kept track of seperately, and recorded on the CIViC Community page as activities. Badges for these activities are also awarded to Curators and Editors, and visible on their profile page. Additional badges awarded for specialized actions are:
    - The Gene Specialist Badge, for submitting multiple Evidence Items to CIViC Variants under the same gene
    - The Disease Specialist Badge, for submitting multiple Evidence Items for the same Disease
    - The Biographer Badge, for filling out personal fields in the Curator or Editor's profile page.
- Flagging exists as an activity in CIViC, which is not associated with a Badge. Flag icons are found across all curatable areas of the CIViC interface, and exist next to the pencil icon used to open the curation interface (See **Table S3**).

Table S3. General curation notation for all items within CIViC

Button/Icon	Name	Description
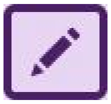	Edit Button	Selection of the purple pencil in the upper left corner allows Curators to edit an entity.
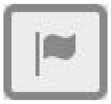	Flag Button	Clicking the flag in the upper left corner allows a Curator to flag an entity for additional review.
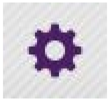	Preferences Button	Users can subscribe to curation updates for a specific entity by clicking the gear icon in the top right of the screen.
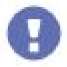	Pending Revisions Icon	Hovering over the exclamation mark icon will show pending revisions for the entity.

Table S4. Examples of Variants supported by the CIViC interface

Variant name	Variant type (Sequence Ontology)	Demonstrates support of…
DPYD*2A Homozygosity	Splice Donor Variant	pharmacogenomic nomenclature
ALK Fusions	Transcript Fusion	fusions with an unknown partner common in FISH techniques
EML4-ALK	Transcript Fusion	specific gene fusions
EML4-ALK E6;A20	Transcript Fusion	fusions with known specific exon boundaries / specific fusion isoforms
BCR-ABL T315I	Missense Variant	specific variants in the context of other variants
CDKN2A Promoter Hypermethylation	N/A	epigenetic modifications
p16 Expression	N/A	expression changes
rs3814960	5 Prime UTR Exon Variant	Variants not directly associated with altered gene product
FLT3-ITD	Inframe Insertion	imprecise insertions with shared consequences
KIT Exon 11 Mutation	Coding Sequence Variant	categorical variants covering specific transcriptional boundaries
TP53 DNA Binding Domain Mutation	DNA Binding Site	categorical variants covering specific functional boundaries
MET Exon 14 Skipping Mutation	Exon Loss Variant	categorical variants covering specific transcriptional consequences
BRCA Loss-of-function	Loss of Function Variant	categorical variants covering specific functional consequences
BRAF V600E	Protein Altering Variant	a specific amino acid change which includes all possible DNA changes which result in this missense change
BRAF V600	Protein Altering Variant	categorical variants involving a single amino acid
BRAF non-V600	Protein Altering Variant	categorical variants excluding a common hotspot
VHL S65W (c.194C>G)	Missense Variant	precise base pair resolution variants
ERBB2 SERUM LEVELS	N/A	sample-specific protein levels measured by assays such as ELISA
IGF1R NUCLEAR EXPRESSION	N/A	cellular compartment-specific expression measured by assays such as immunohistochemistry (IHC)
STAT3 SH2 Domain Mutation	Coding Sequence Variant	variants within a specific domain

Table S5. Examples of classification Sequence Ontology Term classifications

Sequence Ontology Term	Sequence Ontology Definition	Examples
missense_variant	A sequence variant, that changes one or more bases, resulting in a different amino acid sequence but where the length is preserved.	G12D
stop_gained	A sequence variant whereby at least one base of a codon is changed, resulting in a premature stop codon, leading to a shortened polypeptide.	R130*
protein_altering_variant	A sequence variant which is predicted to change the protein encoded in the coding sequence.	G12<br>KINASE DOMAIN MUTATION
frameshift_truncation	A frameshift variant that causes the translational reading frame to be shortened relative to the reference feature.	v2288fs*1
inframe_deletion	An inframe non synonymous variant that deletes bases from the coding sequence.	DEL I843<br>V560DEL<br>DEL755-759
inframe_insertion	An inframe non synonymous variant that inserts bases into in the coding sequence.	P780INS<br>M77INSAYVM<br>ITD
[gene_variant<br><br>OR transcript_variant]<br><br>AND<br><br>[loss_of_function_variant<br><br>OR<br><br>gain_of_function_variant]	[gene_variant: A sequence variant where the structure of the gene is changed.<br>OR<br>transcript_variant: A sequence variant that changes the structure of the transcript]<br><br>AND<br><br>[loss_of_function_variant: A sequence variant whereby the gene product has diminished or abolished function.<br><br>OR<br><br>gain_of_function_variant: A sequence variant whereby new or enhanced function is conferred on the gene product.]	MUTATION
exon_variant	A sequence variant that changes exon sequence.	EXON 10 MUTATION
transcript_fusion<br><br>OR RARELY<br><br>gene_fusion	transcript_fusion: A feature fusion where the deletion brings together transcript regions.<br><br>OR RARELY<br><br>gene_fusion: A sequence variant whereby a two genes have become joined.	EML4-ALK<br>ALK FUSIONS
transcript_fusion<br><br>AND<br><br>missense_variant	transcript_fusion: A feature fusion where the deletion brings together transcript regions.<br><br>AND<br><br>missense_variant: A sequence variant that changes one or more bases, resulting in a different amino acid sequence but where the length is preserved.	ELM4-ALK G1269A

transcript_translocation<br><br>OR<br><br>feature_translocation<br><br>OR<br><br>transcript_fusion	transcript_translocation: A feature translocation where the region contains a transcript.<br><br>OR<br><br>feature_translocation: A sequence variant, caused by an alteration of the genomic sequence, where the structural change, a translocation, is greater than the extent of the underlying genomic features.<br><br>OR<br><br>transcript_fusion: A feature fusion where the deletion brings together transcript regions.	REARRANGEMENT
wild_type	An attribute describing sequence with the genotype found in nature and/or standard laboratory stock.	WILD TYPE
loss_of_heterozygosity	A functional variant whereby the sequence alteration causes a loss of function of one allele of a gene.	LOH
transcript_amplification	A feature amplification of a region containing a transcript.	AMPLIFICATION
transcript_ablation	A feature ablation whereby the deleted region includes a transcript feature.	DELETION
copy_number_change	A sequence variant where copies of a feature (CNV) are either increased or decreased.	COPY NUMBER VARIATION
loss_of_function_variant	A sequence variant whereby the gene product has diminished or abolished function.	LOSS-OF-FUNCTION
Loss_of_function_variant OR transcript_ablation	loss_of_fuction_variant: A sequence variant whereby the gene product has diminished or abolished function.<br><br>transcript_ablation: A feature ablation whereby the deleted region includes a transcript feature.	LOSS
exon_loss_variant	A sequence variant whereby an exon is lost from the transcript.	EXON 14 SKIPPING MUTATION
5_prime_UTR_variant	A UTR variant of the 5' UTR.	5’ UTR MUTATION
3_prime_UTR_variant	A UTR variant of the 3' UTR.	3’ UTR MUTATION
N/A		EXPRESSION<br>NUCLEAR EXPRESSION<br>CYTOPLASMIC EXPRESSION<br>OVEREXPRESSION<br>UNDEREXPRESSION<br>METHYLATION<br>PROMOTER METHYLATION<br>PROMOTER HYPERMETHYLATION

Table S6. Use cases for curation of Predictive, Prognostic and Diagnostic Evidence Items with different Evidence Direction, and in different contexts including primary and secondary mutations

Context	Direction/ Significance	Preclinical (Level D)	Case Study (Level C)	Clinical (Level B)	Comment
Clinical or preclinical studies testing for the ability of a variant to promote sensitivity to a given treatment in a specific disease context, where patient populations or preclinical samples without the variant may be used as controls. (Primary sensitizing mutation)	Predictive: Supports Sensitivity or Response	Controlled experiments in preclinical models demonstrating sensitization of variant in comparison to wildtype.	Clinical observation of a patient suggesting variant association with a treatment response, where treatment response is not observed / expected for wildtype	Phase I, II, III or other clinical study demonstrating a significant association of the variant with treatment sensitivity, possibly compared to wildtype control population.	Associates a given CIViC Variant and with drug or other treatment sensitivity or response. If work described in the evidence item has been directly cited to support regulatory approval or practice guideline, then evidence item is labeled as Validated Level A.
	Predictive: Does Not Support Sensitivity or Response	Controlled experiments in preclinical models demonstrating no sensitization of variant in comparison to wildtype.	Clinical observation of a patient with variant responding to treatment as would a wildtype patient. I.e. no observation of a potential sensitizing role for the variant.	Phase I, II, III or other clinical study with failure to show significant treatment sensitivity associated with the variant, possibly compared to wildtype/control population.	Variant responds the same to treatment as in the non-sensitized wildtype context.
Clinical or preclinical studies testing for the ability of a variant to promote resistance to a particular treatment in a specific disease context, where the background (without variant) is sensitive to the treatment (i.e. studies designed to test for a secondary resistance mutation).	Predictive: Supports Resistance	Controlled experiments for particular treatment in preclinical models demonstrating loss of sensitization with the variant in comparison to sensitization in the absence of variant.	Clinical observation of patient suggesting the variant is associated with treatment resistance (e.g. variant might appear in sequencing after a previously responsive patient develops resistance to the treatment).	Phase I or higher phase study demonstrating statistically significant variant association with resistance to a given treatment.	Introduction of variant is associated with loss of sensitivity observed in the background state without variant. If the work described in the evidence item has been directly cited to support regulatory approval or in practice guidelines, then the evidence item is labeled as Validated Level A.
	Predictive: Does Not Support Resistance	Controlled preclinical experiments for a particular treatment that demonstrate no loss of sensitivity to treatment when variant is present.	Observation of a patient with a given variant responding to treatment in the same way as a sensitized patient would be expected to.	Phase I or higher phase study demonstrating no statistical association with the variant and resistance to a given treatment.	This annotation is not equivalent to “Supports Sensitivity”, since the variant is not sensitizing, but instead is neutral, and does not affect the baseline sensitivity. The annotation is best used when guidelines suggest variants of the given type may induce resistance, such as *KRAS* mutations in colorectal cancer with respect to EGFR inhibitor treatment.

Table S6 Continued

Context	Direction/ Significance	Preclinical (Level D)	Case Study (Level C)	Clinical (Level B)	Comment
Comparison of a variant to a different, known sensitizing variant for a given treatment and specific disease. (Comparison of primary sensitizing mutations)	Predictive: Supports Reduced Sensitivity	Controlled experiments in preclinical models demonstrating lesser degree of sensitization of the variant in comparison to an established sensitizing variant, but increased sensitization over wildtype.	Supports Sensitivity/Response best used in this context	Clinical study showing statistically significant intermediate response for given variant between established sensitized variant and baseline wildtype gene.	In case studies (Level C Evidence) this Clinical Significance is not recommended due to lack of adequate numbers of controls for comparison. Note that Reduced Sensitivity is used to compare two variants, but not two treatment regimes.
	Predictive: Does Not Support Reduced Sensitivity	Supports Sensitivity/ Response recommended in this context	Supports Sensitivity/Response recommended in this context	Supports Sensitivity/Response recommended in this context	Does Not Support Reduced Sensitivity is not currently a recommended annotation, as no clinically relevant use case for the annotation is apparent.
Comparison of two different treatment types against the same variant, within the same disease context. (Different treatment types against a primary mutation)	Predictive: Supports Sensitivity	(Noninferiority) Controlled experiments in preclinical models demonstrating a variant responds equally well to a new treatment in comparison to established treatment for the variant.	NA	(Noninferiority) Clinical studies showing patients do not fare worse with a new variant-targeted treatment in comparison to established treatment for the given variant. This evidence supports sensitivity for the variant to the new treatment.	Case studies will not support this type of evidence since a single or small group of patients cannot generate the necessary statistical power for comparison. If work described in the evidence item has been directly cited in support of regulatory approval or practice guidelines, then the evidence item is labeled Validated Level A.
	Predictive: Does Not Support Sensitivity	Controlled experiments in preclinical models demonstrate lesser degree of sensitization of variant to a new treatment in comparison to established treatment for the variant.	NA	Clinical trials demonstrating lesser degree of sensitization of variant to a new treatment in comparison to established treatment for the variant. In this case, the evidence does not support sensitivity for the variant to the new treatment.	When curating Evidence Items from results which show a newer treatment is less effective than an existing treatment for a given variant, then the Predictive, Does Not Support Sensitivity annotation may be used for the variant and disease with respect to the new treatment. The comparison to existing treatment can be described in the evidence summary.

Table S6 Continued

Context	Direction/ Significance	Preclinical (Level D)	Case Study (Level C)	Clinical (Level B)	Comment
Observation of patients or preclinical systems with association to a specific disease type, which are positive for the given variant. Comparison to various control samples, may be performed. Publications citing guidelines also can be used.	Diagnostic: Supports Positive	Preclinical work suggesting association between variant and disease or disease subtype.	Observations of variant being present in a small number of patients with the given disease, and potential comparison to patients without variant or disease	Clinical observations in a patient population of significant variant association with a positive diagnosis of a given disease.	Evidence from publications describing practice guidelines or regulatory approval may be used for curation and labeled as Validated level A. Work suggesting novel disease classification based on or related to the given variant may be curated by experts in the field, but is not recommended for general curation. Submission of new terms to Disease Ontology should accompany such curation.
	Diagnostic: Does Not Support Positive	Preclinical work suggesting no association between variant and disease or disease subtype.	Observations demonstrating lack of variant in small number of patients with specified disease or variant presence in patients without the specified disease.	Clinical studies with statistical results showing variant cannot support positive diagnosis.	This annotation will generally be used in cases where the variant could be expected to have diagnostic significance (e.g. previous findings), so that reports to the contrary could hold clinical interest.
Observation of patients or preclinical systems with contraindication to a specific disease type, which are positive for the given variant. Comparison to various control samples may be performed.	Diagnostic: Supports Negative	Preclinical work suggesting variant associated with negative diagnosis.	Smaller numbers of patient observations suggest variant may be a contraindication for a specific disease.	Studies with statistically significant findings which suggest that variant may be added to exclusion criteria for a given disease.	See comments for Diagnostic: Supports Positive
	Diagnostic: Does Not Support Negative	Preclinical work suggesting lack of association between variant and negative diagnosis.	Smaller numbers of patient observations suggest variant is not a contraindication for a specific disease.	Studies with statistically significant findings suggesting that the variant has no diagnostic power for the given disease.	See comments for Diagnostic: Does Not Support Positive

Table S6 Continued

Context	Direction/ Significance	Preclinical (Level D)	Case Study (Level C)	Clinical (Level B)	Comment
Variant present in patient populations or preclinical samples directly associated with better outcome, or associated with known markers of better outcome.	Prognostic: Supports Better Outcome	Experiments in preclinical systems suggesting variant is associated with better outcome, for instance through demonstration of association with cellular markers of less aggressive disease.	Case study reports or smaller trial subgroups, where patients with the given variant show good outcomes with respect to a given clinical measure, or show other indications associated with better outcome (e.g. patient samples show markers of better outcome).	Clinical observation that patient subgroups with the variant have better outcomes than patients without the variant, and that this result is not specific to a particular treatment type. Statistically significant results are ideal here.	If work described in the evidence has been directly cited to support regulatory approval or practice guidelines, then the Evidence Item is labeled Validated Level A. Prognostic evidence refers to better or worse outcome associated with a specific variant and disease, which is shown to occur regardless of specific treatment context.
	Prognostic: Does Not Support Better Outcome	Controlled preclinical experiments showing variant lacks association with better outcome.	Case study reports or trial subgroups with small numbers of patients with the given variant, that do not suggest a better outcome.	Clinical patient data which show no significant association of variant with better outcome in comparison to patients without variant.	This annotation will generally be used in cases where the variant could be expected to have prognostic significance (e.g. previous findings), so that reports to the contrary could hold clinical interest.
Variant present in patient populations or preclinical samples directly associated with poor outcome, or associated with known markers of poor outcome.	Prognostic: Supports Poor Outcome	Preclinical work suggesting association between variant and indicators of poor prognosis such as proliferative biomarkers, (e.g. Ki-67).	Case study reports or trial subgroups with small numbers of patients with the given variant, which suggest a poor outcome. Patient samples may show markers associated with a worse outcome.	Clinical observation that patient subgroups with the variant have significantly worse outcomes by some clinical measure, not specific to a particular treatment context.	See comments for Prognostic: Supports Better Outcome
	Prognostic Does Not Support Poor Outcome	Preclinical work that suggests lack of association with variant and indicators of poor prognosis such as proliferative biomarkers.	Case study reports or trial subgroups with smaller numbers of patients with the given variant that do not suggest a poor outcome.	Clinical patient data which show no significant association of variant with poor outcome, in comparison to patients without variant.	See comments for Prognostic Does Not Support Better Outcome

Table S6 Continued

This table presents various scenarios or contexts a Curator might find themselves attempting to curate when dealing with Predictive (Therapeutic) or other clinical evidence. These evidence scenarios make up the first column (**Context**). The next column (**Direction/Significance**) gives specific Clinical Significance annotations which could be chosen to appropriately capture and curate the evidence. The next three columns (**Preclinical**, **Case Study**, **Clinical**) give an example of what evidence might look like for each scenario. The final column (**Comment**) gives further clarification to the fields in the corresponding row.

- Usually, in cases where a putative secondary resistance variant on a sensitive background does not induce resistance, we choose the "Does Not Support Resistance" annotation, such as in EID7611, where a patient with ALK-rearranged lung cancer and the L1196M resistance mutation is given ceritinib, and a response is seen. In rare cases, a secondary variant on a primary sensitizing background may yield increased response, such as in EID7603, where secondary ceritinib resistance mutation L1198F, on an ALK-FUSION background, shows an increased response to crizotinib over ALK-FUSION alone. Thus here the "Supports Sensitivity to Crizotinib" annotation is chosen.
- The context of a secondary mutation being tested for potential resistance also impacts the Variant Name choice in CIViC. In some cases the secondary variant acts in a fashion that is generally independent of a class of primary sensitizing mutation. An example of this is T790M which acts in a secondary fashion with respect to primary 1st generation tyrosine kinase inhibitor (TKI)-sensitizing EGFR mutations. In this case the secondary resistance mutation can be simply and independently represented in CIViC with Variant Name T790M. In other cases, such as secondary ALK mutations on a sensitizing ALK-FUSION background, we choose to name this type of CIViC Variant ALK-FUSION G1269A, or ALK-FUSION F1245C, and so on, to distinguish this from cases where these ALK mutations might be assessed individually. This is done since some reports have studied wildtype ALK, or wildtype ALK with point mutations, in certain cancers. EID1269 is an example of this. So, while "TKI-sensitizing EGFR mutations" tend to be interchangeable with each other, with respect to the secondary T790M mutation, ALK and "ALK FUSIONS" are not interchangeable with each other, with respect to secondary ALK mutations such as F1245C. Therefore the choice of keeping the primary sensitizing mutation in the Variant Name or not can depend on what other research is happening with related variants. Often the nomenclature used in the field will inform the choice for Variant naming, and Curators are advised to have or gain familiarity with a field using reviews and primary literature before beginning in-depth curation for it.

Table S7. Definitions of Clinical Significance for all Evidence Types

Evidence Type	Clinical Significance	Definition / Curation Tips
Predictive *(Impact on therapeutic response)*	Sensitivity/Response	Associated with a clinical or preclinical response to treatment
	Resistance	Associated with clinical or preclinical resistance to treatment
	Adverse Response	Associated with an adverse response to drug treatment
	Reduced Sensitivity	Response to treatment is lower than seen in other treatment contexts
	N/A	Variant does not inform clinical action
Diagnostic *(Impact on diagnosis or disease subtype)*	Positive	Associated with diagnosis of disease or disease subtype
	Negative	Associated with lack of disease or disease subtype
Prognostic *(Impact on disease progression or patient survival)*	Better Outcome	Demonstrates better than expected clinical outcome
	Poor Outcome	Demonstrates worse than expected clinical outcome
	N/A	Variant does not inform clinical action
Predisposing *(Impact on disease susceptibility)*	N/A	Codes (ACMG criteria) from ACMG-AMP 2015 guidelines (Richards et al. 2015) are added to the Evidence Statement for this type of Evidence Item in lieu of a structured Clinical Significance annotation. Assertions summarizing collections of Evidence Items for a Variant add a 5-tier pathogenicity valuation based on the collection of ACMG criteria (See **Figure 5B**)
Functional *(Impact on biological alterations)*	Gain of Function	A sequence variant whereby enhanced function is conferred on the gene product
	Loss of Function	A sequence variant whereby the gene product has diminished or abolished function
	Unaltered Function	A sequence variant whereby the function of the gene product is unchanged
	Neomorphic	A sequence variant whereby the gene product creates a novel function
	Dominant Negative	A sequence variant whereby the gene product abolishes wild type allele function
	Unknown	A functional variant that cannot be precisely defined by gain-of-function, loss-of-function, neomorphic, dominant negative or unaltered function

Table S8. General guidelines and examples for Evidence Rating

A five-star Evidence Rating is used to evaluate the quality and amount of evidence supporting a particular Clinical Significance (i.e. Sensitivity, Resistance, etc) from a given publication or abstract, for a given Evidence Item. Each Evidence Item is given a rating, from 1 to 5 stars, based on the quality of the evidence the statement summarizes. The rating is specific to the data and conclusions within the Evidence Statement. The overall publication/study might be high quality, but the Evidence Statement may refer to a single conclusion in the study, and that part of the study might not be well supported. For example, the Evidence Item may relate to patients with a particular mutation, and the study might involve an impressive 500 patients, but if only 2 patients have the mutation in question, the quality rating may be low for this Evidence Statement.

★★★★★	Strong, well supported evidence from a lab or journal with respected academic standing. Experiments are well controlled, and results are clean and reproducible across multiple replicates. Evidence confirmed using independent methods. The study is statistically well powered.
★★★★	Strong, well supported evidence. Experiments are well controlled, and results are convincing. Any discrepancies from expected results are well-explained and not concerning.
★★★	Evidence is convincing, but not supported by a breadth of experiments. May be smaller scale projects, or novel results without many follow-up experiments. Discrepancies from expected results are explained and not concerning.
★★	Evidence is not well supported by experimental data, and little follow-up data is available. Experiments may lack proper controls, have small sample size, or are not statistically convincing.
★	Claim is not supported well by experimental evidence. Results are not reproducible, or have very small sample size. No follow-up is done to validate novel claims.

Table S9. Markdown and Macros.

Markdown is used to add emphasis, styling, images, and links to comments. Macros add links to specific CIViC users and entities. Below show the methods for employing Markdown and Macros to fields within the CIViC interface. These are particularly intended for comments on CIViC entities.

**Markdown**	
**Headers**	# This is an <h1> tag
	## This is an <h2> tag
	###### This is an <h6> tag
**Emphasis**	*This text will be italic*<br>_This will also be italic_
	**This text will be bold**<br>__This will also be bold__
	*Italics and boldface **can be** combined*
**Unordered List**	* Item 1<br>* Item 2<br>* Item 2a<br>* Item 2b<br>* Item 3
**Ordered Lists**	1. Item 1<br>2. Item 2<br>3. Item 3<br>* Item 3a<br>* Item 3b
**Images**	`![this cool diagram](http://site.com/images/cool.png)`<br>Format: `![Alt Text](url)`
**Links**	http://civic.genome.wustl.edu - automatic!<br>[CIViC](http://civic.genome.wustl.edu)
**Blockquotes**	As Charles Darwin said:<br><br>>It is not the strongest<br>>of the species that survive,<br>>nor the most intelligent,<br>>but the one most responsive to change.
**Inline Code**	I think you should use an<br>`<addr>` element here instead.

Macros		
**@ Mention suggestions**	Type '@', and the first few letters of a user's name, and CIViC will show you a dropdown menu of users with matching display names. Hit enter to insert the user mention link, which will display in the rendered comment as a link to the user's profile page, and generate a notification to the mentioned user.	
	@username	Select any user in the CIViC community
	@editors	Generate notification for all users that have Editor roles
	@admins	Generate notification for all users that have admin roles
**#ENTITY link macro**	'#' followed by an entity type abbreviation, and an entity ID will be displayed as a link to that entity's summary view.	
	#V123	Variant link
	#G123	Gene link
	#E123	Evidence Item link
	#VG123	VariantGroup link
	#R123	Revision link
	#A123	Assertion link
**Macro suggestion prompts**	Type '#', followed by an entity type abbreviation, a colon and search string, and CIViC will show you a list of entities with matching names (or summaries, in the case of evidence items). For example, entering '#V:v600' will display a list of variants with 'V600' in the names; '#G:BR' will display a list of Genes with the string 'BR' in the name. Select an entity to insert an #ENTITY string for that entity.	
	#G:[string]	Search for a gene by name
	#V:[string]	Search for a variant by name
	#VG:[string]	Search for a variant group by name
	#E:[string]	Search for an evidence item by summary
	#R:[string]	Search for a revision by change contents
	#A:[string]	Search for an assertion by summary

Table S10. Minimal Evidence Item requirements for AMP/ASCO/CAP-based CIViC Assertion to be accepted by CIViC Editors

AMP/ASCO/CAP Tier and Level	Minimal CIViC Evidence Items (EIDs) required
Tier I Level A or B	At least one A or B CIViC Evidence Level EID
Tier II Level C	At least one A or B CIViC Evidence Level EID or at least two C CIViC Evidence Leve EIDs
Tier II Level D	At least two C or D CIViC Evidence Level EIDs

References

Cotto, Kelsy C., Alex H. Wagner, Yang-Yang Feng, Susanna Kiwala, Adam C. Coffman, Gregory Spies, Alex Wollam, Nicholas C. Spies, Obi L. Griffith, and Malachi Griffith. 2018. “DGIdb 3.0: A Redesign and Expansion of the Drug–gene Interaction Database.” *Nucleic Acids Research* 46 (D1): D1068–73.

Griffith, Malachi, Obi L. Griffith, Adam C. Coffman, James V. Weible, Josh F. McMichael, Nicholas C. Spies, James Koval, et al. 2013. “DGIdb: Mining the Druggable Genome.” *Nature Methods* 10 (12): 1209–10.

Li, Marilyn M., Michael Datto, Eric J. Duncavage, Shashikant Kulkarni, Neal I. Lindeman, Somak Roy, Apostolia M. Tsimberidou, et al. 2017. “Standards and Guidelines for the Interpretation and Reporting of Sequence Variants in Cancer: A Joint Consensus Recommendation of the Association for Molecular Pathology, American Society of Clinical Oncology, and College of American Pathologists.” *The Journal of Molecular Diagnostics: JMD* 19 (1): 4–23.

Richards, Sue, Nazneen Aziz, Sherri Bale, David Bick, Soma Das, Julie Gastier-Foster, Wayne W. Grody, et al. 2015. “Standards and Guidelines for the Interpretation of Sequence Variants: A Joint Consensus Recommendation of the American College of Medical Genetics and Genomics and the Association for Molecular Pathology.” *Genetics in Medicine: Official Journal of the American College of Medical Genetics* 17 (5): 405–24.

Robarge, J. D., L. Li, Z. Desta, A. Nguyen, and D. A. Flockhart. 2007. “The Star-Allele Nomenclature: Retooling for Translational Genomics.” *Clinical Pharmacology and Therapeutics* 82 (3): 244–48.

Tate, John G., Sally Bamford, Harry C. Jubb, Zbyslaw Sondka, David M. Beare, Nidhi Bindal, Harry Boutselakis, et al. 2019. “COSMIC: The Catalogue Of Somatic Mutations In Cancer.” *Nucleic Acids Research* 47 (D1): D941–47.

Wagner, Alex H., Adam C. Coffman, Benjamin J. Ainscough, Nicholas C. Spies, Zachary L. Skidmore, Katie M. Campbell, Kilannin Krysiak, et al. 2016. “DGIdb 2.0: Mining Clinically Relevant Drug–gene Interactions.” *Nucleic Acids Research* 44 (D1): D1036–44.

Xin, Jiwen, Adam Mark, Cyrus Afrasiabi, Ginger Tsueng, Moritz Juchler, Nikhil Gopal, Gregory S. Stupp, et al. 2015. “MyGene.info and MyVariant.info: Gene and Variant Annotation Query Services.” *bioRxiv*. https://doi.org/10.1101/035667.
